# Supplementary material for: Radiogenic strontium isotope variability in the Valley of Oaxaca: A predictive isoscape for Mesoamerican paleomobility studies
Source: PLoS One. 2025 Dec 10;20(12):e0338628. doi: 10.1371/journal.pone.0338628 (PMC12694802; doi:10.1371/journal.pone.0338628)
Supplement: S1 Appendix — (PDF) [file pone.0338628.s004.pdf]

S1 Appendix. Identification of contamination from agricultural fertilizers in modern plant samples from San Martín Tilcajete

In this manuscript, we use modern plant samples from across the Valley of Oaxaca (*n*=95) to test the performance of an initial global isoscape model trained on isotope data compiled from previously published global and regional syntheses [1–4]. We then use the Oaxaca plant sample data to train subsequent iterations of the model to develop a regionally specific predictive isoscape model of $^{87}Sr/^{86}Sr$ values across Mesoamerica. During our analysis of uncertainty patterns for predictions across the isoscape, we detected a group of samples from San Martín Tilcajete (*n*=12) with implausibly high $^{87}Sr/^{86}Sr$ rations for the region.

The Tilcajete samples' $^{87}Sr/^{86}Sr$ values ranged from 0.707237 to 0.711976, with a mean of $^{87}Sr/^{86}Sr$=0.709283±0.001535 (1σ, *n*=12). This is a remarkably wide range of $^{87}Sr/^{86}Sr$ values that is not at all typical of other sites included in the analysis. Most sites have narrow interquartile ranges (Fig S1.1) and standard deviations (≤ E-4), whereas both Tilcajete's interquartile range and standard deviation (E-3) are an order of magnitude larger. When all Tilcajete samples are excluded from the analysis, the total $^{87}Sr/^{86}Sr$ range for the Valley of Oaxaca shrinks by over 50% to a range of just 0.704603 – 0.708782. Although the mean of all observed samples decreases only slightly to $^{87}Sr/^{86}Sr$=0.706500±0.001001 (1σ, *n*=83), the standard deviation decreases by an order of magnitude.

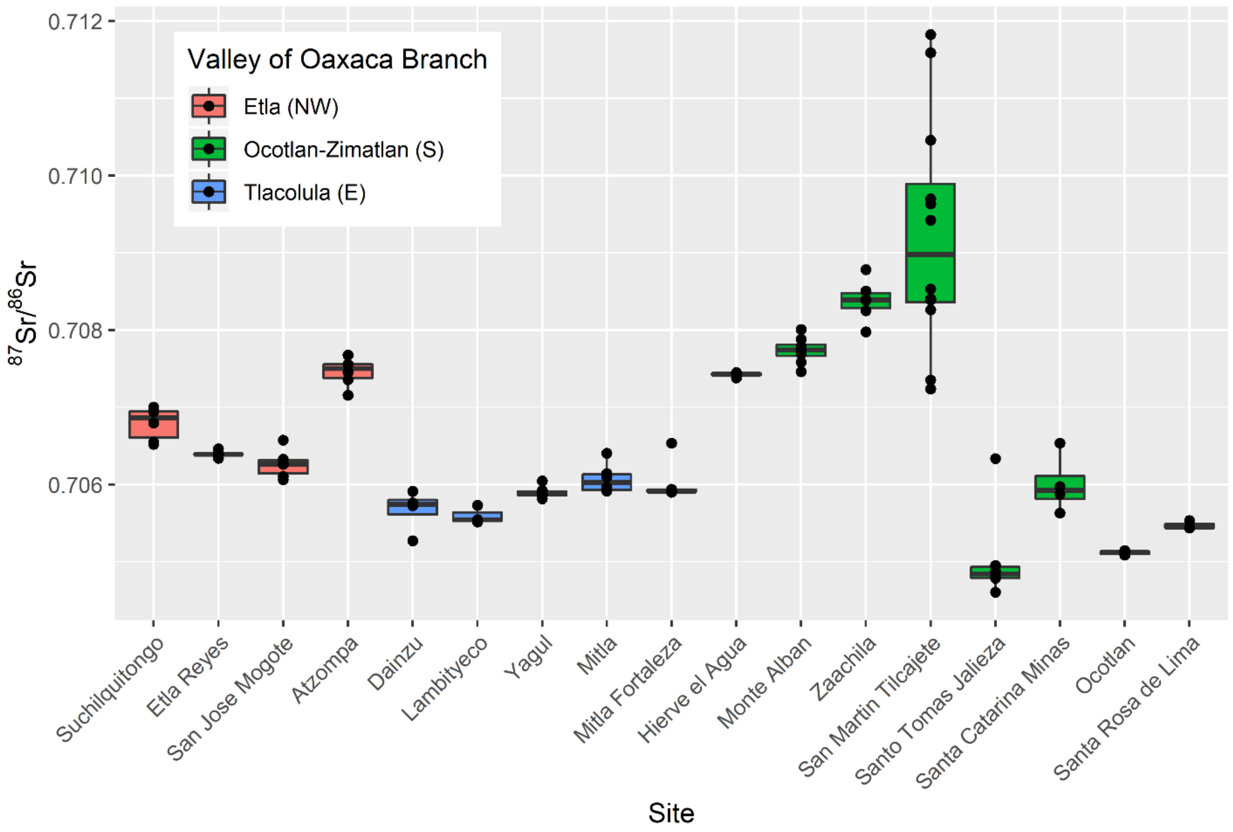

Fig S1.1. Boxplots of $^{87}Sr/^{86}Sr$ ranges for all Valley of Oaxaca sites overlain with individual plant samples. Note the markedly larger range of Tilcajete compared to all other sites.

The Tilcajete samples include the six highest $^{87}Sr/^{86}Sr$ values in the analysis. This is unexpected, given that Tilcajete is located on relatively young geologic bedrock: a Neogene andesitic tuff/andesite deposit neighbored by Quaternary alluvial deposits to the west, intrusive dacitic and andesitic porphyritic igneous rock deposits to the east, and Lower Cretaceous sandstone-shale deposits to the north [5]. Tilcajete's location near a geologic contact zone could account for the wider range in observed $^{87}Sr/^{86}Sr$ values. Our regionally specific Mesoamerican isoscape produced higher variability in $^{87}Sr/^{86}Sr$ predictions (associated with greater uncertainty) in areas near major bedrock transitions due to the greater variability in potential source Sr in these areas. At Tilcajete, however, the oldest neighboring geology dates to the Lower Cretaceous. This is still quite young when compared to the reported maximum value at Tilcajete of $^{87}Sr/^{86}Sr=0.711976$. Most observed Mesoamerican $^{87}Sr/^{86}Sr$ values fall under ~0.7090 [2,6]. Indeed, Tilcajete's maximum value falls within the range of the highest $^{87}Sr/^{86}Sr$ values observed in all Mesoamerica—from ~0.7110-0.7150, observed in the Maya Mountains, a significantly older Paleozoic formation in southern Belize [7].

After identifying the Tilcajete plant samples (n=12) as outliers that were unlikely to reflect the geologic inputs to local $^{87}Sr/^{86}Sr$ values, we investigated the collection area further. We found that although the plant samples we collected were not from an actively farmed zone, the plot of land had been intensively farmed in the last 50 years. The site, located on the slope of a hill, was leveled via the addition of foreign soil that would have impacted local $^{87}Sr/^{86}Sr$ values in modern plants. Additionally, the landowner confirmed that when his family had previously farmed the plot, they used agricultural fertilizers which are also known to skew local $^{87}Sr/^{86}Sr$ values away from underlying bedrock values. As the Tilcajete samples thus most likely did not reflect geologic or regularly occurring environmental Sr inputs, we excluded them from the dataset used to train the final $^{87}Sr/^{86}Sr$ isoscape model.

References

1. Bataille CP, Crowley BE, Wooller MJ, Bowen GJ. Advances in global bioavailable strontium isoscapes. Palaeogeography, Palaeoclimatology, Palaeoecology. 2020;555: 109849. doi:10.1016/j.palaeo.2020.109849

2. Ebert CE, Hixon SW, Buckley GM, George RJ, Pacheco-Forés SI, Palomo JM, et al. The Caribbean and Mesoamerica Biogeochemical Isotope Overview (CAMBIO). Sci Data. 2024;11: 349. doi:10.1038/s41597-024-03167-6

3. Le Corre M, Dargent F, Grimes V, Wright J, Côté SD, Reich MS, et al. An ensemble machine learning bioavailable strontium isoscape for Eastern Canada. Geiss CE, editor. FACETS. 2025;10: 1–17. doi:10.1139/facets-2024-0180

4. Wang X, Bocksberger G, Arandjelovic M, Agbor A, Angedakin S, Aubert F, et al. Strontium isoscape of sub-Saharan Africa allows tracing origins of victims of the transatlantic slave trade. Nat Commun. 2024;15: 10891. doi:10.1038/s41467-024-55256-0

5. Jiménez Hernández A, Mendoza Torres A. Carta geológico-minera Villa de Zaachila E14-D57 Oaxaca. Pachuca, Hidalgo: Servicio Geológico Mexicano; 2009.

6. Price TD, Burton JH, Fullagar PD, Wright LE, Buikstra JE, Tiesler V. Strontium isotopes and the study of human mobility in ancient Mesoamerica. Latin American Antiquity. 2008;19: 167–180. doi:10.2307/25478222

7. Freiwald C. Maya migration networks: Reconstructing population movement in the Belize River valley during the Late and Terminal Classic. Ph.D., The University of Wisconsin - Madison. 2011. Available: https://www.proquest.com/docview/886460450/abstract/F82F1B17A9DA43E7PQ/1

S1 Apéndice. Identificación de contaminación por fertilizantes agrícolas en muestras de plantas modernas de San Martín Tilcajete

En este manuscrito, utilizamos muestras de plantas modernas del Valle de Oaxaca (*n*=95) para evaluar el rendimiento de un modelo inicial de isopaisaje global, entrenado con datos isotópicos recopilados a partir de síntesis globales y regionales publicadas previamente [1–4]. Posteriormente, utilizamos los datos de las muestras de plantas de Oaxaca para entrenar iteraciones posteriores del modelo y desarrollar un modelo predictivo regional de isopaisaje con valores de $^{87}Sr/^{86}Sr$ en Mesoamérica. Durante nuestro análisis de los patrones de incertidumbre para las predicciones en el isopaisaje, detectamos un grupo de muestras de San Martín Tilcajete (*n*=12) con proporciones de $^{87}Sr/^{86}Sr$ inverosímilmente altas para la región.

Los valores de $^{87}Sr/^{86}Sr$ en las muestras de Tilcajete oscilaron entre 0.707237 y 0.711827, con una media de $^{87}Sr/^{86}Sr$ = 0.709233 ± 0.001495 (1σ, *n*=12). Este rango de valores de $^{87}Sr/^{86}Sr$ es notablemente amplio, a diferencia de otros sitios incluidos en el análisis. La mayoría de los sitios presentan rangos intercuartiles estrechos (Fig. S1.1) y desviaciones estándar (≤ E-4), mientras que tanto el rango intercuartil como la desviación estándar (E-3) de Tilcajete son un orden de magnitud mayores. Al excluir del análisis todas las muestras de Tilcajete, el rango total de $^{87}Sr/^{86}Sr$ para el Valle de Oaxaca se reduce en más del 50%, a un rango de tan solo 0.704603 – 0.708782. Aunque la media de todas las muestras observadas disminuye sólo ligeramente a $^{87}Sr/^{86}Sr$ =0.706467 ±0.000987 (1σ, *n*=83), la desviación estándar disminuye en un orden de magnitud.

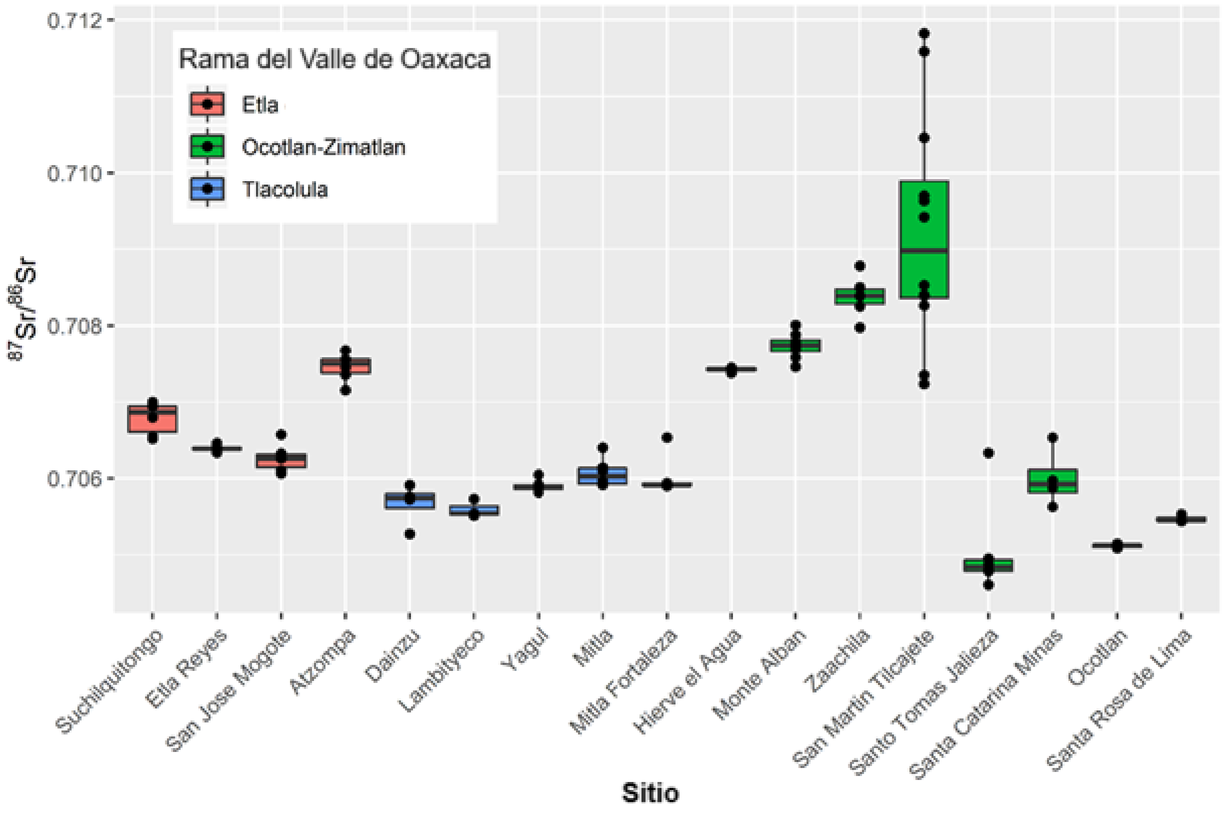

Fig. S1.1. Diagramas de caja de los rangos de $^{87}Sr/^{86}Sr$ para todos los sitios del Valle de Oaxaca, superpuestos con muestras de plantas individuales. Obsérvese el rango considerablemente mayor de Tilcajete en comparación con los demás sitios.

Las muestras de Tilcajete incluyen los seis valores más altos de $^{87}Sr/^{86}Sr$ en el análisis. Esto es inesperado, dado que Tilcajete se encuentra en un lecho rocoso geológico relativamente joven: un depósito de toba/andesita andesítica neógena contigua a depósitos aluviales cuaternarios al oeste, depósitos de roca ígnea porfídica dacítica e intrusiva andesítica al este, y depósitos de arenisca-lutita del Cretácico Inferior al norte [5]. La ubicación de Tilcajete cerca de una zona de contacto geológico podría explicar el rango más amplio en los valores observados de $^{87}Sr/^{86}Sr$. Nuestro isopaisaje mesoamericano regionalmente específico produjo una mayor variabilidad en las predicciones de $^{87}Sr/^{86}Sr$ (asociada con una mayor incertidumbre) en áreas cercanas a las principales transiciones del lecho rocoso debido a la mayor variabilidad en la fuente potencial de Sr en estas áreas. En Tilcajete, sin embargo, la geología vecina más antigua data del Cretácico Inferior. Este valor es aún bastante reciente en comparación con el valor máximo reportado en Tilcajete de $^{87}Sr/^{86}Sr=0.711827$. La mayoría de los valores de $^{87}Sr/^{86}Sr$ observados en Mesoamérica se encuentran por debajo de ~0.7090 [2,6]. De hecho, el valor máximo de Tilcajete se encuentra dentro del rango de los valores más altos de $^{87}Sr/^{86}Sr$ observados en toda Mesoamérica, de ~0.7110 a 0.7150, observados en las Montañas Mayas, una formación paleozoica significativamente más antigua en el sur de Belice [7].

Tras identificar las muestras de plantas de Tilcajete (n=12) como valores atípicos que probablemente no reflejaran las aportaciones geológicas a los valores locales de $^{87}Sr/^{86}Sr$, investigamos el área de recolección con más profundidad. Descubrimos que, si bien las muestras de plantas que recolectamos no provenían de una zona de cultivo activo, la parcela había sido cultivada intensivamente en los últimos 50 años. El sitio, ubicado en la ladera de una colina, fue nivelado mediante la adición de tierra extraña que habría afectado a los valores locales de $^{87}Sr/^{86}Sr$ en las plantas modernas. Además, el propietario del terreno confirmó que cuando su familia había cultivado previamente la parcela, utilizaban fertilizantes agrícolas que también se sabe que distorsionan los valores locales de $^{87}Sr/^{86}Sr$ de los valores del lecho rocoso subyacente. Como las muestras de Tilcajete probablemente no reflejaban aportaciones geológicas ni ambientales de Sr que ocurren regularmente, las excluimos del conjunto de datos utilizado para entrenar el modelo final de isopaisaje de $^{87}Sr/^{86}Sr$.

Referencias

1. Bataille CP, Crowley BE, Wooller MJ, Bowen GJ. Advances in global bioavailable strontium isoscapes. Palaeogeography, Palaeoclimatology, Palaeoecology. 2020;555: 109849. doi:10.1016/j.palaeo.2020.109849

2. Ebert CE, Hixon SW, Buckley GM, George RJ, Pacheco-Forés SI, Palomo JM, et al. The Caribbean and Mesoamerica Biogeochemical Isotope Overview (CAMBIO). Sci Data. 2024;11: 349. doi:10.1038/s41597-024-03167-6

3. Le Corre M, Dargent F, Grimes V, Wright J, Côté SD, Reich MS, et al. An ensemble machine learning bioavailable strontium isoscape for Eastern Canada. Geiss CE, editor. FACETS. 2025;10: 1–17. doi:10.1139/facets-2024-0180

4. Wang X, Bocksberger G, Arandjelovic M, Agbor A, Angedakin S, Aubert F, et al. Strontium isoscape of sub-Saharan Africa allows tracing origins of victims of the transatlantic slave trade. Nat Commun. 2024;15: 10891. doi:10.1038/s41467-024-55256-0

5. Jiménez Hernández A, Mendoza Torres A. Carta geológico-minera Villa de Zaachila E14-D57 Oaxaca. Pachuca, Hidalgo: Servicio Geológico Mexicano; 2009.

6. Price TD, Burton JH, Fullagar PD, Wright LE, Buikstra JE, Tiesler V. Strontium isotopes and the study of human mobility in ancient Mesoamerica. Latin American Antiquity. 2008;19: 167–180. doi:10.2307/25478222

7. Freiwald C. Maya migration networks: Reconstructing population movement in the Belize River valley during the Late and Terminal Classic. Ph.D., The University of Wisconsin - Madison. 2011. Available: https://www.proquest.com/docview/886460450/abstract/F82F1B17A9DA43E7PQ/1
