# Supplementary material for: Radiogenic strontium isotope variability in the Valley of Oaxaca: A predictive isoscape for Mesoamerican paleomobility studies
Source: PLoS One. 2025 Dec 10;20(12):e0338628. doi: 10.1371/journal.pone.0338628 (PMC12694802; doi:10.1371/journal.pone.0338628)
Supplement: S2 Appendix — (PDF) [file pone.0338628.s005.pdf]

Variabilidad isotópica del estroncio radiogénico en el Valle de Oaxaca: un isopaisaje predictivo para estudios de paleomovilidad mesoamericana

Sofía I. Pacheco-Forés[1¶*], Nicolas Gauthier[2¶*], Lacey B. Carpenter[3], Gwyneth Gordon[4], Kelly J. Knudson[5]

[1]Departmento de Antropología, Universidad de Minnesota, Twin Cities, Minneapolis, Minnesota, Estados Unidos
[2]Museo de Historia Natural de Florida, Universidad de Florida, Gainesville, Florida, Estados Unidos
[3]Departmento de Antropología, Universidad Estatal de Nueva York, Buffalo, Buffalo, Nueva York, Estados Unidos
[4]Escuela de Exploración de la Tierra y del Espacio, Universidad Estatal de Arizona, Tempe, Arizona, Estados Unidos
[5]Centro de Investigaciones Bioarqueológicas, Escuela de la Evolución Humana y del Cambio Social, Universidad Estatal de Arizona, Tempe, Arizona, Estados Unidos

*Autores correspondientes
Emails: sipf@umn.edu (SIPF); nicolas.gauthier@ufl.edu (NG)

[¶]Estos autores contribuyeron igualmente a este trabajo.

Resumen

El análisis isotópico del estroncio radiogénico ($^{87}Sr/^{86}Sr$) es un método bien establecido para reconstruir la movilidad de las poblaciones humanas en el pasado y el presente. Los datos de referencia de $^{87}Sr/^{86}Sr$ son fundamentales para el método, ya que el Sr varía a lo largo del paisaje según la geología local y factores geoambientales. Sin embargo, la aplicación del método dentro de los estudios de la paleomovilidad mesoamericana antigua se ha concentrado en dos regiones clave: Teotihuacan y la región maya, a pesar de su posible relevancia más amplia en toda Mesoamérica. Esto se debe en parte a la falta de datos de referencia de $^{87}Sr/^{86}Sr$ disponibles para la región en general. Utilizando el Valle de Oaxaca como caso de estudio, utilizamos Árboles de Regresión Aditiva Bayesiana (BART) para generar un modelo predictivo de isopaisaje de $^{87}Sr/^{86}Sr$ calibrado localmente de Mesoamérica en general y del Valle de Oaxaca en particular. Integramos (1) datos observados de $^{87}Sr/^{86}Sr$ de muestras de plantas modernas (n=95) de 17 sitios en todo el Valle, (2) una base de datos compilada de datos continentales de $^{87}Sr/^{86}Sr$ de América del Norte y del Sur, (3) mapas geológicos de lecho rocoso y (4) datos espaciales de alta resolución sobre covariables geoambientales de Sr para desarrollar y probar iterativamente un modelo predictivo de alto rendimiento para Mesoamérica, destacando la importancia de la calibración regional en el desarrollo de isopaisajes predictivos de $^{87}Sr/^{86}Sr$. Nuestros resultados indican que, aunque existe superposición, $^{87}Sr/^{86}Sr$ se puede utilizar para detectar la migración dentro del Valle de Oaxaca, así como entre el Valle y la mayor parte de Mesoamérica. Luego aplicamos nuestro isopaisaje a datos humanos previamente publicados de $^{87}Sr/^{86}Sr$ de Monte Albán, Oaxaca para demostrar cómo la cuantificación explícita de la incertidumbre de nuestro modelo en los rangos locales de $^{87}Sr/^{86}Sr$ permite una interpretación más matizada de la paleomovilidad en muestras arqueológicas.

Introducción

Existe una larga tradición de investigación arqueológica y bioarqueológica en el Valle de Oaxaca [1–5]. Gran parte de esta investigación se centra en el surgimiento de una de las primeras sociedades a nivel estatal en América: el estado zapoteca multiétnico de Monte Albán en el Valle de Oaxaca del sur de México durante el período Formativo Tardío (300-100 a.C.). Los arqueólogos han documentado cambios marcados en la cultura material y los patrones de asentamiento durante este período, lo que los lleva a inferir que la migración regional e interregional puede haber desempeñado un papel en el desarrollo del estado zapoteca [6]. Aunque los últimos 40 años han visto una explosión en los estudios biogeoquímicos de restos óseos humanos para probar directamente las hipótesis de la migración mesoamericana [7], estos estudios de paleomovilidad se han centrado abrumadoramente en el centro de México y las tierras bajas mayas centrales [8]. Existen muy pocos estudios biogeoquímicos de la migración en el Valle de Oaxaca [pero véanse 9–12]. Esta escasez de investigaciones biogeoquímicas de la migración en el Valle de Oaxaca puede deberse al menos en parte a la falta de datos de referencia isotópicos publicados tanto como la complejidad geológica de la región.

El desarrollo de mapas predictivos de la variación espacial isotópica ambiental, llamados isopaisajes, ha facilitado enormemente la investigación de paleomovilidad en regiones que carecen de datos de referencia isotópicos [13]. En este artículo, examinamos la variabilidad del estroncio radiogénico ($^{87}Sr/^{86}Sr$) en el Valle de Oaxaca. Presentamos valores de $^{87}Sr/^{86}Sr$ de un total de 95 muestras de plantas modernas de 17 sitios en todo el Valle de Oaxaca. Utilizamos el

conjunto de datos resultante, junto con datos de $^{87}Sr/^{86}Sr$ previamente compilados, para desarrollar y probar un modelo predictivo de isopaisajes adaptado a Mesoamérica en general y al Valle de Oaxaca en particular. Nuestro objetivo es proporcionar un isopaisaje de referencia predictivo de $^{87}Sr/^{86}Sr$ de alto rendimiento para su uso en estudios de paleomovilidad en el Valle de Oaxaca.

Evaluación de la migración a través de la biogeoquímica en el Valle de Oaxaca

El Valle de Oaxaca está conformado por una serie de tres valles interconectados que forman un área triangular en forma de Y (Fig. 1). La rama de Etla se extiende al noroeste, la rama de Tlacolula se extiende al este y la rama de Ocotlán-Zimatlán se extiende al sur. Durante la fase Rosario (700-500 a.C.) del periodo Formativo Medio, surgieron tres asentamientos importantes: San José Mogote, Yeguih y San Martín Tilcajete, en cada una de las tres ramas del Valle de Oaxaca. Monte Albán se fundó en el centro del Valle a principios de la fase Monte Albán I Temprano (500-300 a.C.) [1,3,14]. Monte Albán continuó expandiendo su influencia durante la fase Monte Albán I Tardío (300 a.C.-100 a.C.) y la fase Monte Albán II (100 a.C.-200 d.C.) [15,16].

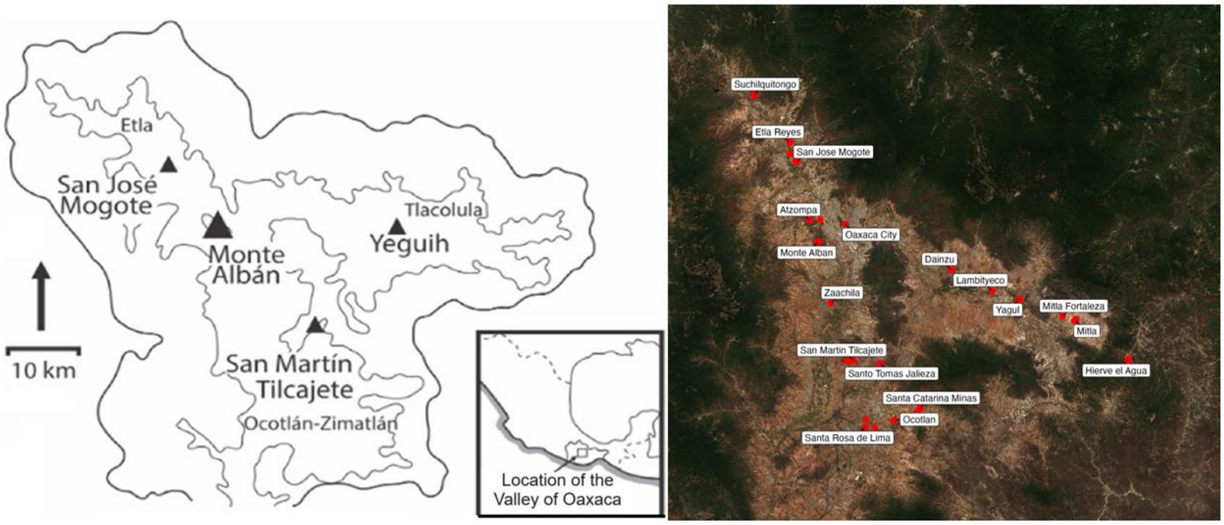

Fig. 1. El Valle de Oaxaca. Las tres ramas del Valle de Oaxaca y sus correspondientes centros regionales (izquierda) junto con un mapa de los 17 sitios del Valle de Oaxaca donde recolectamos plantas modernas para el análisis de $^{87}Sr/^{86}Sr$ (derecha). Mapa izquierdo modificado y reimpreso de [17] bajo licencia CC BY, con autorización de Carpenter, derechos de autora original 2019.

Tanto la migración regional como la interregional desempeñaron un papel crucial en los modelos de desarrollo sociopolítico del Valle de Oaxaca. El marcado crecimiento poblacional y los cambios demográficos dentro del Valle, coincidiendo con la fundación de Monte Albán, se han atribuido a la llegada de migrantes a la región, así como al movimiento de personas dentro del propio Valle [1,3,18–23]. La migración también se ha visto implicada en la resistencia de los centros de población rivales al expansionismo de Monte Albán [16,24]. Tras la consolidación del poder de Monte Albán, la migración fue esencial para las estrategias administrativas y las redes comerciales del estado [9,25–27]. A pesar de ello, la mayor parte de la evidencia arqueológica de

estas migraciones sigue siendo indirecta, insinuada a través de patrones de asentamiento, estimaciones de población y similitudes estilísticas en la arquitectura y la cultura material. Estas fuentes de datos pueden ofrecer información sobre el papel que desempeñó la migración en el desarrollo cultural de la región, pero la forma más directa de evaluarlo es primero identificar a los propios migrantes.

Los métodos biogeoquímicos permiten que los arqueólogos investiguen directamente la migración a escala individual. Los análisis de isótopos de hidrógeno (δ^{2}H), oxígeno estable (δ^{18}O), azufre (δ^{34}S), estroncio radiogénico (^{87}Sr/^{86}Sr) y plomo ($^{206/204}$Pb, $^{207/204}$Pb, $^{208/204}$Pb, $^{207/206}$Pb) proporcionan a los arqueólogos medios bien establecidos para reconstruir las historias residenciales de los pueblos del pasado [p. ej., 27–31]. Estos sistemas isotópicos varían geográficamente. Los isótopos de hidrógeno y oxígeno varían según la hidrología regional y reflejan factores ambientales como la altitud, la temperatura, la humedad y la latitud [28,33–36]. Por lo contrario, las proporciones de isótopos de azufre, estroncio radiogénico y plomo varían según la geología local, lo que refleja la edad y la composición del lecho rocoso geológico [37–41]. Estos isótopos se incorporan a los tejidos humanos calcificados a través del consumo de alimentos, la ingestión de líquidos y la inhalación de partículas de polvo, y reflejan la firma isotópica de la región donde vivió un individuo durante el desarrollo tisular [35,42–48]. Por lo tanto, los arqueólogos pueden detectar migrantes individuales al identificar cambios en las firmas isotópicas entre tejidos que se forman en diferentes momentos a lo largo de la vida y/o divergencias entre las firmas isotópicas de estos tejidos y el entorno funerario, ya que estas diferencias indicarían la residencia en regiones isotópicamente distintas [p. ej., 10,48].

Si bien los análisis biogeoquímicos de la paleomovilidad están bien establecidos y han tenido mucho éxito en la detección de la migración a lo largo de la antigua Mesoamérica, la mayoría de los estudios se centran en la ciudad de Teotihuacán, en el centro de México, y la región maya [8]. Hasta la fecha, solo unos pocos estudios han intentado identificar directamente a los migrantes en el Valle de Oaxaca, y todos, salvo uno, se han centrado exclusivamente en Monte Albán (Tabla 1). En conjunto, los cuatro estudios examinan la paleomovilidad de 75 individuos en el Valle de Oaxaca [9-12]. Solo el 8% de estos individuos fueron identificados como migrantes. Esta evidencia limitada de migración es sorprendente, ya que la discusión anterior indica que existe amplia evidencia arqueológica de migración regional e interregional en el Valle de Oaxaca.

Tabla 1. Estudios biogeoquímicos de la migración en el Valle de Oaxaca[a]

Sitio	Período	Método	*n* migrantes identificados/ *N* individuos muestreados	Fuente de datos
Monte Albán	500 a.C. – 1520 d.C.	δ^{18}O en fosfato de hueso	0/16	[12]
Monte Albán	100 a.C. – 550 d.C.	^{87}Sr/^{86}Sr en apatita de esmalte y hueso	1/5	[10]

Monte Albán	100 a.C. – 1350 d.C.	δ^{18}O en carbonato de esmalte	5/38	[9]
Teposcolula Yucundaa	1295-1634 d.C.	δ^{18}O en carbonato de esmalte	0/16	[11]

[a]Esta tabla solo incluye estudios de individuos excavados en contextos arqueológicos seguros. No incluye datos de Olivares Flores [50], ya que dicho análisis utiliza el análisis de δ^{18}O del carbonato de esmalte de *n*=7 cráneos incisos de procedencia desconocida para argumentar que seis de los siete individuos probablemente provienen del Valle de Oaxaca.

Identificación de migrantes mediante datos de referencia biogeoquímicas e isopaisajes

La escasez de migrantes identificados biogeoquímicamente en el Valle de Oaxaca, a pesar de la larga historia de investigación arqueológica en la región y su estatus como una de las primeras sociedades multiétnicas a nivel estatal en surgir en las Américas, puede deberse a la falta de datos de referencia biogeoquímicos publicados. Las referencias biogeoquímicas son esenciales para establecer expectativas para caracterizar las firmas isotópicas "locales" [51]. Si los valores isotópicos observados de un individuo divergen lo suficiente de una referencia biogeoquímica "local", el individuo se designa como "no local" y, por lo tanto, muy probablemente un migrante. Estas referencias biogeoquímicas se crean a través del análisis de muestras ambientales (típicamente de plantas o fauna) de un sitio de interés y la aplicación de estadísticas de resumen básicas para establecer umbrales para la variación local de ^{87}Sr/^{86}Sr [37,51]. Los arqueólogos han utilizado el valor promedio de ^{87}Sr/^{86}Sr de muestras ambientales de un sitio ± dos desviaciones estándar [52,53] o su rango intercuartil [54] para crear referencias biogeoquímicas que definen la variación local de ^{87}Sr/^{86}Sr en un sitio en particular.

Sin embargo, ninguno de los estudios biogeoquímicos previos de paleomovilidad en el Valle de Oaxaca emplea referencias biogeoquímicas. En cambio, identifican valores atípicos estadísticos entre los valores humanos observados como posibles migrantes. Si bien existe una abundancia de datos isotópicos ambientales publicados para otras regiones de México [p. ej., 54,55], dichos datos son escasos para el Valle de Oaxaca. Esto dificulta la creación de referencias biogeoquímicas regionales relevantes. Además, aunque las referencias biogeoquímicas proporcionan un medio objetivo para inferir el estado de movilidad de un individuo en relación con los valores locales esperados de ^{87}Sr/^{86}Sr, el medio estadístico para establecer umbrales que delimitaran la variación local es arbitrario [51] y no considera la distribución no normal de los valores de ^{87}Sr/^{86}Sr en el paisaje [54]. Por lo tanto, los estudios [9-12] citados en la Tabla 1 representan un inicio prometedor para investigar el papel de la migración en el desarrollo cultural del Valle de Oaxaca y el estado zapoteco, pero aún queda trabajo por hacer.

Isopaisajes e investigaciones de la paleomovilidad en el Valle de Oaxaca

En respuesta a la cobertura regional desigual de los datos de referencia biogeoquímicos publicados y las limitaciones de los enfoques de umbral para las referencias biogeoquímicas, los investigadores han adoptado cada vez más el uso de isopaisajes para completar los vacíos. Los

isopaisajes son mapas predictivos de la variación espacial isotópica ambiental [13]. Algunos isopaisajes se generan a través de la interpolación geoestadística (p. ej., kriging) de datos isotópicos observados [57–61]. Los isopaisajes geoestadísticos se basan en la autocorrelación espacial, la idea de que los puntos geográficamente más cercanos entre sí en un mapa tienden a ser ambientalmente (para H y O) o geológicamente (para S, Sr y Pb) similares. Además de utilizar datos isotópicos empíricos, estos modelos geoestadísticos pueden incorporar covariables como mapas hidrológicos o geológicos para restringir aún más la variabilidad isotópica prevista. Sin embargo, dado que los isótopos suelen tener una distribución irregular en el paisaje, estos isopaisajes geoestadísticos pueden representar erróneamente la variabilidad real del sistema isotópico seleccionado [62]. Por lo tanto, deben utilizarse con precaución.

Los isopaisajes geoestadísticos que utilizan valores empíricos de δ^{18}O de muestras de agua tomadas en todo México muestran el rango previsto de variabilidad clinal de δ^{18}O en México [63,64]. Moreiras Reynaga y colegas [65] dividen esta variación clinal en cinco zonas distintas o ligeramente superpuestas de valores de δ^{18}O. La zona que contiene el Valle de Oaxaca cubre una gran franja del oeste de México, incluyendo la mayor parte de la costa del Pacífico mexicano [65]. El hecho de que un área geográfica tan masiva comparta valores de δ^{18}O similares significa que existe una alta probabilidad de no poder identificar migrantes potenciales en el Valle de Oaxaca de otras partes de la costa del Pacífico. La incapacidad de los análisis de δ^{18}O para abordar cuestiones de movilidad regional dentro del Valle de Oaxaca y su utilidad limitada para evaluar la movilidad interregional en gran parte del occidente de México ha llevado a algunos académicos a postular que los isótopos de δ^{18}O pueden no tener un rango de variación suficiente para detectar la presencia de individuos no locales dentro del Valle [9,50]. Esto podría explicar por qué tres de los cuatro estudios de paleomovilidad existentes en el Valle de Oaxaca que utilizan δ^{18}O identifican tan pocos migrantes (Tabla 1). La focalización en otros sistemas isotópicos con mayor variabilidad en el Valle de Oaxaca y/o el uso de múltiples sistemas isotópicos en lugar de depender de un solo sistema representan la mejor vía para los estudios biogeoquímicos de la migración en el Valle de Oaxaca.

Los modelos mecanicistas representan otro enfoque para generar isopaisajes. Este método emplea los principios básicos de la geoquímica isotópica para predecir la evolución de un sistema isotópico específico en una dada región [67]. Las predicciones del modelo se comparan con los datos de referencia isotópicos publicados para evaluar su rendimiento [68–71]. Si bien son prometedores, los isopaisajes de modelos mecanicistas se ven limitados por mapas geológicos insuficientemente detallados, inexactos o inconsistentes que, a su vez, producen modelos de bajo rendimiento. Además, las estimaciones a partir de mapas geológicos deben tener en cuenta la meteorización del suelo, la profundidad de las raíces de las plantas y la variación vertical del suelo, así como la biodisponibilidad relativa de elementos que causarán una modificación significativa de los isótopos de estroncio entre la litología subyacente y las poblaciones que viven en la región. La meteorización y la variación vertical del suelo dependen en gran medida del clima. La interacción entre estos parámetros de control es poco conocida, lo que dificulta la elaboración de modelos predictivos a partir de factores puramente mecanicistas.

Los desafíos que plantean los isopaisajes geoestadísticos empíricos y los modelos mecanicistas han dado lugar a un enfoque estadístico híbrido basado en procesos para su creación. Este método utiliza la regresión de bosque aleatorio, un algoritmo de aprendizaje automático basado

en árboles de decisión. Un árbol de decisión funciona dividiendo repetidamente los datos en función de los valores predictores (como la edad del lecho rocoso o la pluviosidad) para crear grupos cada vez más homogéneos. Por ejemplo, un árbol simple podría dividir primero las muestras en función de la edad del lecho rocoso (más o menos de 100 millones de años), luego dividir aún más cada grupo en función de la elevación, y así sucesivamente hasta que pueda predecir con precisión los valores de estroncio. Los árboles de decisión son intuitivos, pero pueden ser inestables y propensos al sobreajuste cuando se utilizan solos. Los bosques aleatorios y métodos similares abordan esta limitación combinando cientos de árboles, cada uno considerando diferentes combinaciones de predictores. Este enfoque de conjunto produce predicciones más robustas que un solo árbol. Los isopaisajes híbridos basados en procesos incorporan datos de referencia isotópicos empíricos, junto con otras covariables geoambientales utilizadas en isopaisajes geoestadísticos, en el marco de modelado mecanístico para predecir los valores isotópicos [62,67,72,73]. Este enfoque híbrido supera simultáneamente las limitaciones de los modelos de isopaisajes geoestadísticos y mecanísticos, generando modelos de mayor rendimiento que consideran con precisión las distribuciones no normales de isótopos en las regiones de interés objetivo.

Cuantificar la incertidumbre de las predicciones de isopaisajes es esencial para distinguir entre individuos locales y no locales en contextos arqueológicos [74]. Sin embargo, los isopaisajes generados mediante regresión de bosque aleatorio no proporcionan inherentemente estimaciones de incertidumbre para sus predicciones de valores isotópicos locales. Para abordar esta deficiencia, investigadores han ocupado los bosques de regresión cuantil para generar mapas espacialmente explícitos de incertidumbre para acompañar los isopaisajes predictivos [62,75,76]. Este método estima cuantiles condicionales con base en la distribución de resultados de observaciones similares en todos los árboles del bosque, lo que proporciona estimaciones de la incertidumbre asociada con la predicción de un valor isotópico particular [77]. Sin embargo, es posible que este enfoque subestimará la incertidumbre en regiones con datos de entrenamiento escasos, como regiones geológicamente complejas y con bajo índice de muestreo, como el Valle de Oaxaca.

Isótopos de estroncio y el Valle de Oaxaca

El Valle de Oaxaca representa un candidato particularmente atractivo para un isopaisaje estadístico de $^{87}Sr/^{86}Sr$ basado en procesos. El estroncio radiogénico biodisponible varía según la edad y la composición del lecho rocoso local, pero factores como la erosión y la meteorización preferencial del lecho rocoso, así como la adición de material proveniente del polvo eólico y la espuma marina, pueden alterar significativamente los valores de $^{87}Sr/^{86}Sr$ en el ambiente que finalmente se incorporan a los tejidos humanos [para una discusión detallada de la sistemática del Sr y el ciclo biodisponible del $^{87}Sr/^{86}Sr$ en los ecosistemas, véanse 36, 37, 77].

El Valle de Oaxaca se ubica en la Sierra Madre Sur, la provincia morfotectónica más compleja geológicamente y menos conocida de México [79]. Esta provincia contiene algunas de las formaciones geológicas metamórficas más antiguas de México: granulitas precámbricas, conocidas como el Complejo Oaxaqueño, que se desarrollaron a partir de una secuencia de rift continental [80]. Este terreno precámbrico, profundamente erosionado, está cubierto discordantemente por granitoides aislados del Paleozoico y Mesozoico, y forma el límite occidental del Valle de Oaxaca. Si bien el fondo del Valle está compuesto por depósitos aluviales

cuaternarios recientes, el borde noreste del Valle está formado por rocas de arenisca y lodolita del Cámbrico al Eoceno, atravesadas por la Falla de Oaxaca, y una serie de rocas metamórficas del Paleozoico al Jurásico, conocidas como el Complejo de la Sierra de Juárez. Una mezcla de tobas volcánicas andesíticas y riolíticas magmáticas del Cenozoico, junto con carbonatos de caliza-dolomita del Mesozoico, conforman el límite sureste del Valle. El límite sur del Valle está dominado por tobas andesíticas-riolíticas del Cenozoico, intercaladas con carbonatos del Mesozoico y afloramientos intrusivos de granitoides del Cenozoico [79,81–85]. La marcada variabilidad en la edad y composición del lecho rocoso subyacente, que constituye el material fuente de los depósitos aluviales cuaternarios en el Valle de Oaxaca y sus alrededores, augura una alta variación intra e interregional en los isótopos de $^{87}Sr/^{86}Sr$ (Fig. 2).

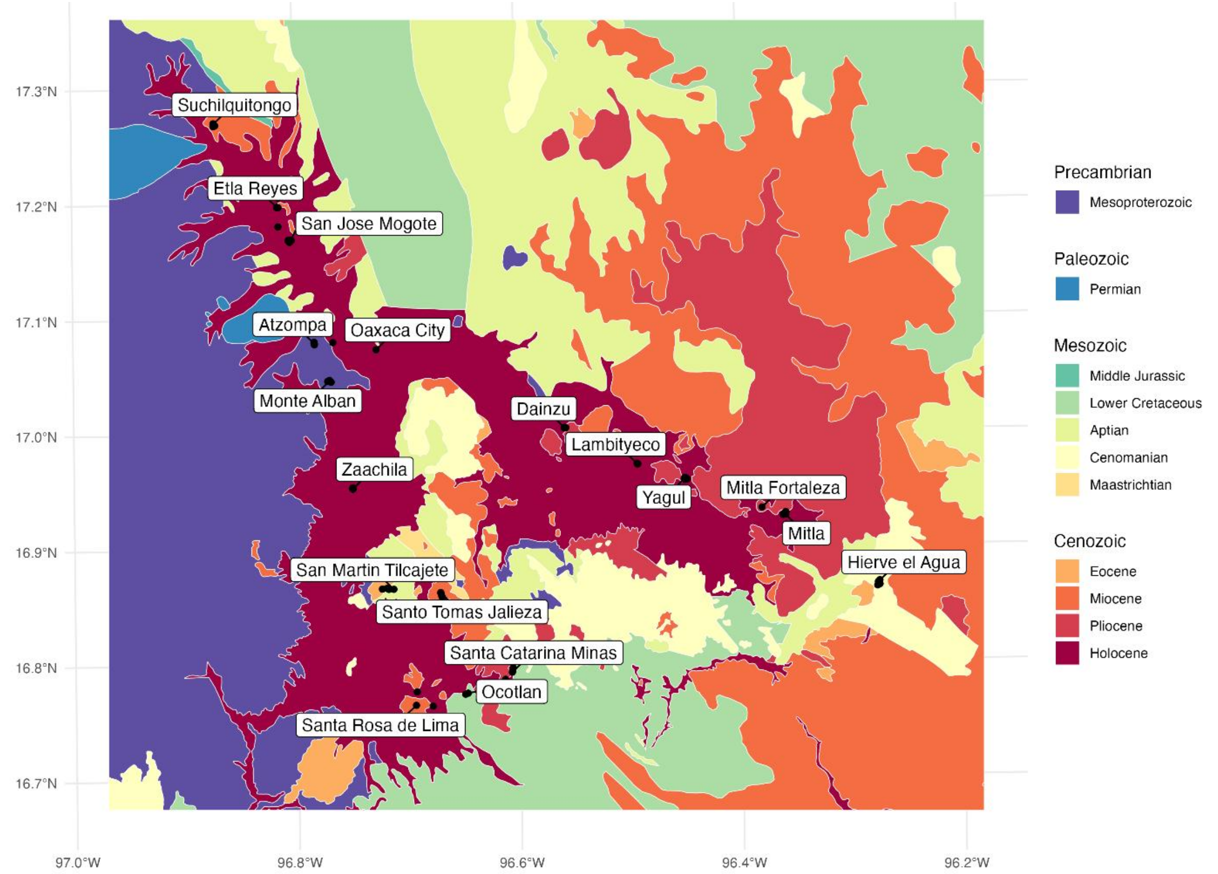

Fig. 2. Un mapa de la edad geológica del lecho rocoso subyacente en el Valle de Oaxaca.

Aunque no existen isopaisajes híbridos para ningún sistema isotópico en el Valle de Oaxaca, Bataille y colegas [67] presentan un modelo de isopaisaje $^{87}Sr/^{86}Sr$ que utiliza regresión de bosque aleatorio para predecir la variabilidad de $^{87}Sr/^{86}Sr$ biodisponible a nivel mundial. Su innovador modelo integra datos empíricos compilados de $^{87}Sr/^{86}Sr$ y covariables auxiliares que se sabe que afectan a $^{87}Sr/^{86}Sr$ biodisponible con un marco de modelo mecanicista basado en el lecho rocoso geológico. Bataille y colegas prueban el modelo utilizando datos empíricos de $^{87}Sr/^{86}Sr$. El modelo funciona bien en la predicción de valores observados de $^{87}Sr/^{86}Sr$ en regiones ricas en datos de referencia, pero funciona mal al predecir valores observados de $^{87}Sr/^{86}Sr$ en regiones con poco muestreo y geologías complejas. Los autores concluyen que la adición de tan solo decenas a cientos de puntos de datos de $^{87}Sr/^{86}Sr$ en áreas geológicamente

complejas y con un bajo índice de muestreo mejora significativamente la precisión regional del modelo global. Además, en algunos casos, es necesario un modelo de isopaisaje específico para cada región que evite los datos globales de $^{87}Sr/^{86}Sr$ biodisponibles para superar el fuerte sesgo predictivo de dichos modelos hacia regiones ricas en datos.

Un modelo de isopaisaje regionalmente específico podría ser invaluable para promover la investigación sobre paleomovilidad en el Valle de Oaxaca. Desafortunadamente, existen muy pocos datos empíricos de referencia de $^{87}Sr/^{86}Sr$ en el Valle de Oaxaca. Price y sus colegas proporcionan algunos promedios a nivel de sitio de los valores de $^{87}Sr/^{86}Sr$, pero todo el Valle está representado por solo 10 muestras de cinco sitios, y no se han publicado los datos individuales [54]. Para abordar esta brecha en la investigación, presentamos datos de referencia de $^{87}Sr/^{86}Sr$ para 95 muestras de plantas modernas de 17 sitios en todo el Valle de Oaxaca. Utilizamos este conjunto de datos, junto con una base de datos compilada de datos continentales de $^{87}Sr/^{86}Sr$ de América del Norte y del Sur, para entrenar y probar iterativamente un modelo regional de isopaisaje mesoamericano de $^{87}Sr/^{86}Sr$ centrado en el Valle de Oaxaca. Nuestro objetivo es proporcionar un isopaisaje predictivo $^{87}Sr/^{86}Sr$ calibrado localmente de alta calidad que sentará las bases para una mayor investigación biogeoquímica de la paleomovilidad en el Valle de Oaxaca.

Materiales y métodos

Recolección de muestras

Recolectamos de manera oportunista un total de 95 muestras de plantas modernas de 17 sitios arqueológicos en el Valle de Oaxaca, con un promedio de cinco muestras de plantas por sitio. Nos enfocamos en plantas para el muestreo de referencia ya que $^{87}Sr/^{86}Sr$ en plantas reflejan un promedio más consistente del $^{87}Sr/^{86}Sr$ biodisponible local en un ecosistema dado que los suelos completos, que pueden variar mucho incluso en un área pequeña debido a las distintas concentraciones de estroncio y perfiles de meteorización de minerales en el lecho rocoso subyacente [37]. Aun así, las plantas con distintas profundidades de enraizamiento tienen acceso a diferentes fuentes de Sr (es decir, lecho rocoso vs. horizontes de suelo fuertemente meteorizados vs. polvo transportado por el viento), introduciendo variación en sus valores observados de $^{87}Sr/^{86}Sr$ [86]. Como tal, muestreamos profundidades de enraizamiento tanto superficiales como profundas siempre que fue posible para dar cuenta de estas diferentes fuentes locales de $^{87}Sr/^{86}Sr$. Además, para simular mejor las fuentes de $^{87}Sr/^{86}Sr$ en las dietas prehispánicas, priorizamos el muestreo de taxones de plantas que contribuyeron a las dietas antiguas en el Valle de Oaxaca, como el maguey (*Agave* spp.) y las tunas (*Opuntia ficus*), o taxones de plantas con profundidades de enraizamiento similares a las de alimentos básicos antiguos como el maíz (*Zea mays*) que no pudimos muestrear [87,88]. Además, buscamos evitar el muestreo de plantas tratadas con fertilizantes o agua de riego, ya que estos podrían sesgar las firmas de estroncio biodisponible local con fuentes no locales de estroncio.

Las muestras recolectadas consistieron principalmente en hojas o vainas de semillas, no en plantas completas. Se registraron las coordenadas UTM (Universal Transverse Mercator) y la elevación de cada muestra de planta mediante un GPS portátil. El Instituto Nacional de Antropología e Historia (INAH) de México no requiere permisos específicos para recolectar muestras de plantas modernas en los sitios de estudio. Además, no se recolectaron especies de

plantas en peligro de extinción o protegidas para el estudio. Las muestras se importaron al Laboratorio de Química Arqueológica de la Universidad Estatal de Arizona con los permisos otorgados a Pacheco-Forés por el Servicio de Inspección de Sanidad Animal y Vegetal del Departamento de Agricultura de los Estados Unidos (permisos PCIP-17-00469 y PCIP-18-00287). Se incluye información adicional sobre las consideraciones éticas, culturales y científicas específicas de la inclusión en la investigación global en la Información complementaria (S1 Lista de verificación).

Métodos biogeoquímicos

Las muestras de plantas se prepararon en el Laboratorio de Química Arqueológica de la Universidad Estatal de Arizona. Cuando fue posible, se simularon dietas prehispánicas mediante el aislamiento y análisis manual de componentes comestibles de plantas secas [89]. Las muestras de plantas se prepararon enjuagadas con agua Millipore de 18.2 MΩ para eliminar la suciedad adherida y se incineraron en un horno durante 10 horas a 800°C. Aproximadamente 25 mg de ceniza vegetal se digirieron en 2 mL de ácido nítrico y clorhídrico concentrado (una parte de HNO_3 por tres partes de HCl) a 50°C durante 24 horas. Esta lixiviación agresiva no incluye ácido fluorhídrico, que descompondría la estructura tetraédrica de sílice de la mayoría de los minerales de silicato. La lixiviación deja gran parte del suelo en forma sólida mientras prioriza la liberación de estroncio biodisponible dentro de las plantas. La solución de lixiviación sobrenadante se evaporó y los precipitados de la muestra se redisolvieron en ácido nítrico concentrado y se diluyeron a una solución madre 2M.

Las muestras disueltas se analizaron en el Laboratorio de Análisis de Metales, Medio Ambiente y Terrestre (METAL) de la Universidad Estatal de Arizona. Se tomó una alícuota para el análisis de la concentración elemental en un espectrómetro de masas de plasma acoplado inductivamente cuadrupolo iCAP de Thermo Fisher Scientific (Q-ICP-MS). Posteriormente, se separó el estroncio con un sistema PrepFAST siguiendo el protocolo estándar de laboratorio descrito en [90]. El estroncio se aisló de la matriz de la muestra utilizando resina de intercambio iónico Sr-Ca (Parte CF-MC-SrCa-1000) suministrada por Elemental Scientific, Inc. y ácido nítrico ultrapuro 5 M (HNO_3). Para determinar la recuperación química, se midió el 22% de las muestras en el Q-ICP-MS antes y después de la purificación química. El rendimiento químico promedio fue del 85%.

La porción restante de cada corte de Sr del PrepFAST se secó en un vaso de precipitados de teflón y se digirió con HNO_3 concentrado y peróxido de hidrógeno (H_2O_2) al 30% para eliminar los compuestos orgánicos de la resina. Una vez digeridas, las muestras se secaron de nuevo y se reconstituyeron con HNO_3 0.32 M. Utilizando la información de concentración del Q-ICP-MS, las muestras se diluyeron con HNO_3 0.32 M hasta una concentración constante calculada de 50 ppb de Sr. Posteriormente, las muestras se analizaron en un espectrómetro de masas de plasma acoplado inductivamente multicolector Thermo-Finnigan Neptune (MC-ICP-MS).

Los datos se recopilaron midiendo 60 relaciones simultáneas, integrando 4.194 segundos cada una. Las muestras se corrigieron para los blancos en el pico y se corrigieron en línea las contribuciones de ^{84}Kr sobre ^{84}Sr y ^{86}Kr sobre ^{86}Sr utilizando una relación $^{83}Kr/^{84}Kr$ de 0.201750 y una relación $^{83}Kr/^{86}Kr$ de 0.664533, tras la corrección instrumental del sesgo de masa utilizando una relación normalizadora $^{88}Sr/^{86}Sr$ de 8.375209. Las muestras se analizaron en dos

sesiones analíticas diferentes. La sensibilidad típica fue de 36 V en ^{88}Sr con una solución de Sr de 50 ppb, con valores de ^{83}Kr de 0.0001 V. Los voltajes de ^{85}Rb para las muestras fueron típicamente <0,001 V debido a las bajas relaciones iniciales de Rb/Sr de las muestras y a la eficaz purificación química. Sin embargo, todos los datos se corrigieron por interferencia utilizando una relación $^{85}Rb/^{87}Rb$ de 2.588960, normalizada a $^{88}Sr/^{86}Sr$ como se indicó anteriormente. Los valores atípicos de la relación con dos desviaciones estándar fuera de la media se eliminaron mediante una rutina de corrección matemática 2D de Matlab escrita por Stephen Romaniello. Todos los datos se re-normalizaron a un valor de SRM 987 de 0.710255 usando estándares de horquillado para compensar por error instrumental y deriva de fondo de Kr. La precisión interna típica de dos errores estándar (EE) para $^{87}Sr/^{86}Sr$ fue ≤1e-5.

Las secuencias incluyeron estándares SRM 987 de concentración coincidente. El SRM 987 se ejecutó como estándar de horquillado con un valor medido de $^{87}Sr/^{86}Sr = 0.710256 \pm 0.000010$ (2σ, $n = 57$). Cada sesión analítica incluyó una secuencia que incorporaba el estándar SRM 987 en un rango de concentraciones variables para verificar la precisión de los valores de $^{87}Sr/^{86}Sr$ en las muestras; todos los valores reportados se encuentran dentro del rango de valores precisos de $^{87}Sr/^{86}Sr$ dentro del rango de error de los estándares de horquillado [91]. Además, se ejecutó SRM 987 dopado con calcio hasta una proporción Ca/Sr de 500 para simular la precisión de las proporciones isotópicas en muestras poco purificadas con bajos rendimientos. Se utilizó SRM 987, con una concentración del 50%, dopado con una relación Ca/Sr de 500, como estándar de control, con un valor medido de $^{87}Sr/^{86}Sr = 0.710252 \pm 0.000018$ (2σ, $n = 17$). El agua de mar de IAPSO (Ocean Scientific International Ltd., Havant, Reino Unido), como estándar de control secundario, obtuvo un valor medido de 0.709182 ± 0.000010 (2σ, $n = 13$), con un margen de error del valor publicado de 0.709182 ± 0.000004 [92]. El NIST 1400 purificado en paralelo con las muestras tuvo un valor medido de 0.713115 ±0.000010 (2σ, n=10), con un margen de error del valor publicado de 0.713150 ±0.000016 [93]. Aproximadamente el 5% de las muestras (n=8) se analizaron por triplicado, con una precisión promedio en las mediciones individuales de ±0.000009 (2σ).

Desarrollo y prueba del modelo isoscape

Recopilamos datos de isótopos de estroncio para América del Norte y del Sur a partir de síntesis globales y regionales existentes [8,67,72] y nuestras mediciones recientemente recopiladas en el Valle de Oaxaca. El conjunto de datos compilado incluyó 7,271 mediciones de humanos, animales, plantas, suelo y fuentes de agua en diversos entornos. Los datos humanos del Panorama Biogeoquímico de Isótopos del Caribe y Mesoamérica (CAMBIO, versión 1.2) se filtraron para excluir a los individuos identificados como no locales del conjunto de datos compilado [8]. Las muestras de agua presentan un desafío especial para el modelado de estroncio, ya que pueden reflejar señales no locales debido al transporte a través de cuencas hidrográficas [69,94–96]. En lugar de excluir estas muestras por completo, incorporamos una variable predictora categórica que indica el tipo de muestra (p. ej., agua, planta, etc.), lo que permite que el modelo maneje adecuadamente los diferentes perfiles de incertidumbre asociados con cada tipo de muestra.

Filtramos los datos para excluir muestras con valores de $^{87}Sr/^{86}Sr$ fuera del rango de 0.703 a 0.780, eliminando así posibles valores atípicos y enfocando el entrenamiento del modelo en el rango más relevante para la interpretación arqueológica en Mesoamérica [véase 8]. Aplicamos

una transformación logit a las proporciones de isótopos de estroncio, un enfoque estándar para variables acotadas que ayuda a normalizar la distribución y reduce la influencia de los valores extremos en el entrenamiento del modelo.

Reunimos un conjunto de predictores ambientales espaciales basados en las relaciones establecidas con los patrones de distribución de isótopos de estroncio [67,69]. Estos predictores incluyeron variables climáticas actualizadas de CHELSA V2.1 [97,98], propiedades del suelo a múltiples profundidades, como la densidad aparente y el pH [99], variables geológicas, incluyendo la edad del basamento y las clasificaciones litológicas [67,100,101], datos de deposición de aerosoles del reanálisis de MERRA-2 [102], y variables del terreno. Realizamos análisis de componentes principales específicos del dominio para reducir la dimensionalidad de estos predictores, creando un conjunto mínimo de variables climáticas, edáficas, geológicas y de aerosoles no correlacionadas. Este enfoque resultó en 24 variables predictoras para el modelo final. Los resultados detallados del PCA se presentan en la Fig. S1.

Empleamos un enfoque de Árboles de Regresión Aditiva Bayesiana (BART) [103] para modelar isopaisajes de estroncio. Los bosques aleatorios tradicionales utilizados en isopaisajes previos [p. ej., 66] presentan limitaciones para cuantificar la incertidumbre de la predicción, mientras que los bosques de regresión cuantil [p. ej., 61,74,75] subestiman la incertidumbre en regiones con datos de entrenamiento dispersos. BART aborda estas limitaciones extendiendo los métodos de árboles de decisión por conjuntos con enfoques estadísticos bayesianos. A diferencia de los bosques aleatorios tradicionales que utilizan estructuras de árbol fijas, BART trata las configuraciones de los árboles en sí mismas como inciertas, manteniendo las distribuciones de probabilidad sobre diferentes posibles arreglos de árboles. Este marco bayesiano permite a BART modelar explícitamente tanto la variabilidad natural en las proporciones isotópicas como la incertidumbre que surge del conocimiento limitado del modelo, a la vez que previene el sobreajuste mediante la regularización.

La principal ventaja de BART para aplicaciones arqueológicas reside en su capacidad para cuantificar de forma natural la incertidumbre, que aumenta adecuadamente en regiones con escasez de datos o geológicamente complejas. En lugar de proporcionar un único valor predicho para cada ubicación, BART genera un rango de valores plausibles con sus probabilidades asociadas. Este enfoque proporciona a los arqueólogos información crucial sobre la confianza relativa de los rangos locales de estroncio y, por consiguiente, la fiabilidad de la identificación de migrantes. En regiones con mayor incertidumbre de predicción, se deben considerar rangos más amplios para definir las firmas locales, mientras que las áreas con menor incertidumbre permiten determinaciones más precisas.

Evaluamos el rendimiento del modelo utilizando los valores de error cuadrático medio (RMSE) y R-cuadrado (R^2) en datos de prueba retenidos. El RMSE representa el error de predicción promedio en las mismas unidades que los datos ($^{87}Sr/^{86}Sr$), mientras que el R^2 representa la proporción de varianza en los datos observados explicada por el modelo. Nuestro enfoque de evaluación iterativa implicó, primero, entrenar un modelo con nuestro conjunto de datos compilado de América del Norte y del Sur, excluyendo todos los datos (compilados y observados) de Mesoamérica. Posteriormente, probamos la precisión de predicción de este modelo con datos compilados de Mesoamérica, utilizando este proceso para ajustar los

parámetros del modelo y evaluar su capacidad de extrapolación a una nueva región de interés. Posteriormente, reentrenamos este modelo con los datos compilados de Mesoamérica y lo probamos con las nuevas muestras de Oaxaca para evaluar su capacidad de extrapolación dentro de nuestra región de interés. Para el isopaisaje final, reentrenamos el modelo incorporando todos los datos compilados de América del Norte, Central y del Sur, y nuestras nuevas muestras del Valle de Oaxaca. Comparamos las predicciones del modelo y las métricas de rendimiento en cada paso del proceso para evaluar la estabilidad de las predicciones del modelo y el valor añadido de incluir muestras cada vez más locales en el conjunto de entrenamiento. Posteriormente, aplicamos este modelo final en toda el área de estudio del Valle de Oaxaca para crear predicciones espacialmente explícitas de los valores de $^{87}Sr/^{86}Sr$ con las estimaciones de incertidumbre asociadas. Evaluamos la importancia de las variables calculando la frecuencia con la que se utilizó cada variable en el modelo BART [103], lo que nos permitió identificar los principales factores ambientales que impulsan los patrones isotópicos de estroncio en la región (Fig. S1). El isopaisaje resultante proporciona tanto los valores predichos de $^{87}Sr/^{86}Sr$ como las estimaciones de incertidumbre asociadas a una resolución espacial de 1 km. Estas predicciones sirven como base para comparar muestras arqueológicas y distinguir a los individuos locales de los no locales en la región.

Resultados

Resultados biogeoquímicos

La Tabla 2 muestra los valores de $^{87}Sr/^{86}Sr$ observados en las plantas muestreadas. En el Valle de Oaxaca, los valores de $^{87}Sr/^{86}Sr$ oscilaron entre 0.704752 y 0.711976, con una media de $^{87}Sr/^{86}Sr=0.706851 \pm 0.001419$ (1σ, $n = 95$).

Tabla 2. Datos de procedencia, detalles de la muestra y valores de $^{87}Sr/^{86}Sr$ para las muestras de plantas analizadas del Valle de Oaxaca.

Núm. de laboratorio	Sitio	UTM-E[a]	UTM-N[a]	Elevación (msnm)	Especie	Profundidad de enraizamiento	$^{87}Sr/^{86}Sr$	2 SE
ACL-10588	Atzompa	736019	1889532	1817	*Agave* spp.	superficial	0.707597	0.000008
ACL-10589	Atzompa	736018	1889624	1983	*Yucca filifera*	profundo	0.707703	0.000008
ACL-10590	Atzompa	737791	1889737	1884	*Jacaranda mimosifolia*	superficial	0.707704	0.000012
ACL-10591	Atzompa	735986	1889778	1893	*Acacia farnesiana*	profundo	0.707504	0.000007
ACL-10592	Atzompa	735987	1889716	1884	*Opuntia ficus*	superficial	0.707301	0.000011
ACL-10593	Atzompa	735988	1889655	1710	*Jacaranda mimosifolia*	superficial	0.707822	0.000009
ACL-9642	Dainzú	760073	1881555	1617	*Poa* spp.	superficial	0.705759	0.000008
ACL-9643	Dainzú	760018	1881603	1615	*Opuntia ficus*	superficial	0.705911	0.000007
ACL-9644	Dainzú	759992	1881588	1611	*Prosopis* spp.	profundo	0.705724	0.000012
ACL-9645	Dainzú	760168	1881588	1637	*Agave* spp.	superficial	0.705270	0.000009
ACL-10598	Etla Reyes	732518	1903608	1640	*Opuntia ficus*	superficial	0.706611	0.000008
ACL-10599	Etla Reyes	732509	1904407	1640	*Prosopis* spp.	profundo	0.706530	0.000006
ACL-10600	Etla Reyes	732528	1902685	1640	*Prosopis* spp.	profundo	0.706536	0.000013
ACL-10601	Etla Reyes	732410	1902715	1643	*Opuntia ficus*	superficial	0.706549	0.000008
ACL-10602	Etla Reyes	732410	1902684	1643	*Opuntia ficus*	superficial	0.706487	0.000008
ACL-10603	Etla Reyes	732520	1900840	1640	*Agave* spp.	superficial	0.706546	0.000009
ACL-9629	Hierve el Agua	790184	1866831	1771	*Agave* spp.	superficial	0.707431	0.000013
ACL-9630	Hierve el Agua	790123	1866492	1711	*Agave* spp.	superficial	0.707377	0.000008
ACL-9631	Hierve el Agua	790343	1866610	1653	*Agave* spp.	superficial	0.707448	0.000011
ACL-9632	Hierve el Agua	790333	1866995	1764	*Agave* spp.	superficial	0.707428	0.000010
ACL-9639	Lambityeco	767086	1878105	1613	*Yucca filifera*	profundo	0.705545	0.000013
ACL-9640	Lambityeco	767040	1878109	1609	*Agave* spp.	superficial	0.705730	0.000009
ACL-9641	Lambityeco	767017	1878173	1604	*Prosopis* spp.	profundo	0.705515	0.000010
ACL-9633	Mitla	781307	1873516	1694	*Agave* spp.	superficial	0.705960	0.000010

ACL-9634	Mitla	781270	1873523	1703	*Poa* spp.	superficial	0.706405	0.000008
ACL-9635	Mitla	781208	1873324	1689	*Yucca filifera*	profundo	0.705913	0.000009
ACL-9636	Mitla	781289	1873332	1691	*Opuntia ficus*	superficial	0.705914	0.000008
ACL-9637	Mitla	781204	1873272	1695	*Pachycereus weberi*	superficial	0.706094	0.000006
ACL-9638	Mitla	781032	1873257	1686	*Poa* spp.	superficial	0.706143	0.000010
ACL-9095	Mitla Fortaleza	779009	1873969	1832	*Yucca filifera*	profundo	0.705918	0.000006
ACL-9096	Mitla Fortaleza	779009	1873969	1832	*Stenocereus thurberi*	superficial	0.705937	0.000006
ACL-9097	Mitla Fortaleza	779009	1873969	1832	*Agave* spp.	superficial	0.705900	0.000011
ACL-9098	Mitla Fortaleza	779009	1873969	1832	*Agave* spp.	superficial	0.705907	0.000014
ACL-9099	Mitla Fortaleza	779009	1873969	1832	*Agave* spp.	superficial	0.706536	0.000009
ACL-9100	Mitla Fortaleza	779009	1873969	1832	*Agave* spp.	superficial	0.705906	0.000007
ACL-9091	Monte Albán	737631	1885941	1940	*Agave* spp.	superficial	0.708005	0.000007
ACL-9092	Monte Albán	737631	1885941	1940	*Agave* spp.	superficial	0.707878	0.000007
ACL-9093	Monte Albán	737631	1885941	1940	*Leucaena leucocephala*	profundo	0.707691	0.000008
ACL-9094	Monte Albán	737631	1885941	1940	*Opuntia ficus*	superficial	0.707726	0.000014
ACL-10594	Monte Albán	737301	1886010	1881	*Agave* spp.	superficial	0.707935	0.000010
ACL-10595	Monte Albán	737449	1886012	1929	*Prosopis* spp.	profundo	0.707902	0.000007
ACL-10596	Monte Albán	737448	1886073	1929	*Agave* spp.	superficial	0.707607	0.000008
ACL-10597	Monte Albán	737478	1886104	1926	*Jacaranda mimosifolia*	superficial	0.707732	0.000006
ACL-10618	Ocotlán	755149	1856142	1475	*Pachycereus weberi*	superficial	0.705086	0.000009
ACL-10619	Ocotlán	750794	1856090	1475	*Agave* spp.	superficial	0.705144	0.000008
ACL-10620	Ocotlán	750558	1855995	1570	*Acacia farnesiana*	profundo	0.705119	0.000008
ACL-9611	San José Mogote	733521	1899560	1615	*Prosopis* spp.	profundo	0.706327	0.000008
ACL-9612	San José Mogote	733501	1899500	1616	*Opuntia ficus*	superficial	0.706104	0.000007
ACL-9613	San José Mogote	733643	1899366	1617	*Agave* spp.	superficial	0.706062	0.000006

ACL-9614	San José Mogote	733622	1899359	1606	*Prosopis* spp.	profundo	0.706574	0.000008
ACL-9615	San José Mogote	733802	1899534	1608	*Opuntia ficus*	superficial	0.706260	0.000012
ACL-9616	San José Mogote	733748	1899569	1612	*Prosopis* spp.	profundo	0.706270	0.000008
ACL-9083	San Martín Tilcajete	745202	1867422	1623	*Acacia farnesiana*	profundo	0.708259 [b]	0.000011
ACL-9084	San Martín Tilcajete	745202	1867422	1623	*Agave* spp.	superficial	0.709700 [b]	0.000013
ACL-9085	San Martín Tilcajete	745202	1867422	1623	*Opuntia ficus*	superficial	0.709633 [b]	0.000010
ACL-9086	San Martín Tilcajete	745202	1867422	1623	*Acacia farnesiana*	profundo	0.710458 [b]	0.000010
ACL-9087	San Martín Tilcajete	745202	1867422	1623	*Yucca filifera*	profundo	0.707351 [b]	0.000008
ACL-9088	San Martín Tilcajete	745202	1867422	1623	*Opuntia ficus*	superficial	0.707237 [b]	0.000006
ACL-9089	San Martín Tilcajete	745202	1867422	1623	*Acacia farnesiana*	profundo	0.708389 [b]	0.000009
ACL-9090	San Martín Tilcajete	745202	1867422	1623	*Dichondra argentea*	superficial	0.708531 [b]	0.000006
ACL-10604	San Martín Tilcajete	743657	1866063	1545	*Agave* spp.	superficial	0.708553 [b]	0.000010
ACL-10605	San Martín Tilcajete	743183	1866057	1579	*Ipomoea arborescens*	superficial	0.711976 [b]	0.000010
ACL-10606	San Martín Tilcajete	743005	1866148	1561	*Protium copal*	profundo	0.711739 [b]	0.000007
ACL-10607	San Martín Tilcajete	742561	1866081	1612	*Pachycereus weberi*	superficial	0.709570 [b]	0.000006
ACL-10614	Santa Catarina Minas	754393	1857425	1612	*Acacia farnesiana*	profundo	0.705971	0.000009
ACL-10615	Santa Catarina Minas	755036	1858109	1615	*Agave* spp.	superficial	0.705627	0.000008
ACL-10616	Santa Catarina Minas	755065	1858202	1631	*Opuntia ficus*	superficial	0.705870	0.000009

ACL-10617	Santa Catarina Minas	755120	1858541	1612	*Pachycereus weberi*	superficial	0.706535	0.000006
ACL-10621	Santa Rosa de Lima	745904	1856217	1527	*Agave* spp.	superficial	0.705435	0.000015
ACL-10623	Santa Rosa de Lima	745830	1854925	1533	*Jacaranda mimosifolia*	superficial	0.705533	0.000008
ACL-10624	Santa Rosa de Lima	747431	1854851	1527	*Pachycereus weberi*	superficial	0.705443	0.000009
ACL-10608	Santo Tomás Jalieza	748316	1865287	1609	*Agave* spp.	superficial	0.705024	0.000007
ACL-10609	Santo Tomás Jalieza	748255	1865409	1585	*Ipomoea arborescens*	superficial	0.704752	0.000006
ACL-10610	Santo Tomás Jalieza	748193	1865655	1585	*Pinus* spp.	profundo	0.704952	0.000007
ACL-10611	Santo Tomás Jalieza	748136	1867930	1615	*Protium copal*	profundo	0.706335	0.000009
ACL-10612	Santo Tomás Jalieza	748162	1865746	1597	*Acacia farnesiana*	profundo	0.704809	0.000011
ACL-10613	Santo Tomás Jalieza	748162	1865716	1588	*Agave* spp.	superficial	0.704783	0.000008
ACL-9617	Suchilquitongo	726283	1910455	1786	*Agave* spp.	superficial	0.706545	0.000008
ACL-9618	Suchilquitongo	726451	1910539	1804	*Prosopis* spp.	profundo	0.706795	0.000010
ACL-9619	Suchilquitongo	726293	1910688	1810	*Agave* spp.	superficial	0.706946	0.000015
ACL-9620	Suchilquitongo	726292	1910717	1808	*Opuntia ficus*	superficial	0.706938	0.000005
ACL-9621	Suchilquitongo	726289	1910739	1809	*Agave* spp.	superficial	0.707002	0.000013
ACL-9622	Suchilquitongo	726309	1910503	1815	*Agave* spp.	superficial	0.706519	0.000008
ACL-9646	Yagul	771545	1876618	1683	*Opuntia ficus*	superficial	0.705875	0.000011
ACL-9647	Yagul	771529	1876694	1690	*Opuntia ficus*	superficial	0.705920	0.000006
ACL-9648	Yagul	771582	1876791	1700	*Pachycereus weberi*	superficial	0.706043	0.000010
ACL-9649	Yagul	771649	1876788	1699	*Opuntia ficus*	superficial	0.705815	0.000010
ACL-9650	Yagul	771826	1876657	1727	*Opuntia ficus*	superficial	0.705874	0.000009
ACL-9651	Yagul	771800	1876708	1740	*Agave* spp.	superficial	0.705855	0.000011
ACL-9623	Zaachila	739726	1875665	1525	*Prosopis* spp.	profundo	0.708502	0.000005
ACL-9624	Zaachila	739719	1875686	1535	*Prosopis* spp.	profundo	0.708782	0.000009
ACL-9625	Zaachila	739735	1875711	1532	*Agave* spp.	superficial	0.708395	0.000007

ACL-9626	Zaachila	739722	1875763	1529	*Agave* spp.	superficial	0.707974	0.000008
ACL-9627	Zaachila	739718	1875801	1514	*Opuntia ficus*	superficial	0.708248	0.000014
ACL-9628	Zaachila	739699	1875702	1526	*Agave* spp.	superficial	0.708384	0.000008

[a]Todas las coordenadas UTM se encuentran en la zona UTM 14N.

[b]Identificamos estos valores de $^{87}Sr/^{86}Sr$ como inverosímilmente altos para la región y probablemente debidos al uso de fertilizantes agrícolas en la zona de recolección. Véase más información a continuación y en el Apéndice S1. Estas muestras se excluyeron del entrenamiento final del modelo.

Rendimiento del modelo isoscape

El modelo de estroncio continental, entrenado con datos compilados de América del Norte y del Sur, pero excluyendo Mesoamérica, logró un rendimiento predictivo moderado al aplicarse a los datos de prueba mesoamericanos (RMSE logit transformado = 0.42; R^2 bruto = 0.39). La incorporación de muestras mesoamericanas a los datos de entrenamiento mejoró sustancialmente el ajuste dentro de la muestra dentro de la región (RMSE logit = 0.18; RMSE bruto = 0.0011; R^2 bruto = 0.71), lo que destaca la importancia de la calibración regional.

Al probarse con las muestras independientes de Oaxaca no incluidas en el entrenamiento del modelo, el modelo continental mostró una capacidad explicativa limitada, pero significativa (RMSE logit = 0.34; RSME bruto = 0.0011; R^2 bruto = 0.33). La incorporación de datos mesoamericanos al entrenamiento mejoró la extrapolación a Oaxaca, reduciendo el error a 0.00087 en unidades brutas (RMSE logit = 0.26; R^2 bruto = 0.30).

En comparación, la línea base geológico de solo lecho rocoso tuvo un peor desempeño en ambas regiones, con RMSE bruto = 0.0019 y R^2 = 0.24 para Mesoamérica, y RMSE bruto = 0.0027 y R^2 = 0.37 para Oaxaca. Estos contrastes demuestran que los isopaisajes de aprendizaje automático, incluso cuando se entrenan a escala continental, proporcionan claras ganancias sobre las líneas base geológicas, y que la incorporación de muestras regionales mejora aún más la precisión predictiva sin sacrificar la capacidad de extrapolación (Tabla 3). En términos prácticos, la generalización fuera de la muestra a Oaxaca logró errores de menos de una parte en mil de la relación $^{87}Sr/^{86}Sr$, lo que subraya la utilidad de los modelos calibrados regionalmente. Sin embargo, para las predicciones espaciales posteriores, ajustamos el modelo final al conjunto de datos continental y mesoamericano completo (incluido Oaxaca) para maximizar la cobertura y la resolución.

Tabla 3. Métricas de rendimiento del modelo[a] en diferentes conjuntos de datos de entrenamiento y regiones de prueba.

Datos de entrenamiento	Datos de prueba	Logit RMSE	Logit R^2	RMSE bruto	R^2 bruto
**Fuera-de-muestra pruebas**					
Continental (sin Meso)	Mesoamérica[b]	0.42	0.60	0.0017	0.39
Continental (sin Meso)	Oaxaca	0.34	0.35	0.0011	0.33
Continental + Meso (sin Oaxaca)	Oaxaca	0.26	0.29	0.00087	0.30
Línea base de lecho rocoso[c]	Mesoamérica[b]	0.54	0.39	0.0019	0.24
Línea base de lecho rocoso[c]	Oaxaca	0.55	0.36	0.0027	0.37
**Dentro-de-muestra ajustes[d]**					

Continental + Meso (sin Oaxaca)	Mesoamérica[b]	0.18	0.89	0.0011	0.71
Continental + Meso + Oaxaca	Mesoamérica[b]	0.18	0.89	0.0011	0.71
Continental + Meso + Oaxaca	Oaxaca	0.22	0.5	0.0007	0.49

[a]Los valores muestran el RMSE (error cuadrático medio) y el R^2 (determinación del coeficiente) en unidades de $^{87}Sr/^{86}Sr$, tanto transformadas en logit como sin transformar (originales). Valores más bajos de RMSE y más altos de R^2 indican un mejor rendimiento del modelo.
[b]Mesoamérica se define como cualquier valor de $^{87}Sr/^{86}Sr$ de México, Belice, Guatemala, Honduras, El Salvador, Nicaragua y Costa Rica.
[c]El punto de referencia de lecho rocoso representa una línea base con información geológica [de 66] para la comparación con los modelos de aprendizaje automático.
[d]Indica medidas de rendimiento dentro de la muestra que podrían ser demasiado optimistas.

Variables predictivas

El análisis de importancia variable reveló varios factores clave que impulsan los patrones de distribución de isótopos de estroncio en la región. Los predictores más influyentes fueron la distancia desde la costa; la edad del basamento de la geología subyacente; el primer componente principal del modelo de lecho rocoso de estroncio; los componentes principales del clima relacionados con la intensidad y el momento de la precipitación; las variables de aerosoles relacionadas con la abundancia de carbono y sulfato y la concentración de polvo; y las variables del suelo relacionadas con la capacidad de intercambio catiónico y la textura (Fig. S1). Estos hallazgos se alinean con las expectativas teóricas para el ciclado del estroncio, donde la geología básica del lecho rocoso se filtra a través de los procesos de transporte del suelo, la meteorización climática y la deposición de aerosoles [67]. En particular, la distancia de alta resolución desde la costa fue favorecida sobre el mapa de aerosoles de sal marina de baja resolución en el modelo, lo que sugiere el claro valor agregado de los predictores de alta resolución, incluso si son indicadores indirectos de las variables impulsoras "verdaderas", en la estimación de las líneas de base locales de Sr.

Distribución espacial de los valores predichos

El modelo calibrado final produjo estimaciones de valores de $^{87}Sr/^{86}Sr$ en Mesoamérica, que oscilaron entre 0.704 y 0.712 (Fig. 3; los intervalos de confianza del 95% superior e inferior de las predicciones de rangos de $^{87}Sr/^{86}Sr$ están disponibles en la Fig. S2). El modelo reveló varias regiones isotópicas distintas. Se identificaron áreas con valores elevados de $^{87}Sr/^{86}Sr$ (>0.709) en la Sierra Maya, la Península de Yucatán y las cordilleras costeras de Oaxaca, lo cual es consistente con formaciones geológicas más antiguas en estas regiones y con datos publicados disponibles de $^{87}Sr/^{86}Sr$ [104]. Se observaron áreas menos radiogénicas (<0.706) en la Sierra de Tuxtla en Veracruz y a lo largo de una franja del eje volcánico central, lo que refleja una geología volcánica más reciente. Curiosamente, el modelo ajustado que incorpora muestras de plantas de Oaxaca redujo las proporciones isotópicas estimadas de estroncio en la mayor parte de México, aumentando solo ligeramente las estimaciones en las regiones costeras del sur de

Oaxaca y Guerrero. En general, los valores y rangos predichos por isopaisajes se ajustan generalmente a los datos resumidos regionales publicados de $^{87}Sr/^{86}Sr$ en Mesoamérica [54], a la vez que ofrecen una visión más clara de la variabilidad espacial regional del estroncio.

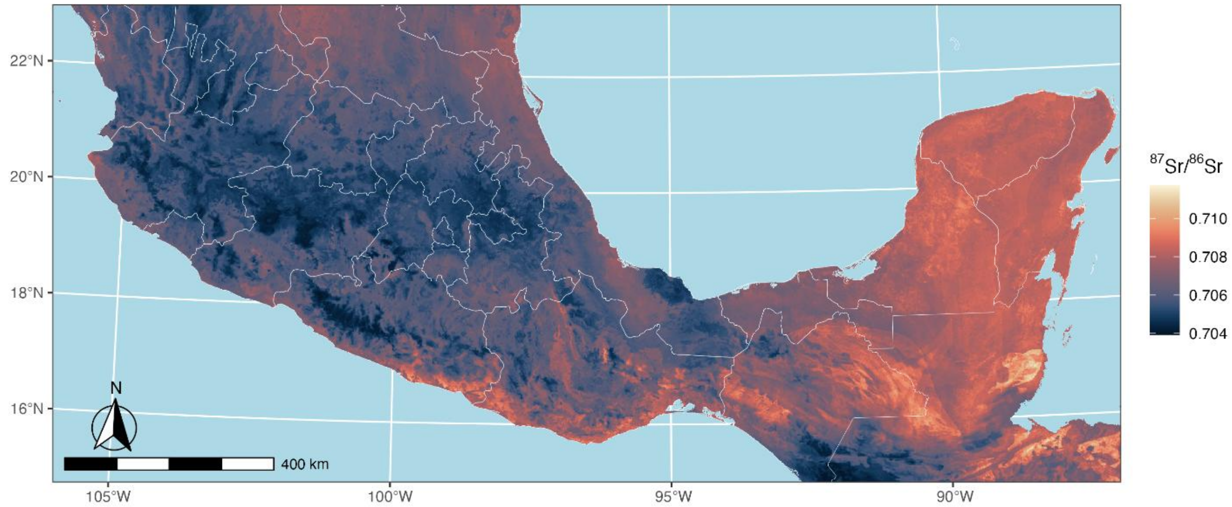

Fig. 3. Modelo isoscape calibrado final de la variación de $^{87}Sr/^{86}Sr$ en Mesoamérica.

En el Valle de Oaxaca, nuestro isopaisaje sugiere que deberíamos ser capaces de detectar la movilidad intravalle sugerida por los datos de prospección de asentamientos arqueológicos (Fig. 4). Aunque cada brazo del Valle exhibe cierta superposición en los valores predichos de $^{87}Sr/^{86}Sr$, el brazo oriental de Tlacolula del Valle está dominado por grandes áreas con valores predichos más bajos de $^{87}Sr/^{86}Sr$ (~0.705-0.706) que concuerdan con las expectativas de su geología subyacente del Mesozoico y Cenozoico más reciente. En contraste, el brazo noroccidental de Etla tiene valores predichos más altos de $^{87}Sr/^{86}Sr$ (~0.706-0.707), y el brazo sur de Ocotlán-Zimatlán presenta algunos de los valores predichos más altos de $^{87}Sr/^{86}Sr$ dentro del Valle (~0.7075-0.708). Estos valores más altos en los brazos de Etla y Octolán-Zimatlán coinciden con su proximidad al Complejo Precámbrico Oaxaqueño. Por lo tanto, si bien el isopaisaje ciertamente permite la identificación de individuos no locales dentro del Valle de Oaxaca que pueden haberse originado en otras partes del Valle, la falta de valores $^{87}Sr/^{86}Sr$ mutuamente excluyentes entre los brazos limita nuestra capacidad de geolocalizar exactamente de dónde se originaron estos no locales.

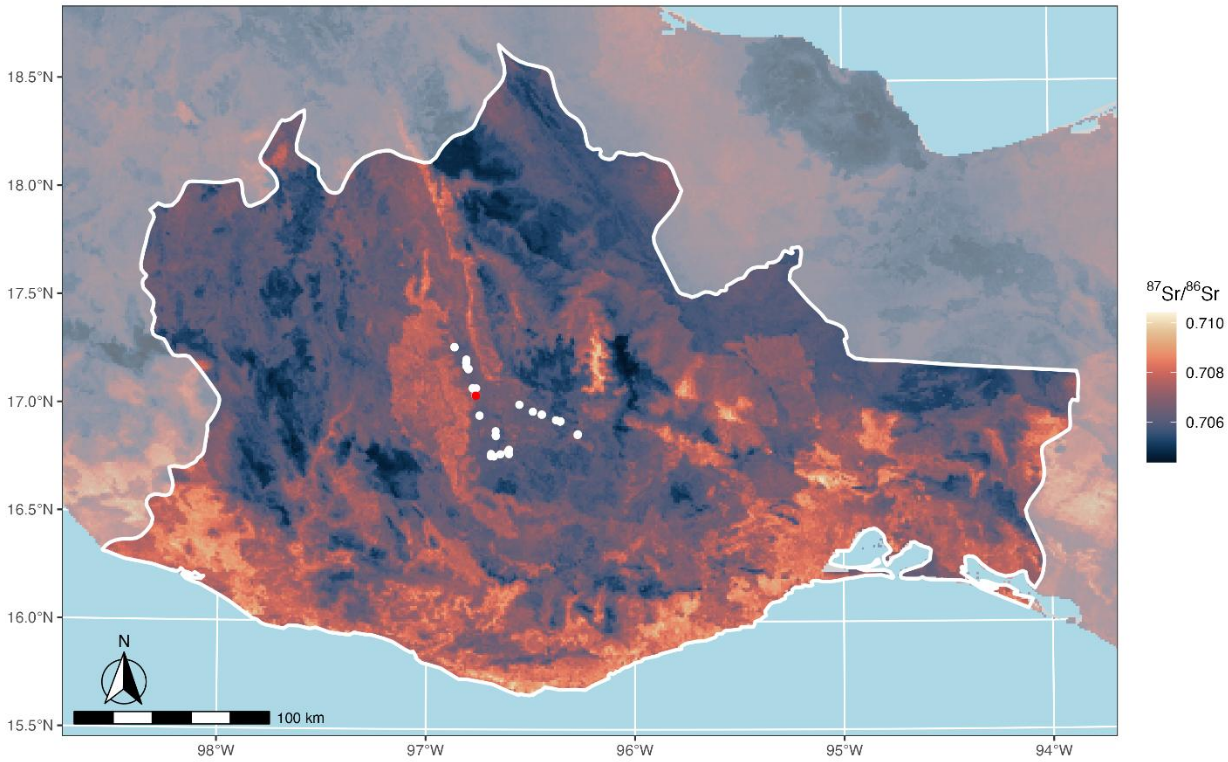

Fig. 4. Modelo final calibrado de isopaisaje $^{87}Sr/^{86}Sr$ en el actual estado de Oaxaca. Los sitios donde se recolectaron muestras de plantas para entrenar el modelo BART se muestran en blanco, con la excepción de Monte Albán, que se muestra en rojo.

Patrones de incertidumbre

El enfoque de modelado BART proporcionó estimaciones nativas de la incertidumbre para todas las predicciones en el isopaisaje (Fig. 5). En consonancia con estudios previos, observamos que la incertidumbre en la predicción del estroncio aumentaba con la proporción de estroncio, y que las proporciones más altas y radiogénicas se asociaban con una mayor incertidumbre debido a la mayor variabilidad en los procesos de meteorización y transporte en regiones con lechos rocosos más antiguos y radiogénicos. Los lechos rocosos más antiguos presentan una mayor variación en las proporciones $^{87}Sr/^{86}Sr$ entre minerales coexistentes, que presentan una susceptibilidad diferente a la meteorización. La coincidencia entre las predicciones del modelo y las observaciones de estroncio fue, en general, buena, sin una estructura espacial clara en los residuos que indicara procesos significativos no modelados. Sin embargo, observamos desajustes persistentes entre las proporciones isotópicas modeladas y observadas en contextos específicos. Estas discrepancias se produjeron principalmente en contextos de enterramientos humanos cerca de las principales transiciones de lecho rocoso, donde cabría esperar una variabilidad relativamente alta en las proporciones de estroncio dentro de la cuenca local. Entre los ejemplos se incluyen individuos del embalse Chalillo, en el río Macal, al borde de las Montañas Mayas, isotópicamente distintas, así como individuos de centros regionales como Cholula, un lugar de peregrinación en el centro de México, y Tikal, una ciudad-estado maya dominante del período Clásico. Durante nuestro análisis, también detectamos un grupo de muestras de nuestras plantas de Oaxaca en San Martín Tilcajete con proporciones de estroncio inverosímilmente altas para la

región (Tabla 2). Investigaciones posteriores revelaron que estas muestras probablemente reflejan contaminación por la aplicación de fertilizantes agrícolas modernos (Apéndice S1) y se excluyeron del entrenamiento final del modelo.

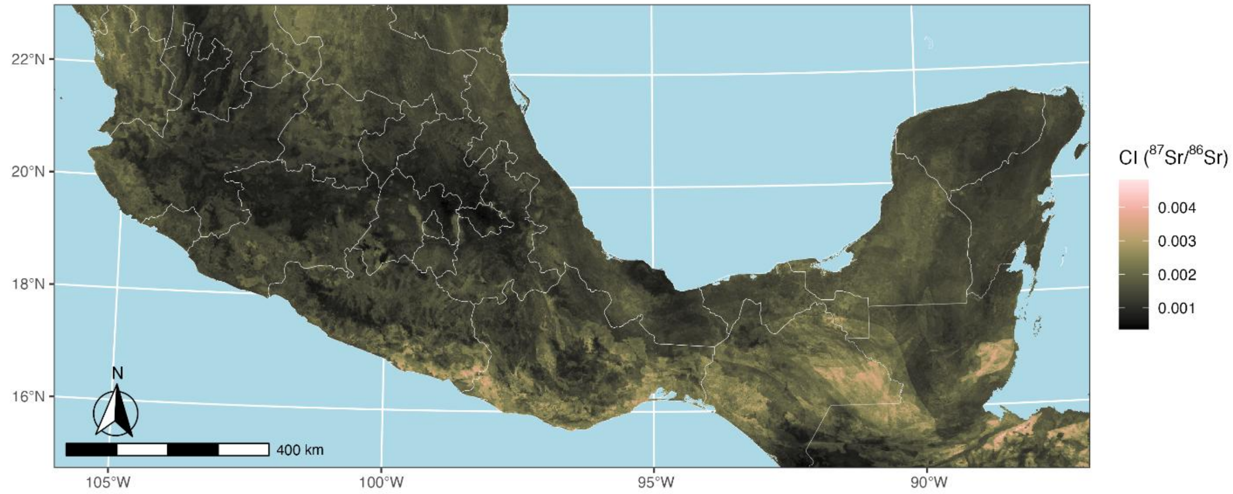

Fig. 5. Estimaciones de incertidumbre nativa para todos los valores predichos de $^{87}Sr/^{86}Sr$ en el isopaisaje.

Discusión

Nuestros resultados indican distinciones isotópicas regionales mucho menos claras que las que podría sugerir el lecho rocoso subyacente por sí solo, lo que refleja la influencia variable de los procesos climáticos y edáficos en la biodisponibilidad de $^{87}Sr/^{86}Sr$. La incertidumbre en las predicciones, si bien reducida con respecto al modelo global, sigue siendo relativamente alta en comparación con el rango de la mayoría de los valores. Por lo tanto, las evaluaciones de la migración basadas en isótopos de estroncio podrían ser más variables de lo esperado a partir de la geología del lecho rocoso únicamente.

Uso del isopaisaje predictivo de $^{87}Sr/^{86}Sr$

Para demostrar la utilidad de nuestro modelo predictivo de isopaisaje de $^{87}Sr/^{86}Sr$, lo aplicamos a valores humanos publicados de $^{87}Sr/^{86}Sr$ (n=10) de Monte Albán [10]. Las observaciones comprenden muestras pareadas de hueso y esmalte de $^{87}Sr/^{86}Sr$ de cinco individuos que datan de la fase Monte Albán II (100 a.C. - 200 d.C.) y la fase Monte Albán III (200-500 d.C.). Price y sus colegas [10] identifican los valores de $^{87}Sr/^{86}Sr$ en hueso y diente de un individuo como significativamente inferiores a los de los otros cuatro individuos de Monte Albán. Los autores sugieren que este individuo probablemente vivió fuera de Monte Albán durante su primera infancia (esmalte) y en los últimos ~10 años de su vida (hueso). Sin embargo, esta evaluación no se basa en una evaluación de los datos de referencia biogeoquímicos de $^{87}Sr/^{86}Sr$, ya que no existen datos ambientales de $^{87}Sr/^{86}Sr$ publicados previamente para Monte Albán.

La cuantificación explícita de la incertidumbre de nuestro modelo permite una interpretación más matizada de las muestras arqueológicas. Por ejemplo, al evaluar individuos de Monte Albán, nuestro modelo proporciona no solo una estimación puntual de la señal de estroncio "local"

basada en la celda de 1 km del yacimiento dentro del isopaisaje generado, sino también una distribución de probabilidad que considera la incertidumbre de la predicción (Fig. 5). Este enfoque bayesiano ofrece un marco más robusto para identificar migrantes potenciales a lo largo de un espectro continuo de localidad, a diferencia de los métodos tradicionales basados en umbrales que construyen una binaria rígida de local/no local. Dado que BART funciona mediante el desarrollo de un conjunto de múltiples árboles de decisión, cada predicción representa una matriz de valores predichos, uno de cada árbol del conjunto. Esta distribución de valores predichos facilita el cálculo de estadísticas de resumen a posteriori, como la media, la mediana o los cuantiles superior e inferior del valor predicho en cualquier dado píxel.

El isopaisaje predice que los valores locales de $^{87}Sr/^{86}Sr$ en Monte Albán oscilan entre 0.7069 y 0.7086, con una mediana de $^{87}Sr/^{86}Sr$ =0.7077. Este rango representa el rango intercuartil de la distribución predictiva posterior del modelo BART final para los valores de $^{87}Sr/^{86}Sr$ en Monte Albán (Fig. 6) y es algo más amplio que una línea base biogeoquímica tradicional basada en umbrales, generada a partir de los valores ambientales de $^{87}Sr/^{86}Sr$. Por ejemplo, nuestros valores observados de $^{87}Sr/^{86}Sr$ en la planta de Monte Albán oscilaron entre 0.707607 y 0.708005, con una media de $^{87}Sr/^{86}Sr$ =0.707809 ± 0.000139 (1σ, n=8). Aunque más amplio, el modelo BART produce un rango local calibrado según las fuentes geológicas y ambientales reales de estroncio, que captura con mayor precisión el ciclo de $^{87}Sr/^{86}Sr$ en el entorno, en lugar de umbrales estadísticos impuestos arbitrariamente, independientes del contexto ambiental más amplio. Además, el rango local predicho por nuestro modelo de isopaisaje ofrece ventajas adicionales sobre las líneas de base biogeoquímicas estadísticas tradicionales generadas a partir de los valores ambientales observados de $^{87}Sr/^{86}Sr$. Mientras que recolectamos muestras de plantas de forma oportunista (y, por lo tanto, idiosincrásica) en una porción limitada del sitio, el isopaisaje proporciona un rango local estimado de $^{87}Sr/^{86}Sr$ geográficamente estandarizado para Monte Albán en un radio de 1 km, lo que nos permite caracterizar con mayor precisión los valores locales de estroncio.

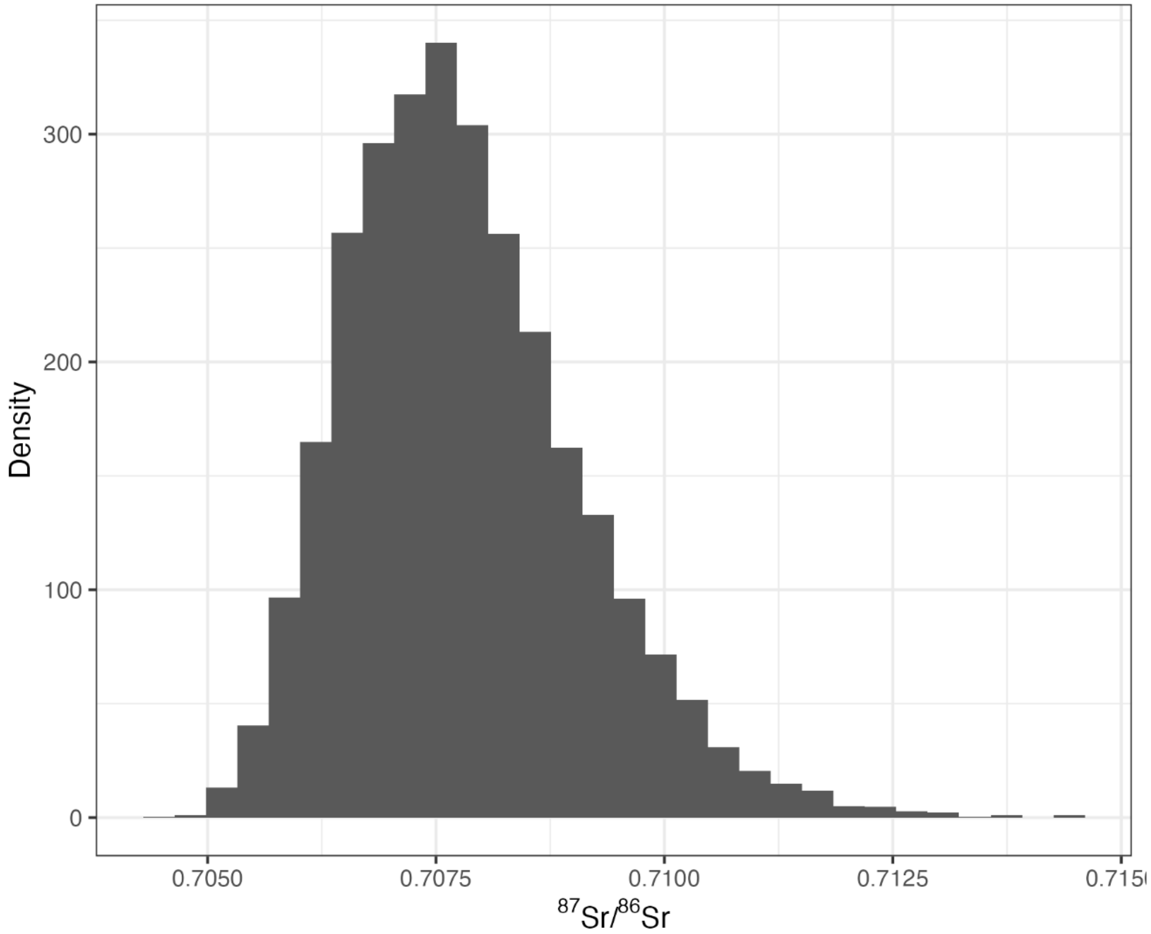

Fig. 6. Distribución predictiva posterior del $^{87}Sr/^{86}Sr$ "local" esperado en Monte Albán a partir del modelo BART final.

Aun así, el rango de $^{87}Sr/^{86}Sr$ previsto para el isopaisaje representa solo una aproximación conservadora de los valores locales de $^{87}Sr/^{86}Sr$ en Monte Albán. Nuestro rango local previsto se basa en una sola celda de 1 km del modelo del isopaisaje. Sin embargo, un estudio arqueológico de Monte Albán muestra que el sitio tenía una superficie aproximada de 3 km² [14]. Si bien Nicholas y Feinman proponen de 1 a 2 km como una zona de captación razonable para la subsistencia agrícola en la mayoría de los asentamientos prehispánicos del Valle de Oaxaca, concluyen que Monte Albán probablemente extrajo agua de un radio superior a los 2 km para satisfacer sus necesidades de subsistencia [23]. Si bien no podemos saber exactamente de dónde Monte Albán obtenía su suministro de alimentos (y, por lo tanto, la ingesta de $^{87}Sr/^{86}Sr$ de sus residentes), el rango isopaisaje previsto, más amplio pero imperfecto, de 1 km ciertamente representa una caracterización más realista de los valores locales esperados de $^{87}Sr/^{86}Sr$ en los antiguos residentes de Monte Albán que el rango local más estrecho basado únicamente en muestras de plantas modernas.

Al aplicar el rango local predicho por el análisis de isótopos a los valores de $^{87}Sr/^{86}Sr$ humanos de Monte Albán publicados previamente y sus errores estándar asociados, obtenemos una comprensión más precisa de la paleomovilidad en el yacimiento (Fig. 7). Si bien los individuos

previamente clasificados como locales (n=4) y no locales (n=1) permanecen cómodamente en estas categorías [10], la ubicación de sus tejidos muestreados, ya sea dentro o en los límites del rango local predicho por Monte Albán, nos permite inferir la certeza o incertidumbre relativa de estas designaciones. Por ejemplo, si el error estándar asociado a un valor de $^{87}Sr/^{86}Sr$ de esmalte o hueso "local" se extendiera más allá del rango local predicho por Monte Albán, esto indicaría una mayor incertidumbre en la designación de residencia de ese individuo. Sin embargo, este no es el caso entre los individuos de Monte Albán, donde todos los valores de $^{87}Sr/^{86}Sr$ publicados previamente presentaron errores estándar asociados extremadamente bajos (<0.00002). Estos márgenes de error eran menores que el tamaño de píxel de los puntos y, por lo tanto, no se visualizaron en la figura. Esto indica que a cada uno de estos tejidos se le puede asignar con seguridad un estatus residencial "local" o "no local."

Además, casi todos los tejidos "locales" (es decir, los tejidos muestreados cuyos valores observados de $^{87}Sr/^{86}Sr$ se encuentran dentro del rango local predicho por Monte Albán) presentan valores de $^{87}Sr/^{86}Sr$ que se agrupan estrechamente en torno al valor mediano predicho por Monte Albán para un tejido "local" de $^{87}Sr/^{86}Sr = 0.7077$. La medición de $^{87}Sr/^{86}Sr$ del fémur del entierro 26A representa la única excepción entre los tejidos "locales," situándose cerca del límite inferior del rango local predicho por Monte Albán. Esto sigue siendo consistente con un individuo que vivió en la zona durante los últimos ~10 años de su vida. Sin embargo, valores de $^{87}Sr/^{86}Sr$ óseos cercanos a los límites del rango local previsto podrían indicar que este individuo se trasladó a Monte Albán recientemente, en su vejez, y que sus valores de $^{87}Sr/^{86}Sr$ óseos se aclimataron a los valores locales de $^{87}Sr/^{86}Sr$ al momento de su muerte. De este modo, obtenemos una mayor certeza sobre la clasificación de los individuos enterrados en Monte Albán como "locales" o "no locales", reconociendo que la localidad biogeoquímica (es decir, estar dentro del rango local previsto para Monte Albán) no equivale necesariamente a la localidad cultural.

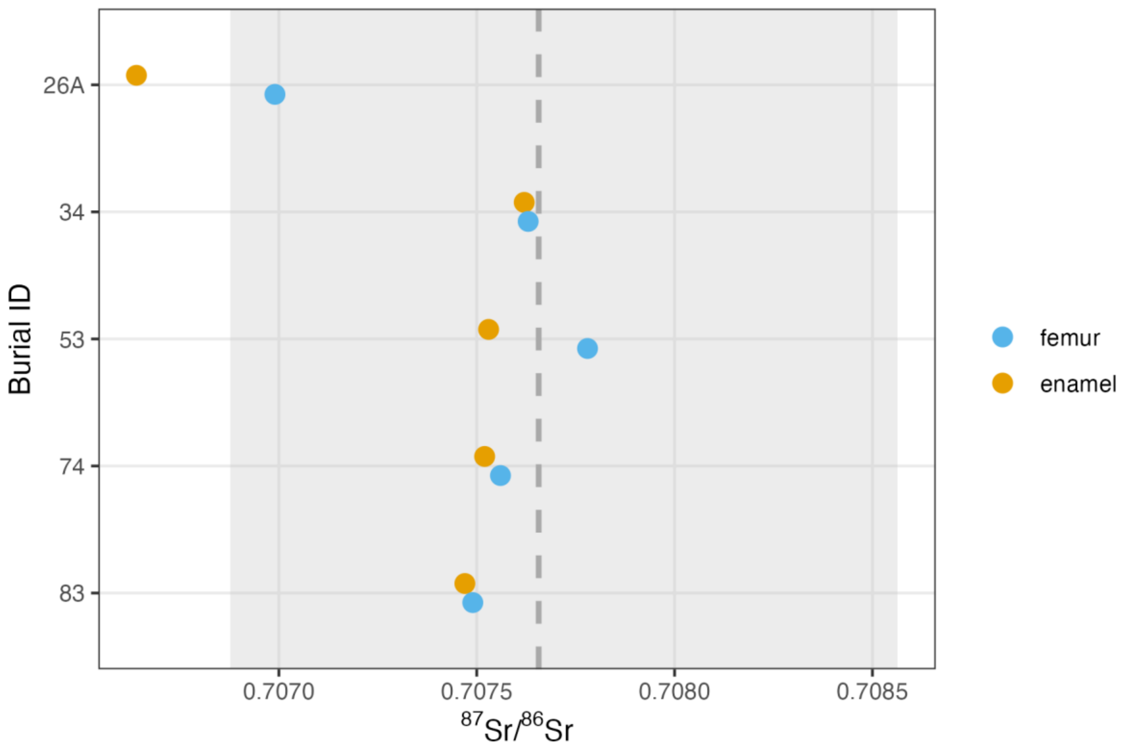

Figura 7. Comparación de muestras pareadas de esmalte y hueso humanos de Monte Albán, publicadas previamente, con las predicciones de BART en Monte Albán. La línea discontinua gris indica la mediana de la predicción de BART para la relación $^{87}Sr/^{86}Sr$ en Monte Albán. El área sombreada gris indica el rango intercuartílico de las predicciones de BART para los valores locales de $^{87}Sr/^{86}Sr$ en Monte Albán. El error estándar reportado para cada valor observado de $^{87}Sr/^{86}Sr$ fue suficientemente bajo (<0.00002) como para que no se incluyeran barras de error en la gráfica [10].

Conclusión

Creamos un modelo BART predictivo de isopaisajes de $^{87}Sr/^{86}Sr$ para Mesoamérica y el Valle de Oaxaca, integrando datos empíricos novedosos y compilados de $^{87}Sr/^{86}Sr$ mesoamericanos, además de mapas geológicos y datos espaciales de covariantes geoambientales de Sr. El mejor desempeño del modelo BART en comparación con un modelo global de isopaisajes de $^{87}Sr/^{86}Sr$ basado únicamente en el lecho rocoso geológico indica tanto la importancia de la calibración regional en el desarrollo de isopaisajes predictivos como la importancia de considerar la influencia variable de factores geoambientales, como el clima y los procesos edáficos, en la biodisponibilidad de $^{87}Sr/^{86}Sr$. Prevemos que el modelo se mejorará aún más con la incorporación de valores empíricos adicionales de $^{87}Sr/^{86}Sr$ de toda Mesoamérica, particularmente en regiones con estimaciones de incertidumbre nativa elevadas.

Nuestro isopaisaje indica una clara variación de $^{87}Sr/^{86}Sr$ dentro de la gran Mesoamérica y el Valle de Oaxaca. Si bien existe cierta superposición en los valores de $^{87}Sr/^{86}Sr$ en las tres ramas del Valle de Oaxaca, existe suficiente variación entre ellas para detectar la migración intravalle, así como la migración entre el Valle y la Gran Mesoamérica. Al proporcionar un isopaisaje predictivo de $^{87}Sr/^{86}Sr$ de alto rendimiento para el Valle, esperamos que las investigaciones

biogeoquímicas de la migración en la región se expandan más allá de su enfoque inicial en Monte Albán, capital del estado zapoteca. La inclusión de las tres ramas del Valle en el isopaisaje permitirá a los investigadores considerar preguntas sobre la escala de la migración en el Valle y el papel que la migración intrarregional e interregional desempeñó en la formación y el mantenimiento del estado zapoteca, así como en el desarrollo cultural posterior de la región.

Finalmente, el isopaisaje proporciona a los arqueólogos una herramienta más refinada para el examen y análisis de los patrones migratorios pasados en la antigua Mesoamérica. Las predicciones estandarizadas y geoambientales del modelo BART sobre los rangos "locales" esperados de $^{87}Sr/^{86}Sr$ para sitios arqueológicos de interés ofrecen claras ventajas sobre el uso de enfoques estadísticos tradicionales basados en umbrales, que no consideran los efectos del contexto ambiental más amplio sobre la variabilidad local de $^{87}Sr/^{86}Sr$. Además, la cuantificación explícita de la incertidumbre en los valores predichos de $^{87}Sr/^{86}Sr$ proporciona a los arqueólogos información crucial sobre la confianza en los rangos "locales" esperados de $^{87}Sr/^{86}Sr$, lo cual impacta directamente en la fiabilidad de la identificación de migrantes.

Agradecimientos

Le agradecemos a Pedro Fabián Ojeda por su ayuda en la recolección de muestras de plantas en el ramal Ocotlán-Zimatlán del Valle de Oaxaca. En el Laboratorio de Arqueológica Química de la Universidad Estatal de Arizona, les agradecemos a los asistentes de investigación Eric Flores, Kari Guilbault, Sparshee Naik y Tajinder Virdee por su ayuda con el procesamiento de las muestras. En el Laboratorio de Análisis de Metales, Ambiental y Terrestre, agradecemos la ayuda del Dr. Stephen Romaniello, el Dr. Trevor Martin y Natasha Zolotova.

Referencias

1. Blanton RE, Kowalewski SA, Feinman GM, Appel J. Monte Alban's hinterland, part I: the prehispanic settlement patterns of the central and Southern parts of the Valley of Oaxaca, Mexico. Ann Arbor, MI: Museum of Anthropology, University of Michigan; 1982.

2. Caso A. Las tumbas de Monte Albán. Anales del Instituto Nacional de Antropología e Historia. 1933; 641–648.

3. Feinman GM, Nicholas LM. Settlement patterns of the Ejutla Valley, Oaxaca, Mexico: a diachronic macroscale perspective. Fieldiana Anthropology. 2013; 1–330.

4. Romero J. El material osteológico de Monte Albán, Mexico. Boletín Bibliográfico de Antropología Americana. 1949;12: 166–168.

5. Saville MH. Exploration of Zapotecan tombs in southern Mexico. American Anthropologist. 1899;1: 350–362. doi:10.1525/aa.1899.1.2.02a00080

6. Feinman GM, Blanton RE, Nicholas LM. The emergence of Monte Albán: a social innovation that lasted a millennium. 1st ed. In: Thurston TL, Fernandez-Gotz M, editors. Power from Below in Premodern Societies. 1st ed. Cambridge University Press; 2021. pp. 220–246. doi:10.1017/9781009042826.011

7. Spence MW, White CD. Mesoamerican bioarchaeology: past and future. Ancient Mesoamerica. 2010;20: 233–240. doi:10.1017/S0956536109990083

8. Ebert CE, Hixon SW, Buckley GM, George RJ, Pacheco-Forés SI, Palomo JM, et al. The Caribbean and Mesoamerica Biogeochemical Isotope Overview (CAMBIO). Sci Data. 2024;11: 349. doi:10.1038/s41597-024-03167-6

9. Casar I, Márquez L, Cienfuegos E, González Licón E, Somerville AD. Monte Alban and Teotihuacan connections: can stable isotope analysis of bone and enamel detect migration between two ancient Mesoamerican urban capitals? Archaeol Anthropol Sci. 2022;14: 220. doi:10.1007/s12520-022-01683-3

10. Price TD, Manzanilla LR, Middleton WD. Immigration and the ancient city of Teotihuacan in Mexico: a study using strontium isotope ratios in human bone and teeth. Journal of Archaeological Science. 2000;27: 903–913. doi:10.1006/jasc.1999.0504

11. Warinner C, García NR, Spores R, Tuross N. Disease, demography, and diet in early colonial New Spain: investigation of a sixteenth-century Mixtec cemetery at Teposcolula Yucundaa. Latin American Antiquity. 2012;23: 467–489. Available: http://www.ingentaconnect.com/content/saa/laa/2012/00000023/00000004/art00006

12. White CD, Spence MW, Stuart-Williams HLQ, Schwarcz HP. Oxygen isotopes and the identification of geographical origins: the Valley of Oaxaca versus the Valley of Mexico. Journal of Archaeological Science. 1998;25: 643–655. doi:10.1006/jasc.1997.0259

13. Bowen GJ, West JB. Isotope landscapes for terrestrial migration research. Terrestrial Ecology. Elsevier; 2008. pp. 79–105. doi:10.1016/S1936-7961(07)00004-8

14. Blanton RE. Monte Albán: settlement patterns at the ancient Zapotec capital. New York, NY: Academic Press; 1978.

15. Redmond EM, Spencer CS. From raiding to conquest: warfare strategies and early state development in Oaxaca, Mexico. In: Arkush EN, Allen MW, editors. The archaeology of warfare: prehistory of raiding and conquest. Gainesville, FL: University Press of Florida; 2006. pp. 336–393.

16. Spencer CS, Redmond EM. Militarism, resistance, and early state development in Oaxaca, Mexico. Social Evolution & History. 2003;2: 25–70. Available: https://cyberleninka.ru/article/n/militarism-resistance-and-early-state-development-in-oaxaca-mexico

17. Carpenter LB. Households and Political Transformation: Daily Life During State Formation at Tilcajete, Oaxaca, Mexico. Ph.D. dissertation, University of Michigan. 2019.

18. Blanton RE, Finsten L, Kowalewski SA, Feinman GM. Migration and population change in the prehispanic Valley of Oaxaca, Mexico. In: Mastache AG, Parsons JR, Santley RS, Serra Puche MC, editors. Arqueología mesoamericana: homenaje a William T Sanders. Mexico City: Instituto Nacional de Antropología e Historia; 1996. pp. 11–36.

19. Feinman GM, Kowalewski SA, Finsten L, Blanton RE, Nicholas LM. Long-term demographic change: a perspective from the Valley of Oaxaca. Journal of Field Archaeology. 1985;12: 333–362. doi:10.1179/jfa.1985.12.3.333

20. Marcus J, Flannery KV. Zapotec civilization: how urban society evolved in Mexico's Oaxaca Valley. London: Thames & Hudson; 1996.

21. Kowalewski SA, Feinman GM, Finsten L, Blanton RE, Nicholas LM. Monte Albán's hinterland, part II. Ann Arbor, MI: Museum of Anthropology, University of Michigan; 1989.

22. Marcus J. Woman's ritual in Formative Oaxaca: figurine making, divination, death and the ancestors. Ann Arbor, MI: University of Michigan Museum of Anthropology; 1998.

23. Nicholas LM, Feinman GM. The foundation of Monte Albán, intensification, and growth: coactive processes and joint production. Front Polit Sci. 2022;4: 805047. doi:10.3389/fpos.2022.805047

24. Spencer CS, Redmond EM. Primary state formation in Mesoamerica. Annu Rev Anthropol. 2004;33: 173–199. doi:10.1146/annurev.anthro.33.070203.143823

25. Elson CM. Excavations at Cerro Tilcajete: a Monte Albán II administrative center in the Valley of Oaxaca. Ann Arbor, MI: Museum of Anthropology, University of Michigan; 2007.

26. Márquez Morfín L, Negrete-Gutiérrez SS, Pérez-Flórez AM. A Teotihuacano in Monte Alban: life history in a domestic group from an osteobiographic approach. 1st ed. In: Wrobel GD, Cucina A, editors. Mesoamerican Osteobiographies. 1st ed. Gainesville, FL: University Press of Florida; 2024. pp. 138–150. doi:10.2307/jj.16430753.13

27. Spence MW. A Zapotec diaspora network in Classic-period central Mexico. In: Stein GJ, editor. The archaeology of colonial encounters: comparative perspectives. Santa Fe, NM: School of American Research Press; 2005. pp. 173–206.

28. Ehleringer JR, Bowen GJ, Chesson LA, West AG, Podlesak DW, Cerling TE. Hydrogen and oxygen isotope ratios in human hair are related to geography. Proceedings of the National Academy of Sciences. 2008;105: 2788–2793. Available: http://www.pnas.org/content/105/8/2788.short

29. Ericson JE. Strontium isotope characterization in the study of prehistoric human ecology. Journal of Human Evolution. 1985;14: 503–514. Available: http://www.sciencedirect.com/science/article/pii/S0047248485800294

30. Gulson BL, Jameson WW, Gillings BR. Stable lead isotopes in teeth as indicators of past domicile-a potential new tool in forensic science? Journal of forensic sciences. 1997;42: 787–791. Available: http://www.researchgate.net/profile/Brian_Gulson/publication/13916426_Stable_lead_isoto

pes_in_teeth_as_indicators_of_past_domicile--a_potential_new_tool_in_forensic_science/links/00b4952a7e1534f912000000.pdf

31. Richards MP, Fuller BT, Hedges REM. Sulphur isotopic variation in ancient bone collagen from Europe: implications for human palaeodiet, residence mobility, and modern pollutant studies. Earth and Planetary Science Letters. 2001;191: 185–190. doi:10.1016/S0012-821X(01)00427-7

32. Schwarcz HP, Gibbs L, Knyf M. Oxygen isotope analysis as an indicator of place of origin. Snake Hill: an investigation of a military cemetery from the War of 1812. Toronto, ON: Dundurn Press; 1991. pp. 263–268.

33. Bowen GJ, Wassenaar LI, Hobson KA. Global application of stable hydrogen and oxygen isotopes to wildlife forensics. Oecologia. 2005;143: 337–348. Available: http://www.jstor.org.ezproxy1.lib.asu.edu/stable/20062256

34. Dansgaard W. Stable isotopes in precipitation. Tellus. 1964;16: 436–468. doi:10.1111/j.2153-3490.1964.tb00181.x

35. Gat JR. Oxygen and hydrogen isotopes in the hydrologic cycle. Annual Review of Earth and Planetary Sciences. 1996;24: 225–262. doi:10.1146/annurev.earth.24.1.225

36. Luz B, Kolodny Y. Oxygen isotope variation in bone phosphate. Applied Geochemistry. 1989;4: 317–323. doi:10.1016/0883-2927(89)90035-8

37. Bentley RA. Strontium isotopes from the earth to the archaeological skeleton: a review. J Archaeol Method Theory. 2006;13: 135–187. doi:10.1007/s10816-006-9009-x

38. Faure G, Powell JL. Strontium isotope geology. New York, NY: Springer-Verlag; 1972.

39. Krouse H, Levinson A. Geographical trends of carbon and sulphur isotope abundances in human kidney stones. Geochimica et Cosmochimica Acta. 1984;48: 187–191. doi:10.1016/0016-7037 (84)90360-0

40. Nehlich O. The application of sulphur isotope analyses in archaeological research: a review. Earth-Science Reviews. 2015;142: 1–17. doi:10.1016/j.earscirev.2014.12.002

41. Stille P, Shields G. Radiogenic isotope geochemistry of sedimentary and aquatic systems. Berlin: Springer; 1997. doi:10.1007/BFb0117747

42. Flockhart DTT, Kyser TK, Chipley D, Miller NG, Norris DR. Experimental evidence shows no fractionation of strontium isotopes (87Sr/86Sr) among soil, plants, and herbivores: implications for tracking wildlife and forensic science. Isotopes in Environmental and Health Studies. 2015;51: 372–381. doi:10.1080/10256016.2015.1021345

43. Kamenov GD, Gulson BL. The Pb isotopic record of historical to modern human lead exposure. Science of The Total Environment. 2014;490: 861–870. doi:10.1016/j.scitotenv.2014.05.085

44. Longinelli A. Oxygen isotopes in mammal bone phosphate: a new tool for paleohydrological and paleoclimatological research? Geochimica et Cosmochimica Acta. 1984;48: 385–390. doi:10.1016/0016-7037(84)90259-X

45. Luz B, Kolodny Y, Horowitz M. Fractionation of oxygen isotopes between mammalian bone-phosphate and environmental drinking water. Geochimica et Cosmochimica Acta. 1984;48: 1689–1693. doi:10.1016/0016-7037(84)90338-7

46. Richards MP, Fuller BT, Sponheimer M, Robinson T, Ayliffe L. Sulphur isotopes in palaeodietary studies: a review and results from a controlled feeding experiment. International Journal of Osteoarchaeology. 2003;13: 37–45. doi:10.1002/oa.654

47. Sealy JC, van der Merwe NJ, Sillen A, Kruger FJ, Krueger HW. 87Sr/86Sr as a dietary indicator in modern and archaeological bone. Journal of Archaeological Science. 1991;18: 399–416. doi:10.1016/0305-4403(91)90074-Y

48. Turekian KK, Kulp JL. Strontium content of human bones. Science. 1956;124: 405–407. Available: http://www.jstor.org/stable/1751986

49. Knudson KJ, Stanish C, Lozada Cerna MC, Faull KF, Tantaleán H. Intra-individual variability and strontium isotope measurements: A methodological study using 87Sr/86Sr data from Pampa de los Gentiles, Chincha Valley, Peru. Journal of Archaeological Science: Reports. 2016;5: 590–597. doi:10.1016/j.jasrep.2016.01.016

50. Olivares Flores E. Aspectos sobre dieta y procedencia de los cráneos esgrafiados de la Casa del Mendrugo, a partir de análisis de isótopos estables. M.A. thesis, Universidad Nacional Autónoma de México. 2021. Available: http://132.248.9.195/ptd2021/febrero/0809591/0809591.pdf

51. Price TD, Burton JH, Bentley RA. The characterization of biologically available strontium isotope ratios for the study of prehistoric migration. Archaeometry. 2002;44: 117–135. doi:10.1111/1475-4754.00047

52. Ezzo JA, Johnson CM, Price TD. Analytical perspectives on prehistoric migration: a case study from east-central Arizona. Journal of Archaeological Science. 1997;24: 447–466. doi:10.1006/jasc.1996.0129

53. Price TD, Johnson CM, Ezzo JA, Ericson J, Burton JH. Residential mobility in the prehistoric southwest United States: a preliminary study using strontium isotope analysis. Journal of Archaeological Science. 1994;21: 315–330. Available: http://www.sciencedirect.com/science/article/pii/S0305440384710314

54. Price TD, Burton JH, Fullagar PD, Wright LE, Buikstra JE, Tiesler V. Strontium isotopes and the study of human mobility in ancient Mesoamerica. Latin American Antiquity. 2008;19: 167–180. doi:10.2307/25478222

55. Hodell DA, Quinn RL, Brenner M, Kamenov G. Spatial variation of strontium isotopes (87Sr/86Sr) in the Maya region: a tool for tracking ancient human migration. Journal of Archaeological Science. 2004;31: 585–601. doi:10.1016/j.jas.2003.10.009

56. Pacheco-Forés SI, Gordon GW, Knudson KJ. Expanding radiogenic strontium isotope baseline data for central Mexican paleomobility studies. PLOS ONE. 2020;15: e0229687. doi:10.1371/journal.pone.0229687

57. Copeland SR, Cawthra HC, Fisher EC, Lee-Thorp JA, Cowling RM, le Roux PJ, et al. Strontium isotope investigation of ungulate movement patterns on the Pleistocene Paleo-Agulhas Plain of the Greater Cape Floristic Region, South Africa. Quaternary Science Reviews. 2016;141: 65–84. doi:10.1016/j.quascirev.2016.04.002

58. Kootker LM, van Lanen RJ, Kars H, Davies GR. Strontium isoscapes in The Netherlands. Spatial variations in 87Sr/86Sr as a proxy for palaeomobility. Journal of Archaeological Science: Reports. 2016;6: 1–13. doi:10.1016/j.jasrep.2016.01.015

59. Laffoon JE, Sonnemann TF, Shafie T, Hofman CL, Brandes U, Davies GR. Investigating human geographic origins using dual-isotope (87Sr/86Sr, δ18O) assignment approaches. PLOS ONE. 2017;12: e0172562. doi:10.1371/journal.pone.0172562

60. Scaffidi BK, Knudson KJ. An archaeological strontium isoscape for the prehistoric Andes: understanding population mobility through a geostatistical meta-analysis of archaeological 87Sr/86Sr values from humans, animals, and artifacts. Journal of Archaeological Science. 2020;117: 105121. doi:10.1016/j.jas.2020.105121

61. Willmes M, Bataille CP, James HF, Moffat I, McMorrow L, Kinsley L, et al. Mapping of bioavailable strontium isotope ratios in France for archaeological provenance studies. Applied Geochemistry. 2018;90: 75–86. doi:10.1016/j.apgeochem.2017.12.025

62. Bataille CP, von Holstein ICC, Laffoon JE, Willmes M, Liu X-M, Davies GR. A bioavailable strontium isoscape for Western Europe: A machine learning approach. Cotton J, editor. PLoS ONE. 2018;13: e0197386. doi:10.1371/journal.pone.0197386

63. Moreiras Reynaga DK, Millaire J-F, Chávez Balderas X, Román Berrelleza JA, López Luján L, Longstaffe FJ. Building Mexican isoscapes: oxygen and hydrogen isotope data of meteoric water sampled across Mexico. Data in Brief. 2021;36: 107084. doi:10.1016/j.dib.2021.107084

64. Wassenaar LI, Van Wilgenburg SL, Larson K, Hobson KA. A groundwater isoscape (δD, δ18O) for Mexico. Journal of Geochemical Exploration. 2009;102: 123–136. doi:10.1016/j.gexplo.2009.01.001

65. Moreiras Reynaga DK, Millaire J-F, Chávez Balderas X, Román Berrelleza JA, López Luján L, Longstaffe FJ. Residential patterns of Mexica human sacrifices at Mexico-Tenochtitlan and Mexico-Tlatelolco: Evidence from phosphate oxygen isotopes. Journal of Anthropological Archaeology. 2021;62: 101296. doi:10.1016/j.jaa.2021.101296

66. Knudson KJ, Price TD. Utility of multiple chemical techniques in archaeological residential mobility studies: case studies from Tiwanaku- and Chiribaya-affiliated sites in the Andes. Am J Phys Anthropol. 2007;132: 25–39. doi:10.1002/ajpa.20480

67. Bataille CP, Crowley BE, Wooller MJ, Bowen GJ. Advances in global bioavailable strontium isoscapes. Palaeogeography, Palaeoclimatology, Palaeoecology. 2020;555: 109849. doi:10.1016/j.palaeo.2020.109849

68. Bataille CP, Brennan SR, Hartmann J, Moosdorf N, Wooller MJ, Bowen GJ. A geostatistical framework for predicting variability in strontium concentrations and isotope ratios in Alaskan rivers. Chemical Geology. 2014;389: 1–15. doi:10. 1016/j.chemgeo.2014.08.030

69. Bataille CP, Bowen GJ. Mapping 87Sr/86Sr variations in bedrock and water for large scale provenance studies. Chemical Geology. 2012;304–305: 39–52. doi:10.1016/j.chemgeo.2012.01.028

70. Laffoon JE, Davies GR, Hoogland MLP, Hofman CL. Spatial variation of biologically available strontium isotopes (87Sr/86Sr) in an archipelagic setting: a case study from the Caribbean. Journal of Archaeological Science. 2012;39: 2371–2384. doi:10.1016/j.jas.2012.02.002

71. West JB, Hurley JM, Dudás FÖ, Ehleringer JR. The Stable Isotope Ratios of Marijuana. II. Strontium Isotopes Relate to Geographic Origin. Journal of Forensic Sciences. 2009;54: 1261–1269. doi:10.1111/j.1556-4029.2009.01171.x

72. Le Corre M, Dargent F, Grimes V, Wright J, Côté SD, Reich MS, et al. An ensemble machine learning bioavailable strontium isoscape for Eastern Canada. Geiss CE, editor. FACETS. 2025;10: 1–17. doi:10.1139/facets-2024-0180

73. Wang X, Bocksberger G, Arandjelovic M, Agbor A, Angedakin S, Aubert F, et al. Strontium isoscape of sub-Saharan Africa allows tracing origins of victims of the transatlantic slave trade. Nat Commun. 2024;15: 10891. doi:10.1038/s41467-024-55256-0

74. Wunder MB. Using Isoscapes to Model Probability Surfaces for Determining Geographic Origins. Isoscapes. Springer, Dordrecht; 2010. pp. 251–270. doi:10.1007/978-90-481-3354-3_12

75. Vlam M, Boeschoten LE, Van Der Sleen P, Adzkia U, Boom A, Bouka G, et al. Evaluating the Potential of Oxygen Isoscapes for Tropical Timber Tracing. SSRN; 2024. doi:10.2139/ssrn.5036136

76. Boeschoten LE, Vlam M, Sass-Klaassen U, Meyer-Sand BRV, Boom A, Bouka GUD, et al. Stable isotope ratios in wood show little potential for sub-country origin verification in Central Africa. Forest Ecology and Management. 2023;544: 121231. doi:10.1016/j.foreco.2023.121231

77. Meinshausen N. Quantile regression forests. Journal of Machine Learning Research. 2006;7: 983–999.

78. Blum JD, Taliaferro EH, Weisse MT, Holmes RT. Changes in Sr/Ca, Ba/Ca and 87Sr/86Sr ratios between trophic levels in two forest ecosystems in the northeastern U.S.A. Biogeochemistry. 2000;49: 87–101.

79. de Cserna Z. An outline of the geology of Mexico. In: Bally AW, Palmer AR, editors. The geology of North America--an overview. Boulder, CO: Geological Society of America; 1989. pp. 233–264.

80. Ortega-Gutiérrez F. La evolucion tectonica premisisipica del sur de México. Universidad National Autónoma de México Instituto de Geología Revista. 1981;5: 140–157.

81. Ferrusquía-Villafranca I. Geology of Mexico: a synopsis. In: Ramamoorthy TP, Bye R, Lot A, Fa J, editors. Biological diversity of Mexico: origins and distribution. New York: Oxford University Press; 1993. pp. 3–107.

82. Morán-Zenteno D. The geology of the Mexican republic. Cincinnati, OH: American Association of Petroleum Geologists; 1994.

83. Cano N, Camprubí A, González-Partida E. Metallogeny of the state of Oaxaca, Mexico. Journal of Maps. 2023;19: 2151384. doi:10.1080/17445647.2022.2151384

84. Servicio Geológico Mexicano. Carta geológico-minera Oaxaca E14-9, Oaxaca y Puebla. Pachuca, Hidalgo: Servicio Geológico Mexicano; 2000. Available: https://mapserver.sgm.gob.mx/Cartas_Online/geologia/99_E14-9_GM.pdf

85. Servicio Geológico Mexicano. Carta geológico-minera Zaachila E14-12, Oaxaca. Pachuca, Hidalgo: Servicio Geológico Mexicano; 2000. Available: https://mapserver.sgm.gob.mx/Cartas_Online/geologia/100_E14-12_GM.pdf

86. Reynolds AC, Quade J, Betancourt JL. Strontium isotopes and nutrient sourcing in a semi-arid woodland. Geoderma. 2012;189–190: 574–584. doi:10.1016/j.geoderma.2012.06.029

87. Feinman GM, Nicholas LM. Perspectives on dietary variability in the Classic-period Valley of Oaxaca. In: Pérez Rodríguez V, Morell-Hart S, King SM, editors. Mesquite pods to mezcal: 10,000 years of Oaxacan cuisines. Austin, TX: University of Texas Press; 2024. pp. 99–122.

88. Grimstead DN, Nugent S, Whipple J. Why a standardization of strontium isotope baseline environmental data is needed and recommendations for methodology. Advances in Archaeological Practice. 2017;5: 184–195. doi:10.1017/aap.2017.6

89. Runia LexT. Strontium and calcium distribution in plants: effect on palaeodietary studies. Journal of Archaeological Science. 1987;14: 599–608. doi:10.1016/0305-4403(87)90078-1

90. Romaniello SJ, Field MP, Smith HB, Gordon GW, Kim MH, Anbar AD. Fully automated chromatographic purification of Sr and Ca for isotopic analysis. J Anal At Spectrom. 2015;30: 1906–1912. doi:10.1039/C5JA00205B

91. National Institute of Standards and Technology, editor. Certificate of Analysis Standard Reference Material 987. Department of Commerce, United States of America; 2007.

92. Ma J, Wei G, Liu Y, Ren Z, Xu Y, Yang Y. Precise measurement of stable (δ 88/86Sr) and radiogenic (87Sr/86Sr) strontium isotope ratios in geological standard reference materials using MC-ICP-MS. Chin Sci Bull. 2013;58: 3111–3118. doi:10.1007/s11434-013-5803-5

93. Galler P, Limbeck A, Boulyga SF, Stingeder G, Hirata T, Prohaska T. Development of an on-line flow injection Sr/matrix separation method for accurate, high-throughput determination of Sr isotope ratios by multiple collector-inductively coupled plasma-mass spectrometry. Anal Chem. 2007;79: 5023–5029. doi:10.1021/ac070307h

94. Aubert D, Probst A, Stille P, Viville D. Evidence of hydrological control of Sr behavior in stream water (Strengbach catchment, Vosges mountains, France). Applied Geochemistry. 2002;17: 285–300. doi:10.1016/S0883-2927(01)00080-4

95. Tricca A, Stille P, Steinmann M, Kiefel B, Samuel J, Eikenberg J. Rare earth elements and Sr and Nd isotopic compositions of dissolved and suspended loads from small river systems in the Vosges mountains (France), the river Rhine and groundwater. Chemical Geology. 1999;160: 139–158.

96. Wadleigh MA, Veizer J, Brooks C. Strontium and its isotopes in Canadian rivers: Fluxes and global implications. Geochimica et Cosmochimica Acta. 1985;49: 1727–1736. doi:10.1016/0016-7037(85)90143-7

97. Brun P, Zimmermann NE, Hari C, Pellissier L, Karger DN. CHELSA-BIOCLIM+ A novel set of global climate-related predictors at kilometre-resolution. EnviDat; 2022. p. 13194139533312 bytes, 423097 bytes. doi:10.16904/ENVIDAT.332

98. Karger DN, Conrad O, Böhner J, Kawohl T, Kreft H, Soria-Auza RW, et al. Climatologies at high resolution for the earth's land surface areas. Sci Data. 2017;4: 170122. doi:10.1038/sdata.2017.122

99. Hengl T, Jesus JM de, Heuvelink GBM, Gonzalez MR, Kilibarda M, Blagotić A, et al. SoilGrids250m: Global gridded soil information based on machine learning. PLOS ONE. 2017;12: e0169748. doi:10.1371/journal.pone.0169748

100. Hartmann J, Moosdorf N. The new global lithological map database GLiM: A representation of rock properties at the Earth surface. Geochemistry, Geophysics, Geosystems. 2012;13. doi:10.1029/2012GC004370

101. Mooney WD, Laske G, Masters TG. CRUST 5.1: A global crustal model at 5° × 5°. Journal of Geophysical Research: Solid Earth. 1998;103: 727–747. doi:10.1029/97JB02122

102. Gelaro R, McCarty W, Suárez MJ, Todling R, Molod A, Takacs L, et al. The Modern-Era Retrospective Analysis for Research and Applications, Version 2 (MERRA-2). 2017 [cited 17 Mar 2025]. doi:10.1175/JCLI-D-16-0758.1

103. Chipman HA, George EI, McCulloch RE. BART: Bayesian additive regression trees. The Annals of Applied Statistics. 2010;4: 266–298. doi:10.1214/09-AOAS285

104. Freiwald C. Maya migration networks: Reconstructing population movement in the Belize River valley during the Late and Terminal Classic. Ph.D., The University of Wisconsin - Madison. 2011. Available: https://www.proquest.com/docview/886460450/abstract/F82F1B17A9DA43E7PQ/1

Información de apoyo

S1 Lista de verificación. Lista de verificación de inclusión en investigaciones globales.

S1 Fig. Resultados de PCA que identifican los principales factores ambientales que impulsan los patrones isotópicos de estroncio en Mesoamérica.

S2 Fig. Intervalos de confianza superior e inferior del 95% para los rangos de predicción de $^{87}Sr/^{86}Sr$ en el isopaisaje mesoamericano.

S1 Apéndice. Identificación de contaminación por fertilizantes agrícolas en muestras de plantas modernas de San Martín Tilcajete.
