# Supplementary material for: Radiogenic strontium isotope variability in the Valley of Oaxaca: A predictive isoscape for Mesoamerican paleomobility studies
Source: PLoS One. 2025 Dec 10;20(12):e0338628. doi: 10.1371/journal.pone.0338628 (PMC12694802; doi:10.1371/journal.pone.0338628)
Supplement: Attachment — Submitted filename: PONE-D-25-23485_reviewer.pdf [file pone.0338628.s006.pdf]

PLOS ONE

Radiogenic strontium isotope variability in the Valley of Oaxaca: A predictive isoscape for Mesoamerican paleomobility studies

--Manuscript Draft--

**Manuscript Number:**	PONE-D-25-23485
**Article Type:**	Research Article
**Full Title:**	Radiogenic strontium isotope variability in the Valley of Oaxaca: A predictive isoscape for Mesoamerican paleomobility studies
**Short Title:**	Radiogenic strontium isotope variability in the Valley of Oaxaca
**Corresponding Author:**	Sofía I. Pacheco-Forés<br>University of Minnesota Twin Cities: University of Minnesota<br>Minneapolis, Minnesota UNITED STATES OF AMERICA
**Keywords:**	radiogenic strontium; isoscapes; Bayesian Additive Regression Trees; ancient Mesoamerica; Valley of Oaxaca
**Abstract:**	Radiogenic strontium (⁸⁷Sr/⁸⁶Sr) isotope analysis is a well-established method for reconstructing the mobility of human populations in the past and present. Baseline ⁸⁷Sr/⁸⁶Sr data are fundamental to the method, as Sr varies across the landscape according to local geology and geoenvironmental factors. The method’s application within studies of ancient Mesoamerican paleomobility, however, has concentrated on two key regions despite its potential broader relevance across greater Mesoamerica. This is due in part to a lack of available baseline ⁸⁷Sr/⁸⁶Sr data for the region at large. Using the Valley of Oaxaca as a case study, we use Bayesian Additive Regression Trees (BART) to generate a locally calibrated predictive ⁸⁷Sr/⁸⁶Sr isoscape model of Mesoamerica in general and the Valley of Oaxaca in particular. We integrate (1) observed ⁸⁷Sr/⁸⁶Sr data from modern plant samples (<i>n</i>=95) from 17 sites across the Valley, (2) a compiled database of continental North and South American ⁸⁷Sr/⁸⁶Sr data, (3) geological bedrock maps, and (4) high resolution spatial data on geoenvironmental Sr covariates to iteratively develop and test a high performing predictive model for Mesoamerica, highlighting the importance of regional calibration in developing predictive ⁸⁷Sr/⁸⁶Sr isoscapes. Our results indicate that though overlap exists, ⁸⁷Sr/⁸⁶Sr can be used to detect migration within the Valley of Oaxaca as well as between the Valley and greater Mesoamerica. We then apply our isoscape to previously published human ⁸⁷Sr/⁸⁶Sr data from Monte Albán, Oaxaca to demonstrate how our model’s explicit quantification of uncertainty in local ⁸⁷Sr/⁸⁶Sr ranges allows for more nuanced interpretation of paleomobility in archaeological samples.
**Order of Authors:**	Sofía I. Pacheco-Forés, Ph.D.
	Nicolas Gauthier, Ph.D.
	Lacey B. Carpenter, Ph.D.
	Gwyneth Gordon, Ph.D.
	Kelly J. Knudson, Ph.D.
**Additional Information:**	
**Question**	**Response**
**Financial Disclosure**<br><br>Enter a financial disclosure statement that describes the sources of funding for the work included in this submission. Review	Yes

Powered by Editorial Manager® and ProduXion Manager® from Aries Systems Corporation

the submission guidelines for detailed requirements. View published research articles from *PLOS ONE* for specific examples.<br><br>This statement is required for submission and **will appear in the published article** if the submission is accepted. Please make sure it is accurate.<br><br>**Funded studies**<br>Enter a statement with the following details:<br>• Initials of the authors who received each award<br>• Grant numbers awarded to each author<br>• The full name of each funder<br>• URL of each funder website<br>• Did the sponsors or funders play any role in the study design, data collection and analysis, decision to publish, or preparation of the manuscript?<br><br>Did you receive funding for this work?	
Please add funding details.<br>as follow-up to "**Financial Disclosure**<br><br>Enter a financial disclosure statement that describes the sources of funding for the work included in this submission. Review the submission guidelines for detailed requirements. View published research articles from *PLOS ONE* for specific examples.<br><br>This statement is required for submission and **will appear in the published article** if the submission is accepted. Please make sure it is accurate.	This research was funded by the National Science Foundation (2013155229 and 1744335 awarded to SIPF; 2409068 awarded to NG and SIPF). The funders had no role in study design, data collection and analysis, decision to publish, or preparation of the manuscript.

**Funded studies**<br>Enter a statement with the following details:<br>• Initials of the authors who received each award<br>• Grant numbers awarded to each author<br>• The full name of each funder<br>• URL of each funder website<br>• Did the sponsors or funders play any role in the study design, data collection and analysis, decision to publish, or preparation of the manuscript?<br><br>Did you receive funding for this work?"	
Please select the country of your main research funder (please select carefully as in some cases this is used in fee calculation).<br>as follow-up to "**Financial Disclosure**<br><br>Enter a financial disclosure statement that describes the sources of funding for the work included in this submission. Review the submission guidelines for detailed requirements. View published research articles from *PLOS ONE* for specific examples.<br><br>This statement is required for submission and **will appear in the published article** if the submission is accepted. Please make sure it is accurate.<br><br>**Funded studies**<br>Enter a statement with the following details:<br>• Initials of the authors who received each award<br>• Grant numbers awarded to each author<br>• The full name of each funder<br>• URL of each funder website<br>• Did the sponsors or funders play any role in the study design, data collection and analysis, decision to publish, or preparation of the manuscript?<br><br>Did you receive funding for this work?"	UNITED STATES - US
**Competing Interests**	The authors have declared that no competing interests exist.

Use the instructions below to enter a competing interest statement for this submission. On behalf of all authors, disclose any competing interests that could be perceived to bias this work—acknowledging all financial support and any other relevant financial or non-financial competing interests.<br><br>This statement is required for submission and **will appear in the published article** if the submission is accepted. Please make sure it is accurate and that any funding sources listed in your Funding Information later in the submission form are also declared in your Financial Disclosure statement.<br><br>View published research articles from *PLOS ONE* for specific examples.<br><br>**NO authors have competing interests**<br><br>Enter: *The authors have declared that no competing interests exist.*<br><br>**Authors with competing interests**<br><br>Enter competing interest details beginning with this statement:<br><br>*I have read the journal's policy and the authors of this manuscript have the following competing interests: [insert competing interests here]*<br><br>* typeset	
**Ethics Statement**<br><br>Enter an ethics statement for this submission. This statement is required if the study involved:<br><br>• Human participants<br>• Human specimens or tissue<br>• Vertebrate animals or cephalopods<br>• Vertebrate embryos or tissues	Field research was conducted under Oficio núm. 401.B(4)19.2016/36/1020 and Oficio núm. 401.B(4)19.2016/36/1065 from the Consejo de Arqueología of the Mexican Instituto Nacional de Antropología e Historia. Collected plant samples were imported to the United States for analysis under United States Department of Agriculture Animal and Plant Health Inspection Service permits PCIP-17-00469 and PCIP-18-00287.

Powered by Editorial Manager® and ProduXion Manager® from Aries Systems Corporation

• Field research<br><br>Write "N/A" if the submission does not require an ethics statement.<br><br>General guidance is provided below. Consult the submission guidelines for detailed instructions. **Make sure that all information entered here is included in the Methods section of the manuscript.**	

**Format for specific study types**<br><br>**Human Subject Research (involving human participants and/or tissue)**<br>• Give the name of the institutional review board or ethics committee that approved the study<br>• Include the approval number and/or a statement indicating approval of this research<br>• Indicate the form of consent obtained (written/oral) or the reason that consent was not obtained (e.g. the data were analyzed anonymously)<br><br>**Animal Research (involving vertebrate animals, embryos or tissues)**<br>• Provide the name of the Institutional Animal Care and Use Committee (IACUC) or other relevant ethics board that reviewed the study protocol, and indicate whether they approved this research or granted a formal waiver of ethical approval<br>• Include an approval number if one was obtained<br>• If the study involved *non-human primates*, add *additional details* about animal welfare and steps taken to ameliorate suffering<br>• If anesthesia, euthanasia, or any kind of animal sacrifice is part of the study, include briefly which substances and/or methods were applied<br><br>**Field Research**<br><br>Include the following details if this study involves the collection of plant, animal, or other materials from a natural setting:<br>• Field permit number<br>• Name of the institution or relevant body that granted permission	
**Data Availability**<br><br>Authors are required to make all data underlying the findings described fully available, without restriction, and from the time of publication. PLOS allows rare exceptions to address legal and ethical concerns. See the PLOS Data Policy and FAQ for detailed information.	Yes - all data are fully available without restriction

Powered by Editorial Manager® and ProduXion Manager® from Aries Systems Corporation

A Data Availability Statement describing where the data can be found is required at submission. Your answers to this question constitute the Data Availability Statement and **will be published in the article**, if accepted.<br><br>**Important:** Stating 'data available on request from the author' is not sufficient. If your data are only available upon request, select 'No' for the first question and explain your exceptional situation in the text box.<br><br>Do the authors confirm that all data underlying the findings described in their manuscript are fully available without restriction?	
**Describe where the data may be found in full sentences. If you are copying our sample text, replace any instances of XXX with the appropriate details.**<br><br>• If the data are **held or will be held in a public repository**, include URLs, accession numbers or DOIs. If this information will only be available after acceptance, indicate this by ticking the box below. For example: *All XXX files are available from the XXX database (accession number(s) XXX, XXX.).*<br>• If the data are all contained **within the manuscript and/or Supporting Information files**, enter the following: *All relevant data are within the manuscript and its Supporting Information files.*<br>• If neither of these applies but you are able to provide **details of access elsewhere**, with or without limitations, please do so. For example:<br><br>*Data cannot be shared publicly because of [XXX]. Data are available from the XXX Institutional Data Access / Ethics Committee (contact via XXX) for researchers who meet the criteria for access to confidential data.*<br><br>*The data underlying the results presented in the study are available from (include the name of the third party*	All relevant data are within the manuscript. Associated code is available at https://github.com/flmnh-ai/oaxaca-isoscape

and contact information or URL).<br>• This text is appropriate if the data are owned by a third party and authors do not have permission to share the data.<br><br>* typeset	
Additional data availability information:	

Powered by Editorial Manager® and ProduXion Manager® from Aries Systems Corporation

Manuscript

Click here to access/download;Manuscript;Manuscript 4.29.25.docx

Radiogenic strontium isotope variability in the Valley of Oaxaca: A predictive isoscape for Mesoamerican paleomobility studies

Sofía I. Pacheco-Forés[1¶*], Nicolas Gauthier[2¶*], Lacey B. Carpenter[3], Gwyneth Gordon[4], Kelly J. Knudson[5]

[1]Department of Anthropology, University of Minnesota, Twin Cities, Minneapolis, Minnesota, United States of America

[2]Florida Museum of Natural History, University of Florida, Gainesville, Florida, United States of America

[3]Department of Anthropology, State University of New York, Buffalo, Buffalo, New York, United States of America

[4]School of Earth and Space Exploration, Arizona State University, Tempe, Arizona, United States of America

[5]Center for Bioarchaeological Research, School of Human Evolution and Social Change, Arizona State University, Tempe, Arizona, United States of America

*Corresponding authors

Emails: sipf@umn.edu (SIPF); nicolas.gauthier@ufl.edu (NG)

[¶]These authors contributed equally to this work.

Abstract

Radiogenic strontium ($^{87}Sr/^{86}Sr$) isotope analysis is a well-established method for reconstructing the mobility of human populations in the past and present. Baseline $^{87}Sr/^{86}Sr$ data are fundamental to the method, as Sr varies across the landscape according to local geology and geoenvironmental factors. The method's application within studies of ancient Mesoamerican paleomobility, however, has concentrated on two key regions despite its potential broader relevance across greater Mesoamerica. This is due in part to a lack of available baseline $^{87}Sr/^{86}Sr$ data for the region at large. Using the Valley of Oaxaca as a case study, we use Bayesian Additive Regression Trees (BART) to generate a locally calibrated predictive $^{87}Sr/^{86}Sr$ isoscape model of Mesoamerica in general and the Valley of Oaxaca in particular. We integrate (1) observed $^{87}Sr/^{86}Sr$ data from modern plant samples (n=95) from 17 sites across the Valley, (2) a compiled database of continental North and South American $^{87}Sr/^{86}Sr$ data, (3) geological bedrock maps, and (4) high resolution spatial data on geoenvironmental Sr covariates to iteratively develop and test a high performing predictive model for Mesoamerica, highlighting the importance of regional calibration in developing predictive $^{87}Sr/^{86}Sr$ isoscapes. Our results indicate that though overlap exists, $^{87}Sr/^{86}Sr$ can be used to detect migration within the Valley of Oaxaca as well as between the Valley and greater Mesoamerica. We then apply our isoscape to previously published human $^{87}Sr/^{86}Sr$ data from Monte Albán, Oaxaca to demonstrate how our model's explicit quantification of uncertainty in local $^{87}Sr/^{86}Sr$ ranges allows for more nuanced interpretation of paleomobility in archaeological samples.

Introduction

There is a long tradition of archaeological and bioarchaeological research in the Valley of Oaxaca [1–5]. Much of this research focuses on the emergence of one of the earliest state-level societies in the Americas—the multi-ethnic Zapotec state of Monte Albán in the Valley of Oaxaca of southern Mexico during the Late Formative period (300-100 BCE). Archaeologists have documented marked shifts in material culture and settlement patterns during this time period, leading them to infer that both regional and interregional migration may have played a role in the development of the Zapotec state [6]. Although the last 40 years have seen an explosion in biogeochemical studies of human skeletal remains to directly test Mesoamerican migration hypotheses [7], these paleomobility studies have focused overwhelmingly on central Mexico and the central Maya lowlands [8]. Very few biogeochemical studies of migration exist in the Valley of Oaxaca [but see 9–12]. This dearth of biogeochemical investigations of migration in the Valley of Oaxaca may be due at least in part to the lack of published isotopic baseline data for the region.

The development of spatially predictive maps of environmental isotopic variation called isoscapes has greatly aided paleomobility research in regions lacking isotope baseline data [13]. In this article, we examine radiogenic strontium ($^{87}Sr/^{86}Sr$) variability across the Valley of Oaxaca. We present $^{87}Sr/^{86}Sr$ values from a total of 95 modern plant samples from 17 sites across the Valley of Oaxaca. We use the resulting dataset, together with previously compiled $^{87}Sr/^{86}Sr$ data, to develop and test a predictive isoscape model tuned to Mesoamerica in general and the Valley of Oaxaca in particular. Our goal is to provide a high performing predictive $^{87}Sr/^{86}Sr$ baseline isoscape for use in paleomobility studies in the Valley of Oaxaca.

Assessing migration through biogeochemistry in the Valley of Oaxaca

The Valley of Oaxaca is made up of a series of three interconnected valleys forming a triangular Y-shaped area (Fig 1). The Etla arm extends to the northwest, the Tlacolula arm extends east, and the Ocotlán-Zimatlán arm extends to the south. During the Rosario (700-500 BCE) phase of the Middle Formative period, three major settlements—San José Mogote, Yeguih, and San Martín Tilcajete—emerged in each of the three branches of the Valley of Oaxaca. Monte Albán was founded in the center of the Valley at the beginning of the Early Monte Albán I phase (500 – 300 BCE) [1,3,14]. Monte Albán continued to expand its influence through the Late Monte Albán I phase (300 BCE – 100 BCE) and the Monte Albán II phase (100 BCE – 200 CE) [15,16].

Fig 1. The Valley of Oaxaca. The three branches of the Valley of Oaxaca and their corresponding regional centers alongside a map of the 17 sites in the Valley of Oaxaca from which we collected modern plants for $^{87}Sr/^{86}Sr$ analysis.

Both regional and interregional migration played a crucial role in the models for the sociopolitical development of the Valley of Oaxaca. Marked population growth and demographic shifts within the Valley coinciding with the founding of Monte Albán have been attributed to the arrival of migrants into the region as well as the movement of peoples within the Valley itself [1,3,17–22]. Migration has also been implicated in competing population centers' resistance to Monte Albán's expansionism [16,23]. Following Monte Albán's consolidation of power, migration was essential to the state's administrative strategies and trade networks [9,24–26].

Despite this, most archaeological evidence of these migrations remains indirect, implied through settlement patterns, population estimates, and stylistic similarities in architecture and material culture. These data sources can offer insights into the role migration played in the region's cultural development, but the most direct way to evaluate this is to first identify the migrants themselves.

Biogeochemical methods allow archaeologists to directly investigate migration on an individual scale. Hydrogen (δ^{2}H), stable oxygen (δ^{18}O), sulfur (δ^{34}S), radiogenic strontium (^{87}Sr/^{86}Sr), and lead ($^{206/204}$Pb, $^{207/204}$Pb, $^{208/204}$Pb, $^{207/206}$Pb) isotope analyses provide archaeologists with well-established means of reconstructing the residential histories of past peoples [e.g., 27–31]. These isotope systems vary geographically. Hydrogen and oxygen isotopes vary according to regional hydrology and reflect environmental factors such as elevation, temperature, humidity, and latitude [27,32–35]. In contrast, sulfur, radiogenic strontium, and lead isotope ratios vary according to local geology, reflecting the age and composition of geologic bedrock [36–40]. These isotopes are incorporated into human calcified tissues via consumed food, imbibed liquids, and inhaled dust particles and will reflect the isotopic signature of the region where an individual lived during tissue development [34,41–47]. Archaeologists can thus detect individual migrants by identifying changes in isotopic signatures between tissues forming at different times over the life course and/or divergences between isotopic signatures in these tissues and the burial environment, as these differences would signify residence in isotopically distinct regions [e.g., 10,48].

While biogeochemical analyses of paleomobility are well established and have been very successful in detecting migration across ancient Mesoamerica, most studies focus on the central Mexican city of Teotihuacan and the Maya region [8]. Only a handful of studies to date have

attempted to directly identify migrants in the Valley of Oaxaca, and all but one of these have focused exclusively on Monte Albán (Table 1). Cumulatively, the four studies examine paleomobility among 75 individuals in the Valley of Oaxaca. Only 8% of these individuals were identified as migrants. This itself is surprising, as the preceding discussion indicates there is ample archaeological evidence of both regional and interregional migration in the Valley of Oaxaca.

Table 1. Biogeochemical studies of migration in the Valley of Oaxaca[a]

Site	Time period	Method	*n* migrants identified/ *N* sampled individuals	Data source
Monte Albán	500 BCE – 1520 CE	$\delta^{18}O$ in bone phosphate	0/16	[12]
Monte Albán	100 BCE – 550 CE	$^{87}Sr/^{86}Sr$ in enamel and bone apatite	1/5	[10]
Monte Albán	100 BCE – 1350 CE	$\delta^{18}O$ in enamel carbonate	5/38	[9]
Teposcolula Yucundaa	1295-1634 CE	$\delta^{18}O$ in enamel carbonate	0/16	[11]

[a]This table only includes studies of individuals excavated from secure archaeological contexts. It does not include data from Olivares Flores [49], as that analysis uses $\delta^{18}O$ analysis of enamel

carbonate from *n*=7 incised skulls of unknown provenience to argue that six of the seven individuals likely originate from the Valley of Oaxaca.

Identifying migrants using biogeochemical baselines and isoscapes

The scarcity of biogeochemically-identified migrants in the Valley of Oaxaca despite the long history of archaeological investigation in the region and its status as one of the first multi-ethnic state-level societies to emerge in the Americas may be due to the lack of published biogeochemical baseline data. Biogeochemical baselines are essential in establishing expectations for characterizing “local” isotopic signatures [50]. If an individual’s observed isotopic values diverge sufficiently from a “local” biogeochemical baseline, the individual is designated a “non-local” and thus most likely a migrant. These biogeochemical baselines are created through the analysis of environmental (typically plant or faunal) samples from a site of interest and the application of basic summary statistics to establish thresholds for local $^{87}Sr/^{86}Sr$ variation [36,50]. Archaeologists have used the mean $^{87}Sr/^{86}Sr$ value of environmental samples from a site ± two standard deviations [51,52] or their interquartile range [53] to create biogeochemical baselines defining local $^{87}Sr/^{86}Sr$ variation at a particular site.

None of the previous biogeochemical studies of paleomobility in the Valley of Oaxaca, however, employ biogeochemical baselines. Instead, they identify statistical outliers among the observed human values as possible migrants. While there is an abundance of published environmental isotopic data for other regions of Mexico [e.g., 54,55], such data are scarce for the Valley of Oaxaca. This makes the creation of relevant regional biogeochemical baselines difficult. Furthermore, although biogeochemical baselines provide an objective means of inferring an individual’s mobility status relative to expected local $^{87}Sr/^{86}Sr$ values, the statistical

means of establishing thresholds to delimit local variation is arbitrary [50] and does not account for the non-normal distribution of $^{87}Sr/^{86}Sr$ values across the landscape [53]. As such, the studies cited in Table 1 represent a promising start to investigating the role of migration in the cultural development of the Valley of Oaxaca and the Zapotec state, but more work remains to be done.

Isoscapes and paleomobility research in the Valley of Oaxaca

In response to the uneven regional coverage of published biogeochemical baseline data and the limitations of threshold approaches to biogeochemical baselines, researchers have increasingly adopted the use of isoscapes to fill in the blanks. Isoscapes are spatially predictive maps of environmental isotopic variation [13]. Some isoscapes are generated through the geostatistical interpolation (e.g., kriging) of observed isotopic data [56–60]. Geostatistical isoscapes are based on spatial autocorrelation, the idea that points geographically closer to each other on a map tend to be environmentally (for H and O) or geologically (for S, Sr, and Pb) similar. In addition to using empirical isotopic data, these geostatistical models can incorporate covariates such as hydrological or geological maps to further constrain predicted isotope variability. Because isotopes are often non-normally distributed across the landscape, however, these geostatistical isoscapes may falsely represent the actual variability in the selected isotope system [61]. As such, they must be used with caution.

Geostatistical isoscapes using empirical $\delta^{18}O$ values from water samples taken across Mexico show the predicted range of clinal $\delta^{18}O$ variability across Mexico [62,63]. Moreiras Reynaga and colleagues [64] divide this clinal variation into five distinct or slightly overlapping zones of $\delta^{18}O$ values. The zone containing the Valley of Oaxaca covers a large swathe of western Mexico, including most of Mexico's Pacific Coast [64]. The fact that such a massive geographical area shares similar $\delta^{18}O$ values means there is a high probability of missing

potential migrants in the Valley of Oaxaca from other parts of the Pacific Coast. The inability of $\delta^{18}O$ analyses to address questions of regional mobility within the Valley of Oaxaca and their limited utility in assessing interregional mobility across much of western Mexico has led some scholars to posit that $\delta^{18}O$ isotopes may not have a sufficient range of variation to detect the presence of non-local individuals within the Valley [9,49]. This may explain why three of the four existing paleomobility studies in the Valley of Oaxaca using $\delta^{18}O$ identify migrants with varying success (Table 1). Targeting other isotope systems with greater variability in the Valley of Oaxaca and/or using multiple isotope systems rather than relying on a single system [e.g., 65] present the best path forward for biogeochemical studies of migration in the Valley of Oaxaca.

Mechanistic models represent another approach to generating isoscapes. This method employs first principles of isotope geochemistry to predict the evolution of a targeted isotope system in a given region [66]. The model's predictions are then compared to published isotope baseline data to test its performance [67–70]. While promising, mechanistic model isoscapes are limited by insufficiently detailed, inaccurate, or inconsistent geologic maps that in turn produce poorly performing models. In addition, estimates from geological maps must take into consideration soil weathering, plant rooting depth and vertical soil variation, and the relative bioavailability of elements which will cause significant modification of strontium isotopes between underlying lithology and the peoples that live in the region. Soil weathering and vertical soil variation are strongly dependent on climate. The interplay between these controlling parameters is poorly understood, making predictive models from purely mechanistic factors problematic.

The challenges posed by both empirical geostatistical and mechanistic model isoscapes have led to the emergence of a hybrid process-based statistical approach to creating isoscapes.

This method uses random forest regression, a decision tree-based machine learning algorithm. A decision tree works by repeatedly splitting data based on predictor values (such as bedrock age or rainfall) to create increasingly homogeneous groups. For example, a simple tree might first divide samples based on bedrock age (older or younger than 100 million years), then further divide each group based on elevation, and so on until it can accurately predict strontium values. Decision trees are intuitive but can be unstable and prone to overfitting when used alone. Random forests and similar methods address this limitation by combining hundreds of trees, each considering different combinations of predictors. This ensemble approach produces more robust predictions than a single tree. Hybrid process-based isoscapes incorporate empirical isotope baseline data along with other geoenvironmental covariates used in geostatistical isoscapes into the mechanistic modeling framework to predict isotope values [61,66,71,72]. This hybrid approach simultaneously overcomes limitations in both geostatistical and mechanistic isoscape models, yielding higher performing models that accurately account for non-normal distributions of isotopes across targeted regions of interest.

Strontium isotopes and the Valley of Oaxaca

The Valley of Oaxaca represents a particularly attractive candidate for a process-based statistical $^{87}Sr/^{86}Sr$ isoscape. Bioavailable radiogenic strontium varies according to the age and composition of local bedrock, but factors such as the erosion and preferential weathering of bedrock, as well as the addition of material from windblown dust and sea spray can significantly alter $^{87}Sr/^{86}Sr$ values in the environment that are ultimately incorporated into human tissues [for an in-depth discussion of Sr systematics and bioavailable $^{87}Sr/^{86}Sr$ cycling within ecosystems, see 36,37,73]. The Valley of Oaxaca is located in the Sierra Madre Sur, the most geologically complex morphotectonic province of Mexico [74]. It contains some the oldest Precambrian

metamorphic geological formations in Mexico overlain with isolated Paleozoic and Mesozoic sedimentary deposits and Cenozoic volcanic formations [74–76]. The Valley thus promises a high level of intra- and interregional variation in $^{87}Sr/^{86}Sr$ isotopes.

Although no hybrid isoscapes for any isotope system exist in the Valley of Oaxaca, Bataille and colleagues [66] present a $^{87}Sr/^{86}Sr$ isoscape model that uses random forest regression to predict bioavailable $^{87}Sr/^{86}Sr$ variability globally. Their groundbreaking model integrates compiled empirical $^{87}Sr/^{86}Sr$ data and auxiliary covariates known to affect bioavailable $^{87}Sr/^{86}Sr$ with a mechanistic model framework based on geological bedrock. Bataille and colleagues test the model using empirical $^{87}Sr/^{86}Sr$ data. The model performs well in predicting observed $^{87}Sr/^{86}Sr$ values in data-rich regions but performs poorly when predicting observed $^{87}Sr/^{86}Sr$ values in under-sampled regions with complex geologies. The authors conclude that the addition of just tens to hundreds of $^{87}Sr/^{86}Sr$ data points in under-sampled geologically complex areas significantly improves the regional accuracy of the global model. Moreover, in some cases, a regionally specific isoscape model eschewing global bioavailable $^{87}Sr/^{86}Sr$ data is necessary to overcome such models' strong predictive bias towards data-rich regions.

A regionally specific isoscape model could be invaluable in furthering paleomobility research in the Valley of Oaxaca. Unfortunately, very little empirical $^{87}Sr/^{86}Sr$ baseline data in the Valley of Oaxaca exist. Price and colleagues provide some site-level averages of $^{87}Sr/^{86}Sr$ values, but the entire Valley is represented by just 10 samples from five sites, and no raw data are published [53]. To address this gap in the research, we present $^{87}Sr/^{86}Sr$ baseline data for 95 modern plant samples from 17 sites across the Valley of Oaxaca. We use this dataset, along with a compiled database of continental North and South American $^{87}Sr/^{86}Sr$ data, to iteratively train and test a regional Mesoamerican $^{87}Sr/^{86}Sr$ isoscape model focused on the Valley of Oaxaca. Our

goal is to provide a high-quality locally calibrated predictive $^{87}Sr/^{86}Sr$ isoscape that will lay the groundwork for increased biogeochemical investigation of paleomobility in the Valley of Oaxaca.

Materials and Methods

Sample collection

We opportunistically collected a total of 95 modern plant samples from 17 archaeological sites across the Valley of Oaxaca, with an average of five plant samples per site. We targeted plants for baseline sampling as $^{87}Sr/^{86}Sr$ in plants reflect a more consistent average of the local bioavailable $^{87}Sr/^{86}Sr$ in a given ecosystem than whole soils, which can vary greatly even in a small area due to the distinct strontium concentrations and weathering profiles of minerals in the underlying bedrock [36]. Even so, plants with varied rooting depths have access to different sources of Sr (i.e., bedrock vs wind-borne dust), introducing variation to their observed $^{87}Sr/^{86}Sr$ values [77]. As such, we sampled both shallow and deep rooting depths whenever possible to account for these different local $^{87}Sr/^{86}Sr$ sources. Furthermore, to better simulate sources of $^{87}Sr/^{86}Sr$ in prehispanic diets, we prioritized sampling plant taxa that contributed to ancient diets in the Valley of Oaxaca, such as maguey (*Agave* spp.) and prickly pears (*Opuntia ficus*), or plant taxa with similar rooting depths to ancient staples such as maize (*Zea mays*) that we were unable to sample [78,79]. Additionally, we sought to avoid sampling plants treated with fertilizers or irrigation water, as these could skew signatures of local bioavailable strontium with non-local sources of strontium.

Collected samples primarily constituted plant leaves or seed pods, not whole plants. We recorded Universal Transverse Mercator (UTM) coordinate and elevation data for each plant sample using a hand-held GPS unit. The Mexican Instituto Nacional de Antropología e Historia

(INAH) does not require specific permissions to collect modern plant samples from the study sites. Furthermore, no endangered or protected plant species were collected for the study. Samples were imported to the Arizona State University Archaeological Chemistry Laboratory under permits granted to Pacheco-Forés from the United States Department of Agriculture Animal and Plant Health Inspection Service (permits PCIP-17-00469 and PCIP-18-00287).

Biogeochemical methods

Plant samples were prepared at the Arizona State University Archaeological Chemistry Laboratory. When possible, prehispanic diets were simulated through the manual isolation and analysis of edible components of dried plants [80]. Plant samples were prepared rinsed with 18.2 MΩ Millipore water to remove adhering dirt and ashed in a furnace for 10 hours at 800˚C. Approximately 25 mg of plant ash was digested in 2 mL of concentrated nitric and hydrochloric acid (HNO_3 +3HCl) at 50˚C for 24 hours. This aggressive leach does not break down the silica tetrahedra structure of most silicate minerals, leaving much of the soil in a solid form while prioritizing the release of bioavailable strontium within plants. Leach solution was evaporated, and sample precipitates were redissolved in concentrated nitric acid and diluted to a 2M stock solution.

Dissolved samples were analyzed at the Metals, Environmental, and Terrestrial Analytical Laboratory (METAL) at Arizona State University. An aliquot was taken for elemental concentration analysis on a Thermo Fisher Scientific iCAP quadrupole inductively coupled plasma mass spectrometer (Q-ICP-MS). Strontium was then separated with a Prep*FAST* system [81]. Strontium was isolated from the sample matrix using Elemental Scientific, Inc. supplied Sr-Ca ion exchange resin (Part CF-MC-SrCa-1000) and ultrapure 5M nitric acid (HNO_3). To

determine chemical recovery, 22% of samples were measured on the Q-ICP-MS before and after chemical purification. Average chemistry yield was 85%.

The remaining portion of each Sr cut from the Prep*FAST* was dried down in a Teflon beaker and digested with concentrated HNO_3 and 30% hydrogen peroxide (H_2O_2) to remove organics from the resin. Once digested, samples were again dried down and reconstituted with 0.32 M HNO_3. Using concentration information from the Q-ICP-MS, the samples were diluted with 0.32 M HNO_3 to a calculated constant concentration of 50 ppb Sr. Samples were then run on a Thermo-Finnigan Neptune multi-collector inductively coupled plasma mass spectrometer (MC-ICP-MS).

Data were collected by measuring 60 simultaneous ratios integrating 4.194 seconds each. Samples were corrected for on-peak blanks, and in-line correction of the contributions of ^{84}Kr on ^{84}Sr and ^{86}Kr on ^{86}Sr using ^{83}Kr/^{84}Kr ratio of 0.201750 and ^{83}Kr/^{86}Kr ratio of 0.664533, after instrumental mass bias correction using a normalizing ^{88}Sr/^{86}Sr ratio of 8.375209. Samples were analyzed in two different analytical sessions. Typical sensitivity was 36 V on ^{88}Sr with a 50 ppb Sr solution, with ^{83}Kr values of 0.0001 V. ^{85}Rb voltages for samples were typically <0.001 V due to the low Rb/Sr initial ratios of the samples and effective chemical purification, but all data was interference-corrected using an ^{85}Rb/^{87}Rb ratio of 2.588960, normalized to ^{88}Sr/^{86}Sr as above. Ratio outliers two standard deviations outside the mean were removed using a Matlab 2D-mathematical correction routine written by Stephen Romaniello. Typical internal ^{87}Sr/^{86}Sr two standard error (SE) precision was ≤1e-5.

Sequences included bracketing concentration-matched SRM 987 standards. SRM 987 was run as a bracketing standard with a measured value of ^{87}Sr/^{86}Sr=0.710227±0.000119 (2σ, *n*=57). Each analytical session included a sequence incorporating SRM 987 standard in a range

of variable concentrations to verify the accuracy of $^{87}Sr/^{86}Sr$ values for samples; reported values are all fall within the range for accurate $^{87}Sr/^{86}Sr$ values within the range of error of the bracketing standards [82]. In addition, SRM 987 doped with calcium up to a ratio of Ca/Sr of 500 was run to simulate the accuracy and precision of isotope ratios in poorly purified samples with low yields. SRM 987 run at 50% concentration doped to a Ca/Sr of 500 was run as a check standard with a measured value of $^{87}Sr/^{86}Sr$=0.710218 ± 0.000138 (2σ, *n*=17). IAPSO seawater (Ocean Scientific International Ltd., Havant, UK) as a secondary check standard had a measured value of 0.709136±0.000142 (2σ, *n*=13), within error of the published value of 0.709182 ±0.000004 [83]. NIST 1400 purified in parallel with samples had a measured value of 0.713084 ±0.000129 (2σ, *n*=10), within error of the published value of 0.713150 ±0.000016 [84]. Approximately 5% of samples (*n*=7) were run in triplicate, with an average precision on individual measurements of ±0.000008 (2σ).

Isoscape model development and testing

We compiled strontium isotope data for North and South America from existing global and regional syntheses [8,66,71] and our newly collected measurements from the Valley of Oaxaca. The compiled dataset included 7,271 measurements from humans, animals, plants, soil, and water sources across diverse environments. Human data from the Caribbean and Mesoamerica Biogeochemical Isotope Overview (CAMBIO, version 1.2) were filtered to exclude individuals identified as non-local from the compiled dataset [8]. Water samples present a special challenge for strontium modeling, as they can reflect non-local signals due to transport across watersheds [68,85–87]. Rather than excluding these samples entirely, we incorporated a categorical predictor variable indicating sample type (e.g., water, plant, etc.), allowing the model to appropriately handle the different uncertainty profiles associated with each sample type.

We filtered the data to exclude samples with $^{87}Sr/^{86}Sr$ values outside the range of 0.703 to 0.780 to remove potential outliers and focus model training on the most relevant range for archaeological interpretation within Mesoamerica [see 8]. We applied a logit transformation to the strontium isotope ratios, a standard approach for bounded variables that helps normalize the distribution and reduces the influence of extreme values on model training.

We assembled a set of spatial environmental predictors based on established relationships with strontium isotope distribution patterns [66,68]. These predictors included updated climate variables from CHELSA V2.1 [88,89], soil properties at multiple depths such as bulk density and pH [90], geological variables including basement age and lithological classifications [66,91,92], aerosol deposition data from MERRA-2 reanalysis [93], and terrain variables. We performed domain-specific principal component analyses to reduce the dimensionality of these predictors, creating a minimal set of uncorrelated climate, soil, geological, and aerosol variables. This approach resulted in 24 predictor variables for the final model. Detailed PCA results are provided in S1 Fig.

We employed a Bayesian Additive Regression Trees (BART) approach [94] for modeling strontium isoscapes. While traditional random forests have been used in previous isoscapes [e.g., 61,66], they have limitations in quantifying prediction uncertainty, which is essential for distinguishing between local and non-local individuals in archaeological contexts. BART extends random forests and similar ensemble decision tree methods by incorporating Bayesian statistical methods to prevent overfitting. The key advantage of BART for archaeological applications is its ability to naturally quantify uncertainty in its predictions. Rather than providing only a single predicted value for each location, BART generates a range of plausible values with associated probabilities. This approach gives archaeologists crucial information

about the relative confidence of local strontium ranges and by extension the reliability of migrant identification. In regions with higher prediction uncertainty, wider ranges must be considered for defining "local" signatures, while areas with lower uncertainty allow for more precise determinations.

We evaluated model performance using both root mean square error (RMSE) and R-squared (R^2) values on held-out test data. Our iterative evaluation approach involved first training a model on our compiled dataset from North and South America, excluding all data (both compiled and observed) from Mesoamerica. We then tested this model's prediction accuracy on compiled data from Mesoamerica, using this process to fine tune the model's parameters while assessing the model's ability to extrapolate to a new region of interest. Then we retrained this model with the compiled data from Mesoamerica and tested it on the newly collected Oaxacan samples to assess its ability to extrapolate within our region of interest. For the final isoscape, we retrained the model incorporating all compiled data from North, Central, and South America and our new Valley of Oaxaca samples. We compare the model predictions and performance metrics at each step in the process to assess the stability of the model's predictions and the added value of including increasingly local samples in the training set. We then applied this final model across the Valley of Oaxaca study area to create spatially explicit predictions of $^{87}Sr/^{86}Sr$ values with associated uncertainty estimates. We assessed variable importance by calculating the frequency with which each variable was used in the BART model [94], allowing us to identify the major environmental factors driving strontium isotope patterns in the region (S1 Fig). The resulting isoscape provides both predicted $^{87}Sr/^{86}Sr$ values and associated uncertainty estimates at a 1 km spatial resolution. These predictions serve as a

baseline against which archaeological samples can be compared to distinguish local from non-local individuals in the region.

Results

Biogeochemical results

Table 2 reports observed $^{87}Sr/^{86}Sr$ values in sampled plants. In the Valley of Oaxaca, $^{87}Sr/^{86}Sr$ values ranged from 0.704603 to 0.711827, with a mean of $^{87}Sr/^{86}Sr=0.706817\pm0.001401$ (1σ, $n=95$).

379 **Table 2. Provenance data, sample details, and $^{87}Sr/^{86}Sr$ values for analyzed Valley of Oaxaca plant samples.**

Laboratory ID	Site	UTM-E[a]	UTM-N[a]	Elevation (masl)	Species	Plant Rooting Depth	$^{87}Sr/^{86}Sr$
ACL-10588	Atzompa	736019	1889532	1817	Agave spp.	shallow	0.707450
ACL-10589	Atzompa	736018	1889624	1983	Yucca filifera	deep	0.707555
ACL-10590	Atzompa	737791	1889737	1884	Jacaranda mimosifolia	shallow	0.707557
ACL-10591	Atzompa	735986	1889778	1893	Acacia farnesiana	deep	0.707356
ACL-10592	Atzompa	735987	1889716	1884	Opuntia ficus	shallow	0.707154
ACL-10593	Atzompa	735988	1889655	1710	Jacaranda mimosifolia	shallow	0.707674
ACL-9642	Dainzú	760073	1881555	1617	Poa spp.	shallow	0.705759
ACL-9643	Dainzú	760018	1881603	1615	Opuntia ficus	shallow	0.705911
ACL-9644	Dainzú	759992	1881588	1611	Prosopis spp.	deep	0.705724
ACL-9645	Dainzú	760168	1881588	1637	Agave spp.	shallow	0.705270
ACL-10598	Etla Reyes	732518	1903608	1640	Opuntia ficus	shallow	0.706463
ACL-10599	Etla Reyes	732509	1904407	1640	Prosopis spp.	deep	0.706382

ACL-10600	Etla Reyes	732528	1902685	1640	Prosopis spp.	deep	0.706388
ACL-10601	Etla Reyes	732410	1902715	1643	Opuntia ficus	shallow	0.706400
ACL-10602	Etla Reyes	732410	1902684	1643	Opuntia ficus	shallow	0.706339
ACL-10603	Etla Reyes	732520	1900840	1640	Agave spp.	shallow	0.706397
ACL-9629	Hierve el Agua	790184	1866831	1771	Agave spp.	shallow	0.707431
ACL-9630	Hierve el Agua	790123	1866492	1711	Agave spp.	shallow	0.707377
ACL-9631	Hierve el Agua	790343	1866610	1653	Agave spp.	shallow	0.707448
ACL-9632	Hierve el Agua	790333	1866995	1764	Agave spp.	shallow	0.707428
ACL-9639	Lambityeco	767086	1878105	1613	Yucca filifera	deep	0.705545
ACL-9640	Lambityeco	767040	1878109	1609	Agave spp.	shallow	0.705730
ACL-9641	Lambityeco	767017	1878173	1604	Prosopis spp.	deep	0.705515
ACL-9633	Mitla	781307	1873516	1694	Agave spp.	shallow	0.705960
ACL-9634	Mitla	781270	1873523	1703	Poa spp.	shallow	0.706405
ACL-9635	Mitla	781208	1873324	1689	Yucca filifera	deep	0.705913
ACL-9636	Mitla	781289	1873332	1691	Opuntia ficus	shallow	0.705914
ACL-9637	Mitla	781204	1873272	1695	Pachycereus weberi	shallow	0.706094

ACL-9638	Mitla	781032	1873257	1686	Poa spp.	shallow	0.706143
ACL-9095	Mitla Fortaleza	779009	1873969	1832	Yucca filifera	deep	0.705918
ACL-9096	Mitla Fortaleza	779009	1873969	1832	Stenocereus thurberi	shallow	0.705937
ACL-9097	Mitla Fortaleza	779009	1873969	1832	Agave spp.	shallow	0.705900
ACL-9098	Mitla Fortaleza	779009	1873969	1832	Agave spp.	shallow	0.705907
ACL-9099	Mitla Fortaleza	779009	1873969	1832	Agave spp.	shallow	0.706536
ACL-9100	Mitla Fortaleza	779009	1873969	1832	Agave spp.	shallow	0.705906
ACL-9091	Monte Albán	737631	1885941	1940	Agave spp.	shallow	0.708005
ACL-9092	Monte Albán	737631	1885941	1940	Agave spp.	shallow	0.707878
ACL-9093	Monte Albán	737631	1885941	1940	Leucaena leucocephala	deep	0.707691
ACL-9094	Monte Albán	737631	1885941	1940	Opuntia ficus	shallow	0.707726
ACL-10594	Monte Albán	737301	1886010	1881	Agave spp.	shallow	0.707787
ACL-10595	Monte Albán	737449	1886012	1929	Prosopis spp.	deep	0.707754
ACL-10596	Monte Albán	737448	1886073	1929	Agave spp.	shallow	0.707459
ACL-10597	Monte Albán	737478	1886104	1926	Jacaranda mimosifolia	shallow	0.707584
ACL-10618	Ocotlán	755149	1856142	1475	Pachycereus weberi	shallow	0.705086

ACL-10619	Ocotlán	750794	1856090	1475	Agave spp.	shallow	0.705144
ACL-10620	Ocotlán	750558	1855995	1570	Acacia farnesiana	deep	0.705119
ACL-9611	San José Mogote	733521	1899560	1615	Prosopis spp.	deep	0.706327
ACL-9612	San José Mogote	733501	1899500	1616	Opuntia ficus	shallow	0.706104
ACL-9613	San José Mogote	733643	1899366	1617	Agave spp.	shallow	0.706062
ACL-9614	San José Mogote	733622	1899359	1606	Prosopis spp.	deep	0.706574
ACL-9615	San José Mogote	733802	1899534	1608	Opuntia ficus	shallow	0.706260
ACL-9616	San José Mogote	733748	1899569	1612	Prosopis spp.	deep	0.706270
ACL-9083	San Martín Tilcajete	745202	1867422	1623	Acacia farnesiana	deep	0.708259[b]
ACL-9084	San Martín Tilcajete	745202	1867422	1623	Agave spp.	shallow	0.709700[b]
ACL-9085	San Martín Tilcajete	745202	1867422	1623	Opuntia ficus	shallow	0.709633[b]
ACL-9086	San Martín Tilcajete	745202	1867422	1623	Acacia farnesiana	deep	0.710458[b]
ACL-9087	San Martín Tilcajete	745202	1867422	1623	Yucca filifera	deep	0.707351[b]
ACL-9088	San Martín Tilcajete	745202	1867422	1623	Opuntia ficus	shallow	0.707237[b]
ACL-9089	San Martín Tilcajete	745202	1867422	1623	Acacia farnesiana	deep	0.708389[b]
ACL-9090	San Martín Tilcajete	745202	1867422	1623	Dichondra argentea	shallow	0.708531[b]

ACL-10604	San Martín Tilcajete	743657	1866063	1545	Agave spp.	shallow	0.708404[b]
ACL-10605	San Martín Tilcajete	743183	1866057	1579	Ipomoea arborescens	shallow	0.711827[b]
ACL-10606	San Martín Tilcajete	743005	1866148	1561	Protium copal	deep	0.711590[b]
ACL-10607	San Martín Tilcajete	742561	1866081	1612	Pachycereus weberi	shallow	0.709421[b]
ACL-10614	Santa Catarina Minas	754393	1857425	1612	Acacia farnesiana	deep	0.705971
ACL-10615	Santa Catarina Minas	755036	1858109	1615	Agave spp.	shallow	0.705627
ACL-10616	Santa Catarina Minas	755065	1858202	1631	Opuntia ficus	shallow	0.705870
ACL-10617	Santa Catarina Minas	755120	1858541	1612	Pachycereus weberi	shallow	0.706535
ACL-10621	Santa Rosa de Lima	745904	1856217	1527	Agave spp.	shallow	0.705435
ACL-10623	Santa Rosa de Lima	745830	1854925	1533	Jacaranda mimosifolia	shallow	0.705533
ACL-10624	Santa Rosa de Lima	747431	1854851	1527	Pachycereus weberi	shallow	0.705443
ACL-10608	Santo Tomás Jalieza	748316	1865287	1609	Agave spp.	shallow	0.704875
ACL-10609	Santo Tomás Jalieza	748255	1865409	1585	Ipomoea arborescens	shallow	0.704603
ACL-10610	Santo Tomás Jalieza	748193	1865655	1585	Pinus spp.	deep	0.704952
ACL-10611	Santo Tomás Jalieza	748136	1867930	1615	Protium copal	deep	0.706335
ACL-10612	Santo Tomás Jalieza	748162	1865746	1597	Acacia farnesiana	deep	0.704809

ACL-10613	Santo Tomás Jalieza	748162	1865716	1588	Agave spp.	shallow	0.704783
ACL-9617	Suchilquitongo	726283	1910455	1786	Agave spp.	shallow	0.706545
ACL-9618	Suchilquitongo	726451	1910539	1804	Prosopis spp.	deep	0.706795
ACL-9619	Suchilquitongo	726293	1910688	1810	Agave spp.	shallow	0.706946
ACL-9620	Suchilquitongo	726292	1910717	1808	Opuntia ficus	shallow	0.706938
ACL-9621	Suchilquitongo	726289	1910739	1809	Agave spp.	shallow	0.707002
ACL-9622	Suchilquitongo	726309	1910503	1815	Agave spp.	shallow	0.706519
ACL-9646	Yagul	771545	1876618	1683	Opuntia ficus	shallow	0.705875
ACL-9647	Yagul	771529	1876694	1690	Opuntia ficus	shallow	0.705920
ACL-9648	Yagul	771582	1876791	1700	Pachycereus weberi	shallow	0.706043
ACL-9649	Yagul	771649	1876788	1699	Opuntia ficus	shallow	0.705815
ACL-9650	Yagul	771826	1876657	1727	Opuntia ficus	shallow	0.705874
ACL-9651	Yagul	771800	1876708	1740	Agave spp.	shallow	0.705855
ACL-9623	Zaachila	739726	1875665	1525	Prosopis spp.	deep	0.708502
ACL-9624	Zaachila	739719	1875686	1535	Prosopis spp.	deep	0.708782
ACL-9625	Zaachila	739735	1875711	1532	Agave spp.	shallow	0.708395

ACL-9626	Zaachila	739722	1875763	1529	Agave spp.	shallow	0.707974
ACL-9627	Zaachila	739718	1875801	1514	Opuntia ficus	shallow	0.708248
ACL-9628	Zaachila	739699	1875702	1526	Agave spp.	shallow	0.708384

[a]All UTM coordinates are in UTM Zone 14N.

[b]We identified these $^{87}Sr/^{86}Sr$ values as implausibly high for the region and likely the artifact of the use of agricultural fertilizers in the collection area. See further discussion below and in S1 Appendix. These samples were excluded from the final model training.

Isoscape model performance

The initial continental strontium model, trained on compiled data from North and South America but excluding Mexico and Central America, achieved moderate predictive performance when applied to the broader Mesoamerican context, with a logit-transformed RMSE of 0.427 and R^2 of 0.57. Adding Mesoamerican data substantially improved the model's ability to predict within the region, reducing RMSE to 0.175 (logit-transformed) and increasing R^2 to 0.889, highlighting the importance of regional calibration. When tested on unseen Oaxaca samples, the model incorporating all continental data (RMSE 0.304, R^2 0.28) performed similarly to the continental-only model (RMSE 0.344, R^2 0.35) and both were better than the geological bedrock benchmark (RMSE 0.533, R^2 0.34). This stepwise improvement demonstrates how increasingly localized data enhances model performance, suggesting that fine-scale isoscape modeling benefits substantially from targeted regional sampling alongside continental-scale data integration (Table 3).

Table 3. Model performance metrics across different training datasets and test regions.

	Mesoamerica Test Data[a]		Oaxaca Plant Samples Test Data[a]	
Training Data	RMSE	R^2	RMSE	R^2
North and South America (excluding Mesoamerica[b])	0.427 / 0.0017	0.57 / 0.38	0.344 / 0.0012	0.35 / 0.29
North and South America + Mesoamerica (excluding Oaxaca plants)	0.175 / 0.0011[d]	0.889 / 0.71[d]	0.304 / 0.0011	0.28 / 0.24
North and South America + Mesoamerica + Oaxaca plants	0.176 / 0.0011[d]	0.888 / 0.71[d]	0.225 / 0.0008[d]	0.59 / 0.57[d]

Bedrock-only benchmark[c]	0.539 / 0.0019	0.394 / 0.24	0.533 / 0.0026	0.34 / 0.28

[a]Values show RMSE (Root Mean Square Error) and R^2 (coefficient determination) in both logit-transformed and untransformed (original) $^{87}Sr/^{86}Sr$ units. Lower RMSE and higher R^2 values indicate better model performance.

[b]Mesoamerica is defined as any $^{87}Sr/^{86}Sr$ values from Mexico, Belize, Guatemala, Honduras, El Salvador, Nicaragua, and Costa Rica.

[c]The bedrock-only benchmark represents a geologically-informed baseline [from 66] for comparison with the machine learning models.

[d]Indicates measures of in-sample performance which may be over optimistic.

Predictive variables

Variable importance analysis revealed several key factors driving strontium isotope distribution patterns in the region. The most influential predictors were distance from the coast; basement age of underlying geology; the first principal component of the strontium bedrock model; climate principal components related to precipitation intensity and timing; aerosol variables related to carbon and sulfate abundance and dust concentration; and soil variables related to cation exchange capacity and texture (S1 Fig). These findings align with theoretical expectations for strontium cycling, where the basic bedrock geology is filtered through soil transport processes, climatic weathering, and aerosol deposition [66]. Notably, the high-resolution distance from the coast was favored over the low-resolution sea salt aerosol map in the model, suggesting the clear added value of high-resolution predictors, even if they are indirect proxies for “true” driving variables, at estimating local Sr baselines.

Spatial distribution of predicted values

The final calibrated model produced estimates of $^{87}Sr/^{86}Sr$ values across Mesoamerica ranging from 0.704 to 0.713 (Fig 2). The model revealed several distinct isotopic regions. Areas with elevated $^{87}Sr/^{86}Sr$ values (>0.709) were identified in the Maya Mountains, Yucatán Peninsula, and coastal ranges of Oaxaca, consistent with older geological formations in these regions and available published $^{87}Sr/^{86}Sr$ data [95]. Less radiogenic areas (<0.706) were observed across the Tuxtla Mountains in Veracruz and along a strip of the central volcanic axis, reflecting younger volcanic geology. Interestingly, the fine-tuned model incorporating Oaxaca plant samples reduced the estimated strontium isotope ratios across most of Mexico, only raising estimates slightly in the southern coastal regions of Oaxaca and Guerrero. On the whole, isoscape predicted values and ranges generally conform to published regional summary $^{87}Sr/^{86}Sr$ data across Mesoamerica [53] while providing a clearer picture of regional strontium spatial variability.

Fig 2. Final calibrated isoscape model of $^{87}Sr/^{86}Sr$ variation across Mesoamerica.

In the Valley of Oaxaca, our isoscape suggests that we should be able to detect the intravalley mobility suggested by archaeological settlement survey data (Fig 3). Although each arm of the Valley exhibits some overlap in predicted $^{87}Sr/^{86}Sr$ values, the eastern Tlacolula arm of the Valley is dominated by large areas of lower predicted $^{87}Sr/^{86}Sr$ values (~0.705-0.706), while the northwestern Etla arm has higher predicted $^{87}Sr/^{86}Sr$ values (~0.706-0.707), and the southern Ocotlán-Zimatlán arm features some of the highest predicted $^{87}Sr/^{86}Sr$ values within the Valley (~0.7075-0.708). Thus, while the isoscape certainly allows for the identification of non-

local individuals within the Valley of Oaxaca who may have originated from other parts of the Valley, the lack of mutually exclusive $^{87}Sr/^{86}Sr$ values between the arms limits our ability to geolocate from where exactly these non-locals originated.

Fig 3. Final calibrated $^{87}Sr/^{86}Sr$ isoscape model across the modern state of Oaxaca. Sites from which we collected plant samples with which to train the BART model are shown in white, with the exception of Monte Albán, which is shown in red.

Uncertainty patterns

The BART modeling approach provided native uncertainty estimates for all predictions across the isoscape (Fig 4). Consistent with previous studies, we found that strontium prediction uncertainty increased with the strontium ratio, with higher, more radiogenic ratios being associated with greater uncertainty due to increased variability in weathering and transport processes in regions with older, more radiogenic bedrock. Older bedrock has a greater variation in $^{87}Sr/^{86}Sr$ ratios between coexisting minerals, which have a different susceptibility to weathering. The match between model predictions and strontium observations was generally good, with no clear spatial structure in the residuals that would indicate significant unmodeled processes. However, we did observe lingering mismatches between modeled and observed isotope ratios in specific contexts. These discrepancies primarily occurred in human burial contexts near major bedrock transitions, where relatively high variability in strontium ratios would be expected within the local catchment. Examples include individuals from the Chalillo Reservoir on the Macal River at the edge of the isotopically distinct Maya Mountains, as well as individuals from regional centers like Cholula, a central Mexican pilgrimage site, and Tikal, a

dominant Classic period Maya city-state. During our analysis, we also detected a group of samples from our Oaxaca plants at San Martín Tilcajete with implausibly high strontium ratios for the region (Table 2). Further investigation revealed these samples likely reflect contamination from modern agricultural fertilizer application (S1 Appendix) and were excluded from the final model training.

Fig 4. Native uncertainty estimates for all predicted $^{87}Sr/^{86}Sr$ values across the isoscape.

Discussion

Our results indicate much less clear regional isotopic distinctions that might be suggested by underlying bedrock alone, reflecting the varying influence of climate and soil processes on bioavailable $^{87}Sr/^{86}Sr$. The uncertainty in predictions, while reduced from the global model, remains relatively high compared to the range of most values. Assessments of migration based on strontium isotopes may thus be more variable than expected from bedrock geology alone.

Using the predictive $^{87}Sr/^{86}Sr$ isoscape

To demonstrate the utility of our predictive $^{87}Sr/^{86}Sr$ isoscape model, we applied it to published human $^{87}Sr/^{86}Sr$ values (n=10) from Monte Albán [10]. The observations comprise paired $^{87}Sr/^{86}Sr$ bone and enamel samples from five individuals dating from the Monte Albán II phase (100 BCE – 200 CE) and the Monte Albán III phase (200 – 500 CE). Price and colleagues [10] identify one individual's bone and tooth $^{87}Sr/^{86}Sr$ values as significantly lower than the other four Monte Albán individuals. The authors suggest this individual likely lived non-locally to Monte Albán during early childhood (enamel) and in the last ~10 years of life (bone). This

assessment, however, is not based on an evaluation of biogeochemical baseline $^{87}Sr/^{86}Sr$ data, as no previously published environmental $^{87}Sr/^{86}Sr$ data exist for Monte Albán.

Our model's explicit quantification of uncertainty allows for more nuanced interpretation of archaeological samples. For example, when evaluating individuals from Monte Albán, our model provides not just a point estimate for the "local" strontium signature, but a probability distribution that accounts for prediction uncertainty (Fig 4). This Bayesian approach offers a more robust framework for identifying potential migrants along a continuous spectrum of locality, as opposed to traditional threshold-based methods that construct a rigid binary of local/non-local. Because BART works by growing an ensemble of multiple decision trees, each prediction represents an array of predicted values, one from each tree in the ensemble. This distribution of predicted values makes it straightforward to calculate summary statistics after the fact, such as the mean, median, or upper and lower quantiles of the predicted value at any given pixel.

The isoscape predicts that local Monte Albán $^{87}Sr/^{86}Sr$ values range from 0.7069 to 0.7086, with a median value of $^{87}Sr/^{86}Sr$=0.7076. This range represents the interquartile range of the final BART model's posterior predictive distribution for $^{87}Sr/^{86}Sr$ values for Monte Albán (Fig 5) and is somewhat broader than a traditional threshold-based biogeochemical baseline generated from environmental $^{87}Sr/^{86}Sr$ values. For example, our observed Monte Albán plant $^{87}Sr/^{86}Sr$ values ranged from 0.707459 to 0.708005, with a mean of $^{87}Sr/^{86}Sr$=0.707736 ± 0.000168 (1σ, n=8). Though wider, the BART model produces a local range calibrated to actual geologic and environmental input strontium sources that more accurately capture $^{87}Sr/^{86}Sr$ cycling within the environment rather than arbitrarily imposed statistical thresholds independent of the broader environmental context. Furthermore, the predicted local range from our isoscape

model provides additional benefits over traditional statistical biogeochemical baselines generated from observed environmental $^{87}Sr/^{86}Sr$ values. Whereas we collected plant samples opportunistically (and therefore idiosyncratically) over a limited portion of the site, the isoscape provides a geographically standardized estimated local $^{87}Sr/^{86}Sr$ range for Monte Albán over a 1 km radius, allowing us to more accurately characterize local strontium values.

Fig 5. Posterior predictive distribution of expected "local" $^{87}Sr/^{86}Sr$ at Monte Albán from the final BART model.

When we apply the isoscape predicted local range to previously published Monte Albán human $^{87}Sr/^{86}Sr$ values, we come away with a more nuanced understanding of paleomobility at the site (Fig 6). While individuals previously categorized as locals (n=4) and non-locals (n=1) remain in these categories [10], their placement either comfortably within, or on the margins of, the Monte Albán predicted local range gives us a sense of the relative certainty or uncertainty of these designations. For example, among individuals with tissue samples falling within the predicted local range, Burial 34's femur is the least likely to be local given that part of the wide range of the $^{87}Sr/^{86}Sr$ measurement itself falls outside the bounds of the local Monte Albán range. In contrast, Burial 53's femur is the most likely of all the observed tissues to be local, as it has the narrowest $^{87}Sr/^{86}Sr$ measurement range and falls squarely within Monte Albán's predicted local range. Similarly, the non-local individual (Burial 26A) exhibits a bone value that falls into the lower extreme of Monte Albán's local range. This, coupled with the individual's burial in a Classic period tomb at Monte Albán, could indicate that the individual from Burial 26A lived locally at Monte Albán before their death for enough time that their bones started acclimating to

local $^{87}Sr/^{86}Sr$ values. We thus get a better sense of the certainty of our designation of “local” and “non-local” status among individuals interred at Monte Albán, recognizing that biogeochemical locality (i.e., falling within the Monte Albán predicted local range) does not necessarily equate cultural locality.

Fig 6. Previously published paired human enamel and bone samples from Monte Albán compared to the BART model predicted Monte Albán local range (grey shaded area). Each boxplot represents simulated draws for mean and standard deviation of each measurement based on $^{87}Sr/^{86}Sr$ and standard error data [from 10].

Conclusion

We created a BART predictive $^{87}Sr/^{86}Sr$ isoscape model of Mesoamerica and the Valley of Oaxaca, integrating novel and compiled empirical Mesoamerican $^{87}Sr/^{86}Sr$ data in addition to geological maps and geoenvironmental Sr covariate spatial data. The BART model’s improved performance over a global $^{87}Sr/^{86}Sr$ isoscape model based on geologic bedrock alone indicates both the importance of regional calibration in developing predictive isoscapes, as well as the importance of accounting for the varying influence of geoenvironmental factors such as climate and soil processes on bioavailable $^{87}Sr/^{86}Sr$. We anticipate that the model will be further improved with the incorporation of additional empirical $^{87}Sr/^{86}Sr$ values from across greater Mesoamerica, particularly in regions with high native uncertainty estimates.

Our isoscape indicates that clear $^{87}Sr/^{86}Sr$ variation exists within greater Mesoamerica and the Valley of Oaxaca. Although there is some overlap in $^{87}Sr/^{86}Sr$ values across the three branches of the Valley of Oaxaca, there is sufficient variation between them to detect intra-valley

migration, as well as migration between the Valley and greater Mesoamerica. By providing a high performing predictive $^{87}Sr/^{86}Sr$ isoscape for the Valley, we hope that biogeochemical investigations of migration in the region will expand beyond their initial focus on the Zapotec state capital of Monte Albán. The inclusion of all three branches of the Valley in the isoscape will allow researchers to consider questions about the scale of migration in the Valley and what role both intra- and interregional migration played in the formation and maintenance of the Zapotec state, as well as in the subsequent cultural development of the region.

Finally, the isoscape provides archaeologists with a more refined tool for the examination and discussion of past migratory patterns within ancient Mesoamerica. The BART model's standardized and geoenvironmentally informed predictions of expected "local" $^{87}Sr/^{86}Sr$ ranges for archaeological sites of interest provides clear advantages over the use of traditional statistical threshold-based approaches that do not account for the broader environmental context's effects on local $^{87}Sr/^{86}Sr$ variability. Moreover, the model's explicit quantification of uncertainty in predicted $^{87}Sr/^{86}Sr$ values provides archaeologists with crucial information about confidence in expected "local" $^{87}Sr/^{86}Sr$ ranges that directly impacts the reliability of migrant identification.

Acknowledgements

At Arizona State University's Archaeological Chemistry Laboratory, we are thankful to research apprentices Eric Flores, Kari Guilbault, Sparshee Naik, and Tajinder Virdee for assistance with sample processing. At the Metals, Environmental, and Terrestrial Analytical Laboratory, we are grateful for the assistance of Dr. Stephen Romaniello, Dr. Trevor Martin, and Natasha Zolotova.

References

1. Blanton RE, Kowalewski SA, Feinman GM, Appel J. Monte Alban's hinterland, part I: the prehispanic settlement patterns of the central and Southern parts of the Valley of Oaxaca, Mexico. Ann Arbor, MI: Museum of Anthropology, University of Michigan; 1982.

2. Caso A. Las tumbas de Monte Albán. Anales del Instituto Nacional de Antropología e Historia. 1933; 641–648.

3. Feinman GM, Nicholas LM. Settlement patterns of the Ejutla Valley, Oaxaca, Mexico: a diachronic macroscale perspective. Fieldiana Anthropology. 2013; 1–330.

4. Romero J. El material osteológico de Monte Albán, Mexico. Boletín Bibliográfico de Antropología Americana. 1949;12: 166–168.

5. Saville MH. Exploration of Zapotecan tombs in southern Mexico. American Anthropologist. 1899;1: 350–362. doi:10.1525/aa.1899.1.2.02a00080

6. Feinman GM, Blanton RE, Nicholas LM. The emergence of Monte Albán: a social innovation that lasted a millennium. 1st ed. In: Thurston TL, Fernandez-Gotz M, editors. Power from Below in Premodern Societies. 1st ed. Cambridge University Press; 2021. pp. 220–246. doi:10.1017/9781009042826.011

7. Spence MW, White CD. Mesoamerican bioarchaeology: past and future. Ancient Mesoamerica. 2010;20: 233–240. doi:10.1017/S0956536109990083

8. Ebert CE, Hixon SW, Buckley GM, George RJ, Pacheco-Forés SI, Palomo JM, et al. The Caribbean and Mesoamerica Biogeochemical Isotope Overview (CAMBIO). Sci Data. 2024;11: 349. doi:10.1038/s41597-024-03167-6

9. Casar I, Márquez L, Cienfuegos E, González Licón E, Somerville AD. Monte Alban and Teotihuacan connections: can stable isotope analysis of bone and enamel detect migration between two ancient Mesoamerican urban capitals? Archaeol Anthropol Sci. 2022;14: 220. doi:10.1007/s12520-022-01683-3

10. Price TD, Manzanilla LR, Middleton WD. Immigration and the ancient city of Teotihuacan in Mexico: a study using strontium isotope ratios in human bone and teeth. Journal of Archaeological Science. 2000;27: 903–913. doi:10.1006/jasc.1999.0504

11. Warinner C, García NR, Spores R, Tuross N. Disease, demography, and diet in early colonial New Spain: investigation of a sixteenth-century Mixtec cemetery at Teposcolula Yucundaa. Latin American Antiquity. 2012;23: 467–489.

12. White CD, Spence MW, Stuart-Williams HLQ, Schwarcz HP. Oxygen isotopes and the identification of geographical origins: the Valley of Oaxaca versus the Valley of Mexico. Journal of Archaeological Science. 1998;25: 643–655. doi:10.1006/jasc.1997.0259

13. Bowen GJ, West JB. Isotope landscapes for terrestrial migration research. Terrestrial Ecology. Elsevier; 2008. pp. 79–105. doi:10.1016/S1936-7961(07)00004-8

14. Blanton RE. Monte Albán: settlement patterns at the ancient Zapotec capital. New York, NY: Academic Press; 1978.

15. Redmond EM, Spencer CS. From raiding to conquest: warfare strategies and early state development in Oaxaca, Mexico. In: Arkush EN, Allen MW, editors. The archaeology of warfare: prehistory of raiding and conquest. Gainesville, FL: University Press of Florida; 2006. pp. 336–393.

16. Spencer CS, Redmond EM. Militarism, resistance, and early state development in Oaxaca, Mexico. Social Evolution & History. 2003;2: 25–70.

17. Blanton RE, Finsten L, Kowalewski SA, Feinman GM. Migration and population change in the prehispanic Valley of Oaxaca, Mexico. In: Mastache AG, Parsons JR, Santley RS, Serra Puche MC, editors. Arqueología mesoamericana: homenaje a William T Sanders. Mexico City: Instituto Nacional de Antropología e Historia; 1996. pp. 11–36.

18. Feinman GM, Kowalewski SA, Finsten L, Blanton RE, Nicholas LM. Long-term demographic change: a perspective from the Valley of Oaxaca. Journal of Field Archaeology. 1985;12: 333–362. doi:10.1179/jfa.1985.12.3.333

19. Flannery KV, Marcus J. Zapotec civilization: how urban society evolved in Mexico's Oaxaca Valley. London: Thames & Hudson; 1996.

20. Kowalewski SA, Feinman GM, Finsten L, Blanton RE, Nicholas LM. Monte Albán's hinterland, part II. Ann Arbor, MI: Museum of Anthropology, University of Michigan; 1989.

21. Marcus J. Woman's ritual in Formative Oaxaca: figurine making, divination, death and the ancestors. Ann Arbor, MI: University of Michigan Museum of Anthropology; 1998.

22. Nicholas LM, Feinman GM. The foundation of Monte Albán, intensification, and growth: coactive processes and joint production. Front Polit Sci. 2022;4: 805047. doi:10.3389/fpos.2022.805047

23. Spencer CS, Redmond EM. Primary state formation in Mesoamerica. Annu Rev Anthropol. 2004;33: 173–199. doi:10.1146/annurev.anthro.33.070203.143823

24. Elson CM. Excavations at Cerro Tilcajete: a Monte Albán II administrative center in the Valley of Oaxaca. Ann Arbor, MI: Museum of Anthropology, University of Michigan; 2007.

25. Márquez Morfín L, Negrete-Gutiérrez SS, Pérez-Flórez AM. A Teotihuacano in Monte Alban: life history in a domestic group from an osteobiographic approach. 1st ed. In: Wrobel GD, Cucina A, editors. Mesoamerican Osteobiographies. 1st ed. Gainesville, FL: University Press of Florida; 2024. pp. 138–150. doi:10.2307/jj.16430753.13

26. Spence MW. A Zapotec diaspora network in Classic-period central Mexico. In: Stein GJ, editor. The archaeology of colonial encounters: comparative perspectives. Santa Fe, NM: School of American Research Press; 2005. pp. 173–206.

27. Ehleringer JR, Bowen GJ, Chesson LA, West AG, Podlesak DW, Cerling TE. Hydrogen and oxygen isotope ratios in human hair are related to geography. Proceedings of the National Academy of Sciences. 2008;105: 2788–2793.

28. Ericson JE. Strontium isotope characterization in the study of prehistoric human ecology. Journal of Human Evolution. 1985;14: 503–514.

29. Gulson BL, Jameson WW, Gillings BR. Stable lead isotopes in teeth as indicators of past domicile-a potential new tool in forensic science? Journal of forensic sciences. 1997;42: 787–791.

30. Richards MP, Fuller BT, Hedges REM. Sulphur isotopic variation in ancient bone collagen from Europe: implications for human palaeodiet, residence mobility, and modern pollutant studies. Earth and Planetary Science Letters. 2001;191: 185–190. doi:10.1016/S0012-821X(01)00427-7

31. Schwarcz HP, Gibbs L, Knyf M. Oxygen isotope analysis as an indicator of place of origin. Snake Hill: an investigation of a military cemetery from the War of 1812. Toronto, ON: Dundurn Press; 1991. pp. 263–268.

32. Bowen GJ, Wassenaar LI, Hobson KA. Global application of stable hydrogen and oxygen isotopes to wildlife forensics. Oecologia. 2005;143: 337–348.

33. Dansgaard W. Stable isotopes in precipitation. Tellus. 1964;16: 436–468. doi:10.1111/j.2153-3490.1964.tb00181.x

34. Gat JR. Oxygen and hydrogen isotopes in the hydrologic cycle. Annual Review of Earth and Planetary Sciences. 1996;24: 225–262. doi:10.1146/annurev.earth.24.1.225

35. Luz B, Kolodny Y. Oxygen isotope variation in bone phosphate. Applied Geochemistry. 1989;4: 317–323. doi:10.1016/0883-2927(89)90035-8

36. Bentley RA. Strontium isotopes from the earth to the archaeological skeleton: a review. J Archaeol Method Theory. 2006;13: 135–187. doi:10.1007/s10816-006-9009-x

37. Faure G, Powell JL. Strontium isotope geology. New York, NY: Springer-Verlag; 1972.

38. Krouse H, Levinson A. Geographical trends of carbon and sulphur isotope abundances in human kidney stones. Geochimica et Cosmochimica Acta. 1984;48: 187–191. doi:10.1016/0016-7037 (84)90360-0

39. Nehlich O. The application of sulphur isotope analyses in archaeological research: a review. Earth-Science Reviews. 2015;142: 1–17. doi:10.1016/j.earscirev.2014.12.002

40. Stille P, Shields G. Radiogenic isotope geochemistry of sedimentary and aquatic systems. Berlin: Springer; 1997. doi:10.1007/BFb0117747

41. Flockhart DTT, Kyser TK, Chipley D, Miller NG, Norris DR. Experimental evidence shows no fractionation of strontium isotopes (87Sr/86Sr) among soil, plants, and herbivores: implications for tracking wildlife and forensic science. Isotopes in Environmental and Health Studies. 2015;51: 372–381. doi:10.1080/10256016.2015.1021345

42. Kamenov GD, Gulson BL. The Pb isotopic record of historical to modern human lead exposure. Science of The Total Environment. 2014;490: 861–870. doi:10.1016/j.scitotenv.2014.05.085

43. Longinelli A. Oxygen isotopes in mammal bone phosphate: a new tool for paleohydrological and paleoclimatological research? Geochimica et Cosmochimica Acta. 1984;48: 385–390. doi:10.1016/0016-7037(84)90259-X

44. Luz B, Kolodny Y, Horowitz M. Fractionation of oxygen isotopes between mammalian bone-phosphate and environmental drinking water. Geochimica et Cosmochimica Acta. 1984;48: 1689–1693. doi:10.1016/0016-7037(84)90338-7

45. Richards MP, Fuller BT, Sponheimer M, Robinson T, Ayliffe L. Sulphur isotopes in palaeodietary studies: a review and results from a controlled feeding experiment. International Journal of Osteoarchaeology. 2003;13: 37–45. doi:10.1002/oa.654

46. Sealy JC, van der Merwe NJ, Sillen A, Kruger FJ, Krueger HW. 87Sr/86Sr as a dietary indicator in modern and archaeological bone. Journal of Archaeological Science. 1991;18: 399–416. doi:10.1016/0305-4403(91)90074-Y

47. Turekian KK, Kulp JL. Strontium content of human bones. Science. 1956;124: 405–407.

48. Knudson KJ, Stanish C, Lozada Cerna MC, Faull KF, Tantaleán H. Intra-individual variability and strontium isotope measurements: A methodological study using 87Sr/86Sr data from Pampa de los Gentiles, Chincha Valley, Peru. Journal of Archaeological Science: Reports. 2016;5: 590–597. doi:10.1016/j.jasrep.2016.01.016

49. Olivares Flores E. Aspectos sobre dieta y procedencia de los cráneos esgrafiados de la Casa del Mendrugo, a partir de análisis de isótopos estables. M.A. thesis, Universidad Nacional Autónoma de México. 2021. Available: http://132.248.9.195/ptd2021/febrero/0809591/0809591.pdf

50. Price TD, Burton JH, Bentley RA. The characterization of biologically available strontium isotope ratios for the study of prehistoric migration. Archaeometry. 2002;44: 117–135. doi:10.1111/1475-4754.00047

51. Ezzo JA, Johnson CM, Price TD. Analytical perspectives on prehistoric migration: a case study from east-central Arizona. Journal of Archaeological Science. 1997;24: 447–466. doi:10.1006/jasc.1996.0129

52. Price TD, Johnson CM, Ezzo JA, Ericson J, Burton JH. Residential mobility in the prehistoric southwest United States: a preliminary study using strontium isotope analysis. Journal of Archaeological Science. 1994;21: 315–330.

53. Price TD, Burton JH, Fullagar PD, Wright LE, Buikstra JE, Tiesler V. Strontium isotopes and the study of human mobility in ancient Mesoamerica. Latin American Antiquity. 2008;19: 167–180. doi:10.2307/25478222

54. Hodell DA, Quinn RL, Brenner M, Kamenov G. Spatial variation of strontium isotopes (87Sr/86Sr) in the Maya region: a tool for tracking ancient human migration. Journal of Archaeological Science. 2004;31: 585–601. doi:10.1016/j.jas.2003.10.009

55. Pacheco-Forés SI, Gordon GW, Knudson KJ. Expanding radiogenic strontium isotope baseline data for central Mexican paleomobility studies. PLOS ONE. 2020;15: e0229687. doi:10.1371/journal.pone.0229687

56. Copeland SR, Cawthra HC, Fisher EC, Lee-Thorp JA, Cowling RM, le Roux PJ, et al. Strontium isotope investigation of ungulate movement patterns on the Pleistocene Paleo-Agulhas Plain of the Greater Cape Floristic Region, South Africa. Quaternary Science Reviews. 2016;141: 65–84. doi:10.1016/j.quascirev.2016.04.002

57. Kootker LM, van Lanen RJ, Kars H, Davies GR. Strontium isoscapes in The Netherlands. Spatial variations in 87Sr/86Sr as a proxy for palaeomobility. Journal of Archaeological Science: Reports. 2016;6: 1–13. doi:10.1016/j.jasrep.2016.01.015

58. Laffoon JE, Sonnemann TF, Shafie T, Hofman CL, Brandes U, Davies GR. Investigating human geographic origins using dual-isotope (87Sr/86Sr, δ18O) assignment approaches. PLOS ONE. 2017;12: e0172562. doi:10.1371/journal.pone.0172562

59. Scaffidi BK, Knudson KJ. An archaeological strontium isoscape for the prehistoric Andes: understanding population mobility through a geostatistical meta-analysis of archaeological 87Sr/86Sr values from humans, animals, and artifacts. Journal of Archaeological Science. 2020;117: 105121. doi:10.1016/j.jas.2020.105121

60. Willmes M, Bataille CP, James HF, Moffat I, McMorrow L, Kinsley L, et al. Mapping of bioavailable strontium isotope ratios in France for archaeological provenance studies. Applied Geochemistry. 2018;90: 75–86. doi:10.1016/j.apgeochem.2017.12.025

61. Bataille CP, von Holstein ICC, Laffoon JE, Willmes M, Liu X-M, Davies GR. A bioavailable strontium isoscape for Western Europe: A machine learning approach. Cotton J, editor. PLoS ONE. 2018;13: e0197386. doi:10.1371/journal.pone.0197386

62. Moreiras Reynaga DK, Millaire J-F, Chávez Balderas X, Román Berrelleza JA, López Luján L, Longstaffe FJ. Building Mexican isoscapes: oxygen and hydrogen isotope data of meteoric water sampled across Mexico. Data in Brief. 2021;36: 107084. doi:10.1016/j.dib.2021.107084

63. Wassenaar LI, Van Wilgenburg SL, Larson K, Hobson KA. A groundwater isoscape (δD, δ18O) for Mexico. Journal of Geochemical Exploration. 2009;102: 123–136. doi:10.1016/j.gexplo.2009.01.001

64. Moreiras Reynaga DK, Millaire J-F, Chávez Balderas X, Román Berrelleza JA, López Luján L, Longstaffe FJ. Residential patterns of Mexica human sacrifices at Mexico-Tenochtitlan and Mexico-Tlatelolco: Evidence from phosphate oxygen isotopes. Journal of Anthropological Archaeology. 2021;62: 101296. doi:10.1016/j.jaa.2021.101296

65. Knudson KJ, Price TD. Utility of multiple chemical techniques in archaeological residential mobility studies: case studies from Tiwanaku- and Chiribaya-affiliated sites in the Andes. Am J Phys Anthropol. 2007;132: 25–39. doi:10.1002/ajpa.20480

66. Bataille CP, Crowley BE, Wooller MJ, Bowen GJ. Advances in global bioavailable strontium isoscapes. Palaeogeography, Palaeoclimatology, Palaeoecology. 2020;555: 109849. doi:10.1016/j.palaeo.2020.109849

67. Bataille CP, Brennan SR, Hartmann J, Moosdorf N, Wooller MJ, Bowen GJ. A geostatistical framework for predicting variability in strontium concentrations and isotope ratios in Alaskan rivers. Chemical Geology. 2014;389: 1–15. doi:10. 1016/j.chemgeo.2014.08.030

68. Bataille CP, Bowen GJ. Mapping 87Sr/86Sr variations in bedrock and water for large scale provenance studies. Chemical Geology. 2012;304–305: 39–52. doi:10.1016/j.chemgeo.2012.01.028

69. Laffoon JE, Davies GR, Hoogland MLP, Hofman CL. Spatial variation of biologically available strontium isotopes (87Sr/86Sr) in an archipelagic setting: a case study from the Caribbean. Journal of Archaeological Science. 2012;39: 2371–2384. doi:10.1016/j.jas.2012.02.002

70. West JB, Hurley JM, Dudás FÖ, Ehleringer JR. The Stable Isotope Ratios of Marijuana. II. Strontium Isotopes Relate to Geographic Origin. Journal of Forensic Sciences. 2009;54: 1261–1269. doi:10.1111/j.1556-4029.2009.01171.x

71. Le Corre M, Dargent F, Grimes V, Wright J, Côté SD, Reich MS, et al. An ensemble machine learning bioavailable strontium isoscape for Eastern Canada. Geiss CE, editor. FACETS. 2025;10: 1–17. doi:10.1139/facets-2024-0180

72. Wang X, Bocksberger G, Arandjelovic M, Agbor A, Angedakin S, Aubert F, et al. Strontium isoscape of sub-Saharan Africa allows tracing origins of victims of the transatlantic slave trade. Nat Commun. 2024;15: 10891. doi:10.1038/s41467-024-55256-0

73. Blum JD, Taliaferro EH, Weisse MT, Holmes RT. Changes in Sr/Ca, Ba/Ca and 87Sr/86Sr ratios between trophic levels in two forest ecosystems in the northeastern U.S.A. Biogeochemistry. 2000;49: 87–101.

74. de Cserna Z. An outline of the geology of Mexico. In: Bally AW, Palmer AR, editors. The geology of North America--an overview. Boulder, CO: Geological Society of America; 1989. pp. 233–264.

75. Ferrusquía-Villafranca I. Geology of Mexico: a synopsis. In: Ramamoorthy TP, Bye R, Lot A, Fa J, editors. Biological diversity of Mexico: origins and distribution. New York: Oxford University Press; 1993. pp. 3–107.

76. Morán-Zenteno D. The geology of the Mexican republic. Cincinnati, OH: American Association of Petroleum Geologists; 1994.

77. Reynolds AC, Quade J, Betancourt JL. Strontium isotopes and nutrient sourcing in a semi-arid woodland. Geoderma. 2012;189–190: 574–584. doi:10.1016/j.geoderma.2012.06.029

78. Feinman GM, Nicholas LM. Perspectives on dietary variability in the Classic-period Valley of Oaxaca. In: Pérez Rodríguez V, Morell-Hart S, King SM, editors. Mesquite pods to mezcal: 10,000 years of Oaxacan cuisines. Austin, TX: University of Texas Press; 2024. pp. 99–122.

79. Grimstead DN, Nugent S, Whipple J. Why a standardization of strontium isotope baseline environmental data is needed and recommendations for methodology. Advances in Archaeological Practice. 2017;5: 184–195. doi:10.1017/aap.2017.6

80. Runia LexT. Strontium and calcium distribution in plants: effect on palaeodietary studies. Journal of Archaeological Science. 1987;14: 599–608. doi:10.1016/0305-4403(87)90078-1

81. Romaniello SJ, Field MP, Smith HB, Gordon GW, Kim MH, Anbar AD. Fully automated chromatographic purification of Sr and Ca for isotopic analysis. J Anal At Spectrom. 2015;30: 1906–1912. doi:10.1039/C5JA00205B

82. National Institute of Standards and Technology, editor. Certificate of Analysis Standard Reference Material 987. Department of Commerce, United States of America; 2007.

83. Ma J, Wei G, Liu Y, Ren Z, Xu Y, Yang Y. Precise measurement of stable (δ 88/86Sr) and radiogenic (87Sr/86Sr) strontium isotope ratios in geological standard reference materials using MC-ICP-MS. Chin Sci Bull. 2013;58: 3111–3118. doi:10.1007/s11434-013-5803-5

84. Galler P, Limbeck A, Boulyga SF, Stingeder G, Hirata T, Prohaska T. Development of an on-line flow injection Sr/matrix separation method for accurate, high-throughput determination of Sr isotope ratios by multiple collector-inductively coupled plasma-mass spectrometry. Anal Chem. 2007;79: 5023–5029. doi:10.1021/ac070307h

85. Aubert D, Probst A, Stille P, Viville D. Evidence of hydrological control of Sr behavior in stream water (Strengbach catchment, Vosges mountains, France). Applied Geochemistry. 2002;17: 285–300. doi:10.1016/S0883-2927(01)00080-4

86. Tricca A, Stille P, Steinmann M, Kiefel B, Samuel J, Eikenberg J. Rare earth elements and Sr and Nd isotopic compositions of dissolved and suspended loads from small river systems in the Vosges mountains (France), the river Rhine and groundwater. Chemical Geology. 1999;160: 139–158.

87. Wadleigh MA, Veizer J, Brooks C. Strontium and its isotopes in Canadian rivers: Fluxes and global implications. Geochimica et Cosmochimica Acta. 1985;49: 1727–1736. doi:10.1016/0016-7037(85)90143-7

88. Brun P, Zimmermann NE, Hari C, Pellissier L, Karger DN. CHELSA-BIOCLIM+ A novel set of global climate-related predictors at kilometre-resolution. EnviDat; 2022. p. 13194139533312 bytes, 423097 bytes. doi:10.16904/ENVIDAT.332

89. Karger DN, Conrad O, Böhner J, Kawohl T, Kreft H, Soria-Auza RW, et al. Climatologies at high resolution for the earth's land surface areas. Sci Data. 2017;4: 170122. doi:10.1038/sdata.2017.122

90. Hengl T, Jesus JM de, Heuvelink GBM, Gonzalez MR, Kilibarda M, Blagotić A, et al. SoilGrids250m: Global gridded soil information based on machine learning. PLOS ONE. 2017;12: e0169748. doi:10.1371/journal.pone.0169748

91. Hartmann J, Moosdorf N. The new global lithological map database GLiM: A representation of rock properties at the Earth surface. Geochemistry, Geophysics, Geosystems. 2012;13. doi:10.1029/2012GC004370

92. Mooney WD, Laske G, Masters TG. CRUST 5.1: A global crustal model at 5° × 5°. Journal of Geophysical Research: Solid Earth. 1998;103: 727–747. doi:10.1029/97JB02122

93. Gelaro R, McCarty W, Suárez MJ, Todling R, Molod A, Takacs L, et al. The Modern-Era Retrospective Analysis for Research and Applications, Version 2 (MERRA-2). 2017 [cited 17 Mar 2025]. doi:10.1175/JCLI-D-16-0758.1

94. Chipman HA, George EI, McCulloch RE. BART: Bayesian additive regression trees. The Annals of Applied Statistics. 2010;4: 266–298. doi:10.1214/09-AOAS285

95. Freiwald C. Maya migration networks: Reconstructing population movement in the Belize River valley during the Late and Terminal Classic. Ph.D., The University of Wisconsin - Madison. 2011. Available: https://www.proquest.com/docview/886460450/abstract/F82F1B17A9DA43E7PQ/1

Supporting Information

S1 Fig. PCA results identifying the major environmental factors driving strontium isotope patterns in Mesoamerica.

S1 Appendix. Identification of contamination from agricultural fertilizers in modern plant samples from San Martín Tilcajete.

S2 Appendix. Spanish translation of manuscript.

Fig 1 Click here to access/download;Figure;Fig 1.tif

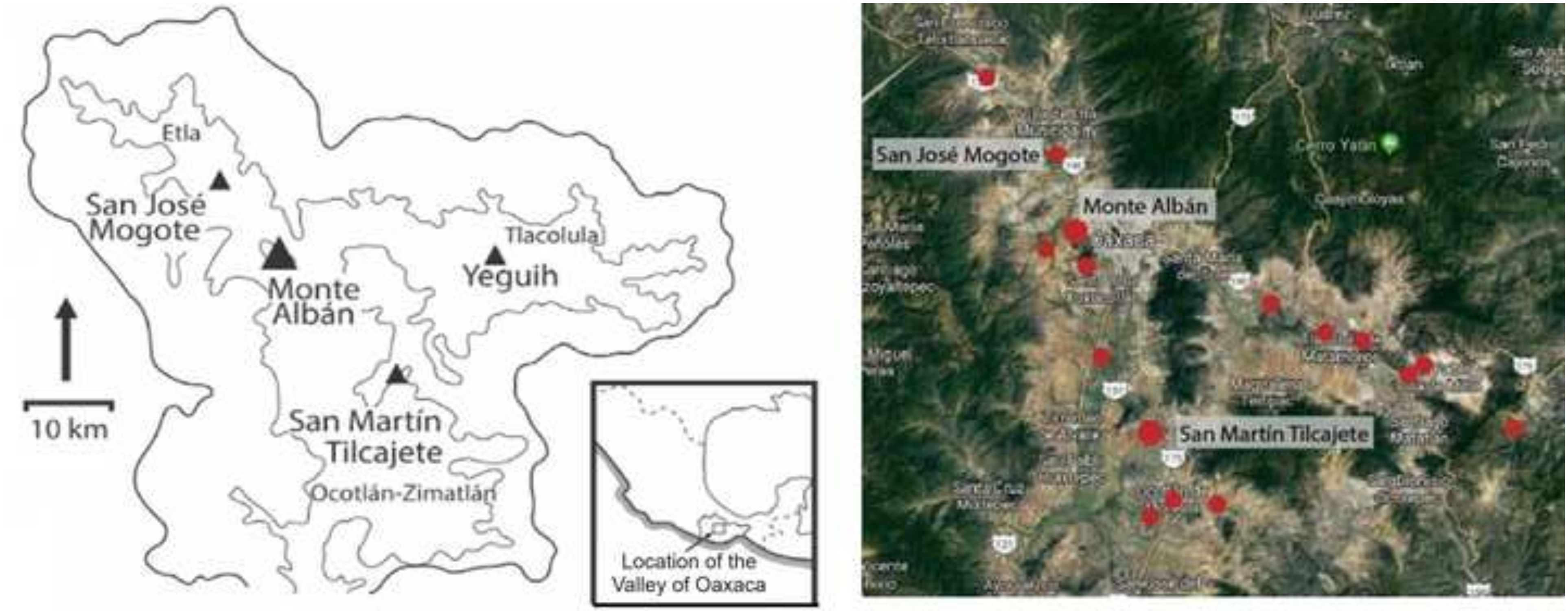

Fig 2

Click here to access/download;Figure;Fig 2.tif

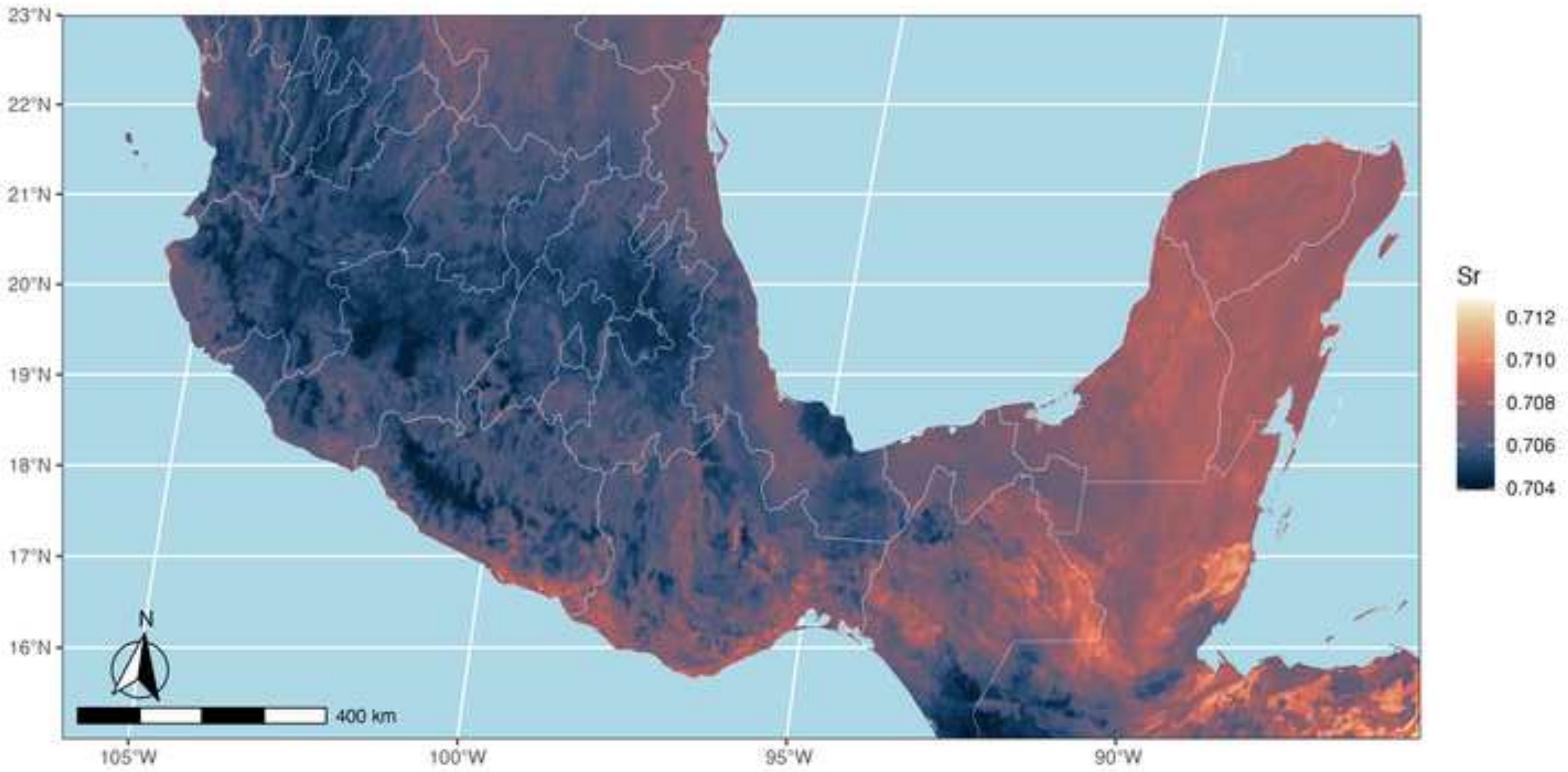

Fig 3

Click here to access/download;Figure;Fig 3.tif

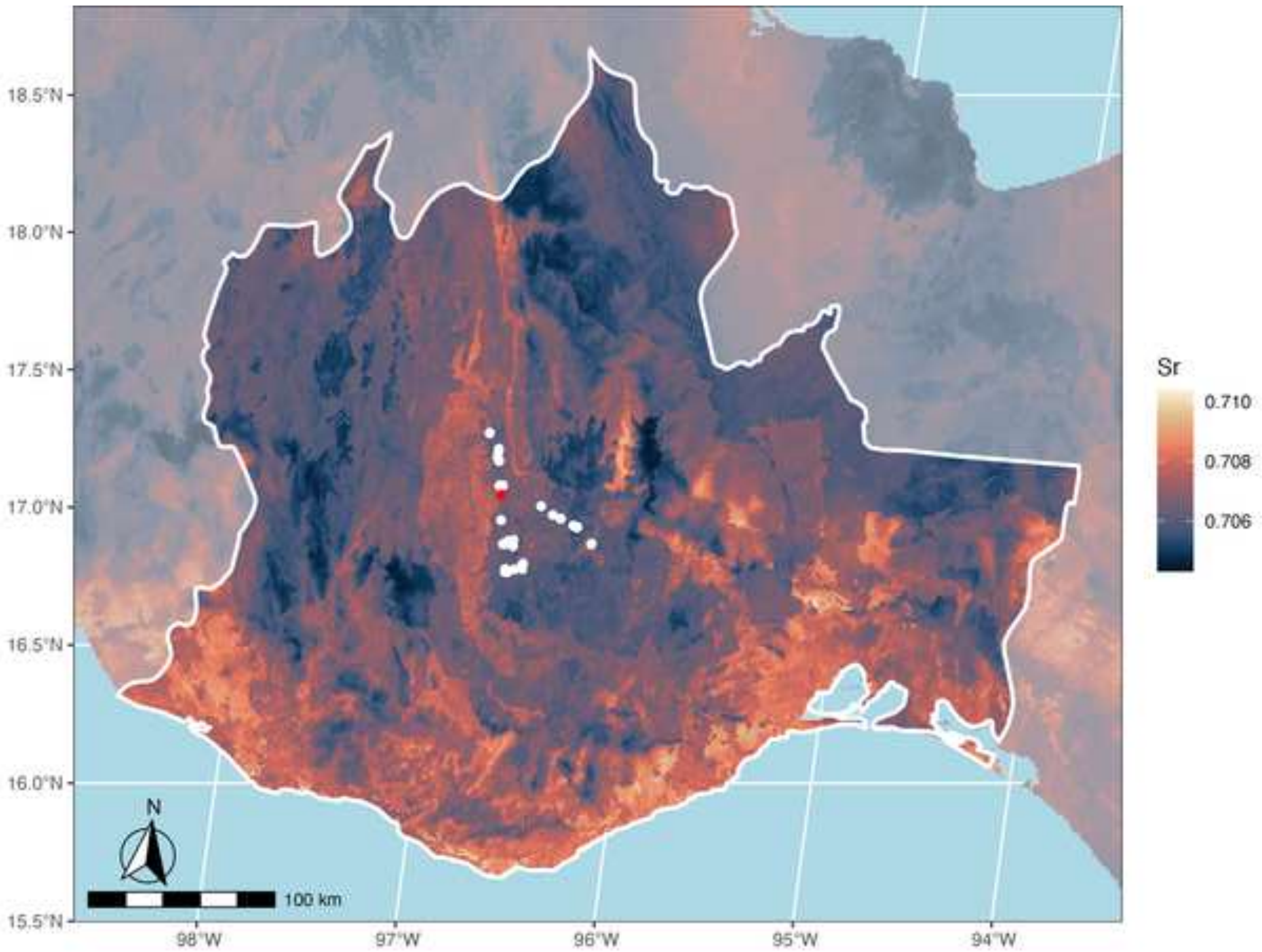

Click here to access/download;Figure;Fig 4.tif

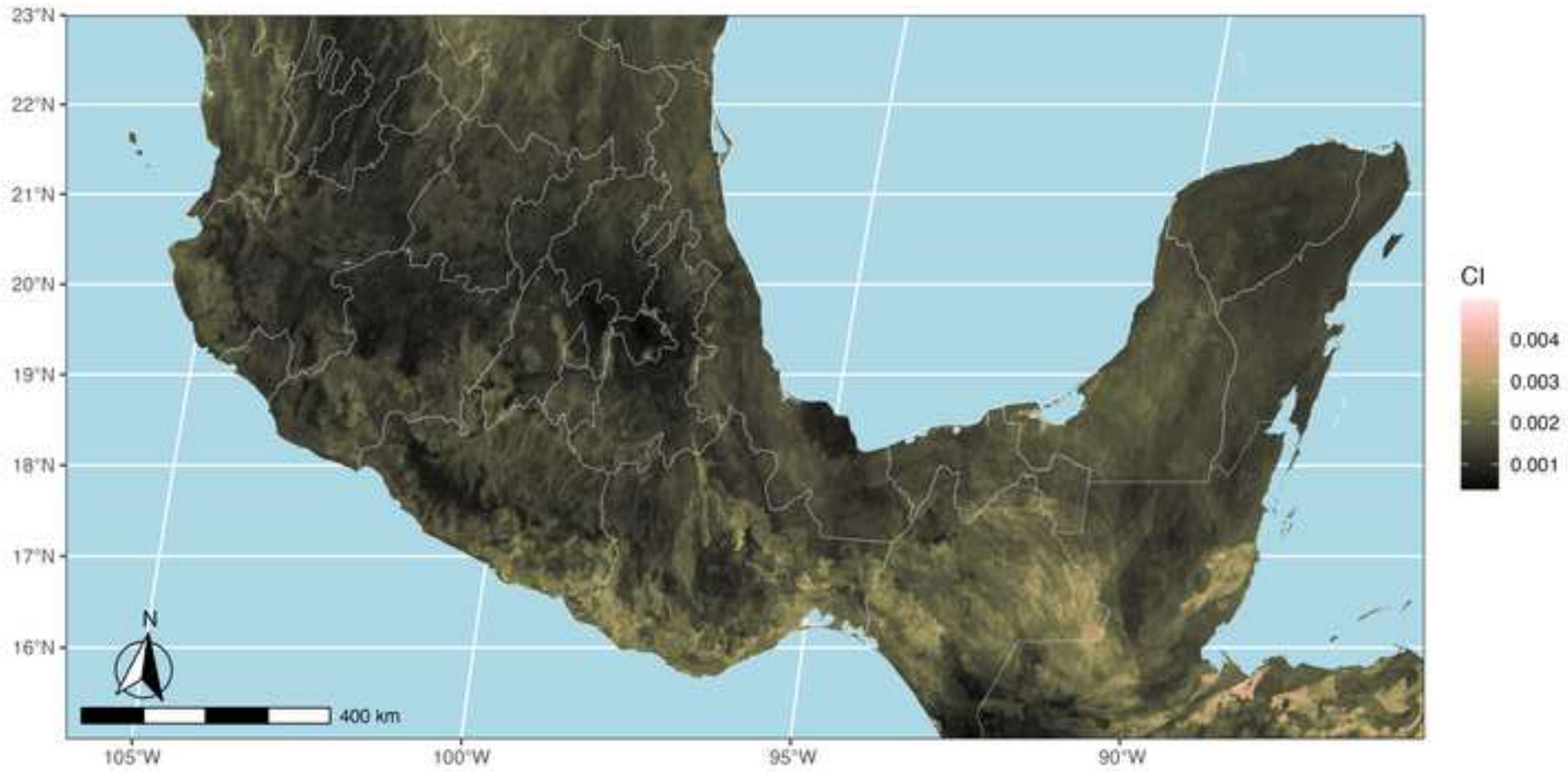

Fig 5

Click here to access/download;Figure;Fig 5.tif

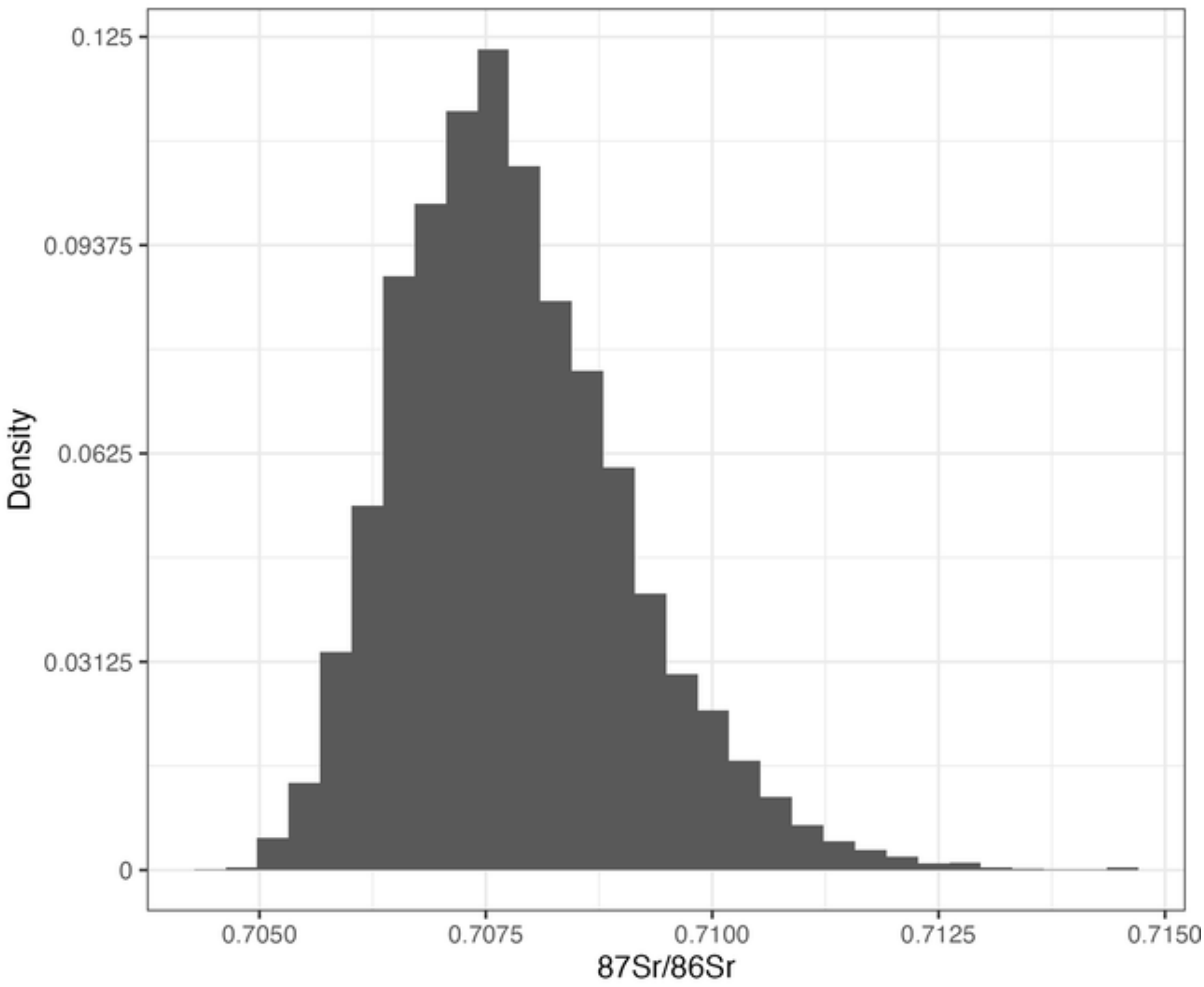

Fig 6

Click here to access/download;Figure;Fig 6.tif

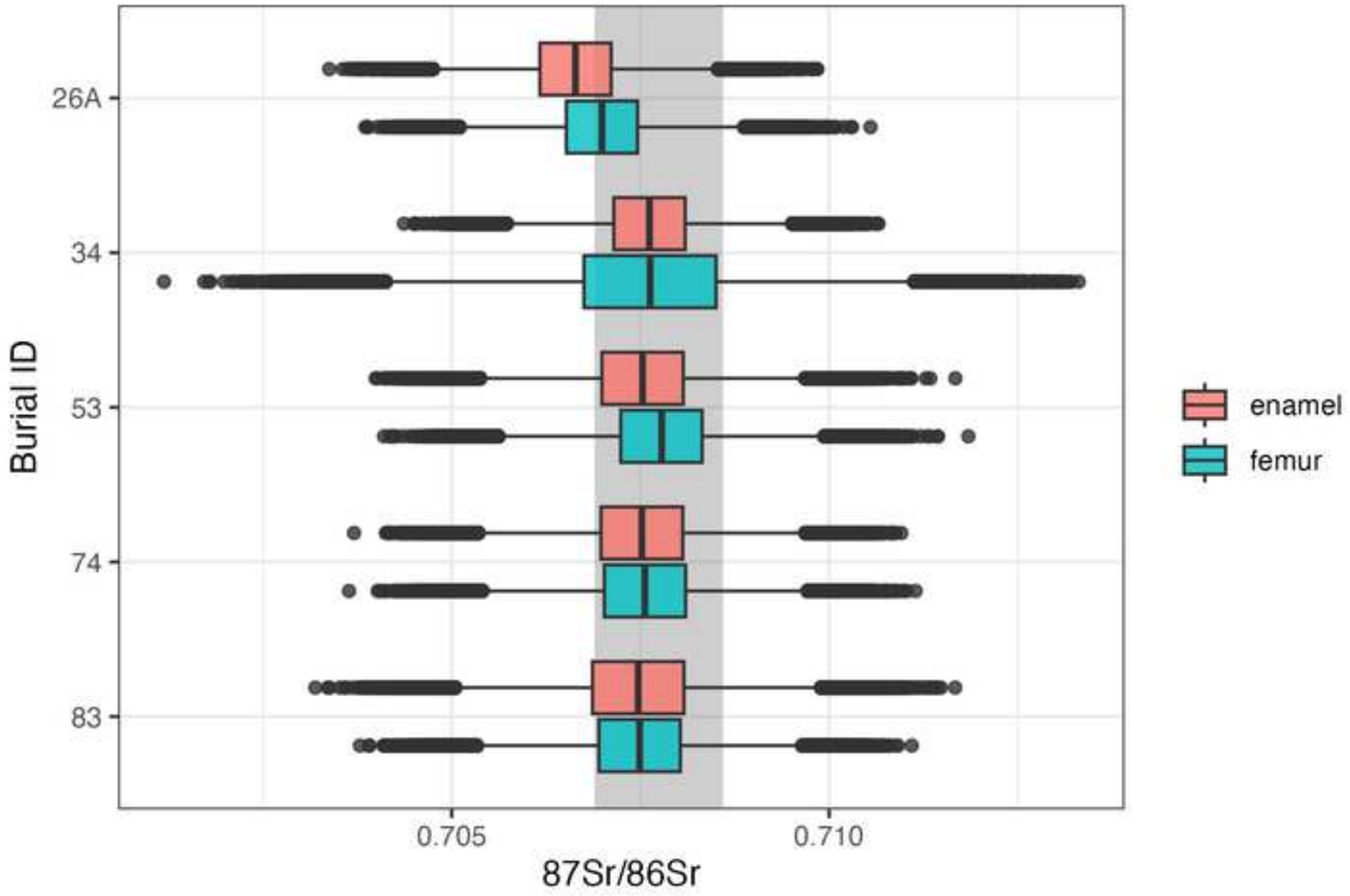

Click here to access/download
Supporting Information
S1 Appendix - Tilcajete fertilizer contamination.docx

Click here to access/download
Supporting Information
S2 Appendix - Spanish Translation.docx

S1 Fig

Click here to access/download
Supporting Information
S1 Fig - Predictor variable processing.docx
