# Supplementary material for: Radiogenic strontium isotope variability in the Valley of Oaxaca: A predictive isoscape for Mesoamerican paleomobility studies
Source: PLoS One. 2025 Dec 10;20(12):e0338628. doi: 10.1371/journal.pone.0338628 (PMC12694802; doi:10.1371/journal.pone.0338628)
Supplement: Attachment — Submitted filename: Response to Reviewers.docx [file pone.0338628.s008.docx]

**Academic Editor Comments**

1. I agree with your point that standard Random Forest (RF) models do not inherently provide uncertainty estimates. However, specific extensions of RF, such as Quantile Regression Forests, have been developed to address this limitation. In the paper, you reference two seminal works by Bataille and co-authors that present isoscape maps without including spatial uncertainty. However, more recent studies have explicitly addressed this issue. Therefore, I recommend a more detailed review of the isoscape literature—particularly with regard to machine learning approaches—and the inclusion of relevant and recent publications that account for uncertainty in spatial predictions.

*We have expanded our isoscape literature review section to include recent work on the use of quantile regression forests to quantify spatial uncertainty in isoscapes. We have also referenced this approach in our discussion of the advantages of our BART approach to isoscape modeling, primarily in its handling of uncertainty in data-sparse regions.*

2. In Figure 6, you present simulated draws based on the mean and standard deviation of published data, which are then compared to the expected local baseline from the isoscape. I believe it would be important to clearly explain the rationale and methodology behind generating these simulated data. This is especially relevant since the isotopic variability represented by a single sample appears to be significantly larger than any analytical uncertainty. Additionally, it is unclear why you did not simply use the average value along with its associated standard error, which might offer a more straightforward and transparent comparison.

*We have edited this figure to use the published ^87^Sr/^86^Sr value and its associated standard error rather than the simulated data.*

**Reviewer 1 Comments**

1. Table 2: add errors to Sr isotopic ratios.

*We have added 2 SE to each ^87^Sr/^86^Sr observation in Table 2.*

2. The geological context of the Oaxaca Valley is a little poor. Expanding it and also discussing the isotopic data related to the geology could help to better constrain the isotopic variation found in the area. A geological map of the Oaxaca Valley could be useful to understand the geological complexity of the region.

*We have expanded our discussion of the geological context of the Valley of Oaxaca and integrated discussions of the geology into our discussion of isoscape ^87^Sr/^86^Sr predictions across the Valley. Additionally, we have included a geological map of the Valley of Oaxaca in manuscript (Fig 2).*

3. Why don't you try to make the model using only the Mesoamerican and Oaxaca valley data, being the North and South America data extremely far?

*We tested models trained exclusively on Mesoamerican data and found essentially identical performance on held-out Oaxacan samples compared to models including all North and South American data. However, we retained the continental-scale training data because geographically distant sites are not necessarily distant in environmental feature space—a Precambrian terrain in Brazil may be more similar to parts of the Oaxacan highlands than geographically proximate volcanic areas. The expanded dataset provides greater environmental parameter coverage without degrading local performance, potentially improving model robustness for out-of-sample predictions across the full range of geological and climatic conditions relevant to archaeological applications in our study region.*

4. Some locations you have sampled show a huge isotopic range as, for example, the Monte Albán, with variation of 0.00055. How do you explain this?

*Local ^87^Sr/^86^Sr can vary a great deal even within a relatively small geographical area. Countless studies, as well as our own isoscape model (S1 Fig), show that the age of the underlying geologic bedrock is the primary driver of variation in bioavailable ^87^Sr/^86^Sr. Given that the Valley of Oaxaca is located in the most geologically complex region of Mexico and contains some of the oldest Precambrian formations in Mexico that are overlain with isolated Paleozoic, Mesozoic, and Cenozoic formations, it is expected that there would be greater variability in local ^87^Sr/^86^Sr values, even within sites.*

*Furthermore, local bioavailable ^87^Sr/^86^Sr values are also influenced by environmental factors such as differential weathering and the addition of atmospheric sediments such as windblown dust, volcanic tephra, and aerosolized sea salt in sea spray (see reviews in Bentley 2006; Blum et al, 2000). Attempting to capture this variation through plants adds an additional layer of variation, as not all ^87^Sr/^86^Sr is bioavailable to plants, and plant rooting depths determine what proportion of bedrock vs environmental ^87^Sr/^86^Sr will make up a plant’s own ^87^Sr/^86^Sr value (Stewart et al., 1998; Poszwa et al., 2002; Poszwa et al., 2004). For this reason, we sampled plants of various rooting depths to more accurately represent local bioavailable ^87^Sr/^86^Sr.*

*Despite these sources of expected variation, Monte Albán’s local range of ^87^Sr/^86^Sr variation is in line with the majority of the sites sampled in the study. The 16 sites across the Valley of Oaxaca demonstrate an average local ^87^Sr/^86^Sr range of 0.000482. This average excludes San Martín Tilcajete, whose samples showed a ^87^Sr/^86^Sr range an order of magnitude larger (0.004739) due to contamination from agricultural fertilizer use (discussed in S1 Appendix).*

*Local ^87^Sr/^86^Sr ranges of ~0.00050 are also fairly typical elsewhere in Mesoamerica. In a study characterizing baseline ^87^Sr/^86^Sr variability in central Mexico through analysis of environmental samples (plants, soils, water, fauna), Pacheco-Forés et al (2020) found a similar average local ^87^Sr/^86^Sr range of 0.00046 in 13 sites across central Mexico. Similarly, in a study of ^87^Sr/^86^Sr variation at the site of Copán in Honduras, Price et al (2010) found a local ^87^Sr/^86^Sr range of ~0.00040 (plants, water, fauna).*

*While the Valley of Oaxaca site ^87^Sr/^86^Sr ranges thus may be slightly higher than those observed elsewhere in Mesoamerica, the ranges are not so large as to call into question the validity of the observed ^87^Sr/^86^Sr values nor to imply they could be contaminated with agricultural fertilizers as the San Martín Tilcajete samples were.*

*Bentley, R. Alexander. “Strontium Isotopes from the Earth to the Archaeological Skeleton: A Review.” Journal of Archaeological Method and Theory 13, no. 3 (July 29, 2006): 135–87.* [*https://doi.org/10.1007/s10816-006-9009-x*](https://doi.org/10.1007/s10816-006-9009-x)*.*

*Blum, Joel D., E. Hank Taliaferro, Marie T. Weisse, and Richard T. Holmes. “Changes in Sr/Ca, Ba/Ca and 87Sr/86Sr Ratios between Trophic Levels in Two Forest Ecosystems in the Northeastern U.S.A.” Biogeochemistry 49 (2000): 87–101.*

*Pacheco-Forés, Sofía I., Gwyneth W. Gordon, and Kelly J. Knudson. “Expanding Radiogenic Strontium Isotope Baseline Data for Central Mexican Paleomobility Studies.” PLOS ONE 15, no. 2 (February 24, 2020): e0229687.* [*https://doi.org/10.1371/journal.pone.0229687*](https://doi.org/10.1371/journal.pone.0229687)*.*

*Poszwa, Anne, Etienne Dambrine, Bruno Ferry, Benoît Pollier, and Michel Loubet. “Do Deep Tree Roots Provide Nutrients to the Tropical Rainforest?” Biogeochemistry 60, no. 1 (August 1, 2002): 97–118.* [*https://doi.org/10.1023/A:1016548113624*](https://doi.org/10.1023/A:1016548113624)*.*

*Poszwa, Anne, Bruno Ferry, Etienne Dambrine, Benoît Pollier, Tonie Wickman, Michel Loubet, and Kevin Bishop. “Variations of Bioavailable Sr Concentration and 87Sr/86Sr Ratio in Boreal Forest Ecosystems.” Biogeochemistry 67, no. 1 (January 1, 2004): 1–20.* [*https://doi.org/10.1023/B:BIOG.0000015162.12857.3e*](https://doi.org/10.1023/B:BIOG.0000015162.12857.3e)*.*

*Price, T. Douglas, James H. Burton, Robert J. Sharer, Jane E. Buikstra, Lori E. Wright, Loa P. Traxler, and Katherine A. Miller. “Kings and Commoners at Copan: Isotopic Evidence for Origins and Movement in the Classic Maya Period.” Journal of Anthropological Archaeology 29, no. 1 (March 2010): 15–32.* [*https://doi.org/10.1016/j.jaa.2009.10.001*](https://doi.org/10.1016/j.jaa.2009.10.001)*.*

*Stewart, Brian W, Rosemary C Capo, and Oliver A Chadwick. “Quantitative Strontium Isotope Models for Weathering, Pedogenesis and Biogeochemical Cycling.” Geoderma 82, no. 1 (February 1, 1998): 173–95.* [*https://doi.org/10.1016/S0016-7061(97)00101-8*](https://doi.org/10.1016/S0016-7061(97)00101-8)*.*

5. As for the example you provide of Monte Albán, the isoscape prediction of a range from 0.7069 to 0.7086 is also huge. Is this range calculated for a single 1 km cell or from a larger area? Is it related to a particular geological complexity of the site or from other factors?

*We have clarified that the isoscape’s predicted local range for Monte Albán is calculated for a single 1 km cell. The ^87^Sr/^86^Sr range of this 1 km cell is indeed fairly large (0.00170), especially when compared to the ^87^Sr/^86^Sr range observed in modern plant samples from Monte Albán (0.00055). However, as we discussed in the manuscript, the isoscape’s wider predicted local ^87^Sr/^86^Sr range for Monte Albán more accurately reflects actual geologic and environmental inputs to local ^87^Sr/^86^Sr values as opposed to the idiosyncratic and opportunistic sampling of modern plants across a limited subsection of the contemporary archaeological site that produced an artificially narrow local ^87^Sr/^86^Sr range. The isoscape takes into account the age and composition of the underlying geologic bedrock as well as environmental Sr inputs, including the overall aridity, dry vs wet atmospheric aerosol deposition, elevation, and other factors. These factors, along with others that drive the isoscape model’s baseline predictions, are summarized in the supplemental document S1 Fig.*

*Additionally, the isoscape’s predicted ^87^Sr/^86^Sr range represents a conservative approximation of “local” Monte Albán ^87^Sr/^86^Sr values. Although we based our prediction on a single 1km cell from the model, archaeological surveys of the site show that it was approximately 3 sq km in size (Blanton 1978: Fig 1.3). While Nicholas and Feinman (2022) propose 1-2km as a reasonable catchment area for agricultural subsistence for most prehispanic settlements within the Valley of Oaxaca, they conclude that Monte Albán likely drew from beyond a 2km radius to fully meet its subsistence needs (p10). While we cannot know from where exactly Monte Albán was drawing its food supply (and thus its residents ^87^Sr/^86^Sr intake), the wider 1 km predicted isoscape range certainly represents a more realistic characterization of expected local ^87^Sr/^86^Sr values in ancient residents of Monte Albán than the narrower local range based solely on modern plant samples.*

*Blanton, Richard E. Monte Albán: Settlement Patterns at the Ancient Zapotec Capital. New York, NY: Academic Press, 1978.*

*Nicholas, Linda M., and Gary M. Feinman. “The Foundation of Monte Albán, Intensification, and Growth: Coactive Processes and Joint Production.” Frontiers in Political Science 4 (March 8, 2022): 805047.* [*https://doi.org/10.3389/fpos.2022.805047*](https://doi.org/10.3389/fpos.2022.805047)*.*

6. If my interpretation is correct, the 'measurement' of archaeological literature samples you use to test the model from Albàn site and reported in Fig. 6 as boxplot were simulated. I doubt that during the single analysis, the ^87^Sr/^86^Sr isotopic ratio varies as much as you report. I suggest you to use only the published values and associated errors in the absence of raw data.

*We have edited this figure to use the published ^87^Sr/^86^Sr value and its associated standard error rather than the simulated data.*

7. In Fig. 2 and 3 write ^87^Sr/^86^Sr and not Sr on the legend.

*Fixed.*

**Reviewer 2 Comments**

1. I understand the complexities of running isoscape models to produce the maps presented in the manuscript, and this is a large hurdle for non-experts wanting to use constructed isoscapes. You present the new data, but not the compiled data used. Also, I do not see the scripts used for the modeling included. These are invaluable for future researchers hoping to similarly construct isoscapes and use these following your approach. Could these be included as supplementary information?

*We have provided both the compiled data used in the model as well as the scripts for the modeling here:* [*https://github.com/flmnh-ai/oaxaca-isoscape*](https://github.com/flmnh-ai/oaxaca-isoscape)*. We included this in the data availability metadata when we submitted our manuscript and will confirm with the editor that the link will appear in the final publication.*

2. Line 27: which regions?

*We have specified that these two regions are the Teotihuacan Valley and the Maya region.*

3. Line 212: typo “some of”

*Corrected.*

4. Line 384 “Isoscape model performance”: for non-expert readers, it would be good to quickly define what “good” looks like in terms of RMSE and R2. Many people interested in isoscapes are not experts in statistics but understanding these performance evaluations is critical.

*We have added additional context to the methods and results sections to clarify interpretation of the RMSE and R2 metrics.*

5. Fig 2 & 3: “^87^Sr/^86^Sr” should be in the legend of the figure, not “Sr” which is confusing and might be interpreted to mean elemental Sr abundance. At present this doesn’t make sense.

*Fixed.*

6. Fig 4: The legend should indicate–or the figure caption–the the CI intervals in the legend are in terms of ^87^Sr/^86^Sr

*Fixed.*

7. Fig 5 & 6: “^87^Sr/^86^Sr” in the x-axis label

*Fixed.*
